# Supplementary material for: Bottleneck analysis of maternal and newborn health services in hard-to-reach areas of Bangladesh using ‘TANAHASHI’ framework’: An explanatory mixed-method study
Source: PLoS One. 2022 May 12;17(5):e0268029. doi: 10.1371/journal.pone.0268029 (PMC9098042; doi:10.1371/journal.pone.0268029)
Supplement: S1 File — (DOCX) [file pone.0268029.s001.docx]

Description of Tanahashi framework

A bottleneck is defined as a component of a system that limits the overall performance or capacity of the system. Identification of bottlenecks is critical for setting priorities on health system strengthening and thereby ensuring equity in maternal and child health services. Bottleneck analysis frame work was adapted from the Tanahashi’s health service coverage evaluation model.(24) In 1978, Tanahashi described a way of both measuring health service coverage and identifying bottlenecks from resource allocation to achievement of desired health outcome.(25, 26) This approach is useful in assessing equity in service coverage and identifying gaps to target interventions for vulnerable population.(27, 28) Different coverage indicators used in Tanahashi framework for measuring gaps within a defined target population are described below:

- *Availability:* The relationship of the volume and type of existing services to the volume of clients and their needs
- *Accessibility:* The relationship between the location of services and the location of clients
- *Utilization:* The relationship between the volume of initial contacts with the service and the volume of clients
- *Adequate Coverage:* The relationship between the volume of full services provided and the volume of clients
- *Effective Coverage:* The relationship between the volume of full services provided with quality and the volume of clients

These five domains of Tanahashi framework are measured against the total population which is considered as 100%. Thus, all these are represented as the proportion of the total population. The difference between total population and effective coverage measures the total gap (**Fig 1**) and the large differences between the consecutive domains depict the bottlenecks. The first two domains, i.e. Availability and Accessibility are measured from the determinants of supply side whereas Utilization and Adequate coverage are related to determinants from demand side. The final one (Effective coverage) are pertinent to determinants of quality of care.
