# Supplementary material for: Bottleneck analysis of maternal and newborn health services in hard-to-reach areas of Bangladesh using ‘TANAHASHI’ framework’: An explanatory mixed-method study
Source: PLoS One. 2022 May 12;17(5):e0268029. doi: 10.1371/journal.pone.0268029 (PMC9098042; doi:10.1371/journal.pone.0268029)
Supplement: S2 File — (DOCX) [file pone.0268029.s002.docx]

| **Sl No.** | **District** | **Upazila** | **Union** | **Population** | **HtR level** | **Physiographic Condition** |
| --- | --- | --- | --- | --- | --- | --- |
| 1 | Bandarban | Alikadam | Alikadam | 20977 | Extremely | Hilly |
| 2 | Bandarban | Bandarban Sadar | Rajbila | 7940 | Extremely | Hilly |
| 3 | Bandarban | Bandarban Sadar | Kuhalong | 11147 | Extremely | Hilly |
| 4 | Bandarban | Bandarban Sadar | Bandarban | 8529 | Extremely | Hilly |
| 5 | Bandarban | Bandarban Sadar | Sholaka | 8907 | Extremely | Hilly |
| 6 | Bandarban | Bandarban Sadar | Tankabati | 4325 | Extremely | Hilly |
| 7 | Bandarban | Lama | Aziznagar | 19525 | Extremely | Hilly |
| 8 | Bandarban | Naikhongchhari | Baishari | 13457 | Extremely | Hilly |
| 9 | Bandarban | Naikhongchhari | Dochhari | 6558 | Extremely | Hilly |
| 10 | Bandarban | Naikhongchhari | Ghandung | 14690 | Extremely | Hilly |
| 11 | Bandarban | Rowangchhari | Nowa Patang | 4105 | Extremely | Hilly |
| 12 | Bandarban | Rowangchhari | Alikhong | 5357 | Extremely | Hilly |
| 13 | Bandarban | Rowangchhari | Rowangchhari | 7370 | Extremely | Hilly |
| 14 | Bandarban | Rowangchhari | Tarachha | 8464 | Extremely | Hilly |
| 15 | Bandarban | Ruma | Ruma | 11973 | Extremely | Hilly |
| 16 | Bandarban | Ruma | Remakri Pransa | 5181 | Extremely | Hilly |
| 17 | Bandarban | Thanchi | Bali Para | 4741 | Extremely | Hilly |
| 18 | Bandarban | Thanchi | Thanchi | 6099 | Extremely | Hilly |
| 19 | Bandarban | Thanchi | Tindu | 3606 | Extremely | Hilly |
| 20 | Bandarban | Thanchi | Remakri Pransa | 4548 | Extremely | Hilly |
|  |  |  |  |  |  |  |
| **Sl No.** | **District** | **Upazila** | **Union** | **Population** | **HtR level** | **Physiographic Condition** |
| 1 | Sunamganj | Bishwambarpur | Fatehpur | 26412 | Extremely | Haor/wetland |
| 2 | Sunamganj | Bishwambarpur | Palash | 26844 | Very | Haor/wetland |
| 3 | Sunamganj | Bishwambarpur | Dakshin Badaghat | 20970 | Very | Haor/wetland |
| 4 | Sunamganj | Chhatak | Dakshin Islampur | 28271 | Extremely | Haor/wetland |
| 5 | Sunamganj | Chhatak | Noarai | 33091 | Very | Haor/wetland |
| 6 | Sunamganj | Chhatak | Chhatak | 10914 | Very | Haor/wetland |
| 7 | Sunamganj | Chhatak | Kalaruka | 32420 | Very | Haor/wetland |
| 8 | Sunamganj | Chhatak | Deokapan |  | Very | Haor/wetland |
| 9 | Sunamganj | Chhatak | Uttar Khurma | 16849 | Very | Haor/wetland |
| 10 | Sunamganj | Chhatak | Jawar Bazar | 30715 | Very | Haor/wetland |
| 11 | Sunamganj | Chhatak | Dakshin Khurma | 20806 | Very | Haor/wetland |
| 12 | Sunamganj | Chhatak | Saidergaon | 26095 | Very | Haor/wetland |
| 13 | Sunamganj | Chhatak | Saila Afzalabad | 28232 | Very | Haor/wetland |
| 14 | Sunamganj | Chhatak | Dular Bazar | 35938 | Very | Haor/wetland |
| 15 | Sunamganj | Chhatak | Sing Chapair | 22383 | Very | Haor/wetland |
| 16 | Sunamganj | Chhatak | Bhatgaon | 28583 | Very | Haor/wetland |
| 17 | Sunamganj | Derai | Rafinagar | 22317 | Extremely | Haor/wetland |
| 18 | Sunamganj | Derai | Charnar Char | 23742 | Extremely | Haor/wetland |
| 19 | Sunamganj | Derai | Kulanj | 26247 | Extremely | Haor/wetland |
| 20 | Sunamganj | Derai | Bhati Para | 19706 | Very | Haor/wetland |
| 21 | Sunamganj | Derai | Rajanagar | 22469 | Very | Haor/wetland |
| 22 | Sunamganj | Derai | Karimpur | 23718 | Very | Haor/wetland |
| 23 | Sunamganj | Derai | Jagaddal | 28570 | Very | Haor/wetland |
| 24 | Sunamganj | Derai | Derai Sarmangal | 14421 | Very | Haor/wetland |
| 25 | Sunamganj | Derai | Taral | 20140 | Very | Haor/wetland |
| 26 | Sunamganj | Dharampasha | Uttar Bangshikunda | 19617 | Extremely | Haor/wetland |
| 27 | Sunamganj | Dharampasha | Dakshin Bongshikunda | 24555 | Extremely | Haor/wetland |
| 28 | Sunamganj | Dharampasha | Chamardani | 20886 | Extremely | Haor/wetland |
| 29 | Sunamganj | Dharampasha | Madhyanagar | 20705 | Extremely | Haor/wetland |
| 30 | Sunamganj | Dharampasha | Joysree | 19219 | Extremely | Haor/wetland |
| 31 | Sunamganj | Dharampasha | Paikurati | 24966 | Extremely | Haor/wetland |
| 32 | Sunamganj | Dharampasha | Uttar Sukhair Rajapur | 16222 | Extremely | Haor/wetland |
| 33 | Sunamganj | Dharampasha | Dharmapasha | 26840 | Extremely | Haor/wetland |
|  |  |  |  |  |  |  |
| **Sl No.** | **District** | **Upazila** | **Union** | **Population** | **HtR level** | **Physiographic Condition** |
| 1 | Kurigram | Bhurungamari | Boldia | 23958 | Very | Char |
| 2 | Kurigram | Char Rajibpur | Kodailkati | 13090 | Extremely | Char |
| 3 | Kurigram | Char Rajibpur | Char Rajibpur | 33564 | Extremely | Char |
| 4 | Kurigram | Char Rajibpur | Mohanganj | 24602 | Extremely | Char |
| 5 | Kurigram | Chilmari | Raniganj | 20975 | Very | Char |
| 6 | Kurigram | Chilmari | Thanahat | 39772 | Very | Char |
| 7 | Kurigram | Chilmari | Ashtamir Char | 16129 | Very | Char |
| 8 | Kurigram | Kurigram Sadar | Punchgachhi | 24420 | Extremely | Char |
| 9 | Kurigram | Kurigram Sadar | Holokhana | 29004 | Very | Char |
| 10 | Kurigram | Kurigram Sadar | Jatrapur | 19519 | Very | Char |
| 11 | Kurigram | Nageshwari | Kedar | 24297 | Extremely | Char |
| 12 | Kurigram | Nageshwari | Ballabher Khas | 24013 | Extremely | Char |
| 13 | Kurigram | Nageshwari | Berubari | 18453 | Extremely | Char |
| 14 | Kurigram | Nageshwari | Narayanpur | 19922 | Extremely | Char |
| 15 | Kurigram | Nageshwari | Noonkhawa | 12385 | Extremely | Char |
| 16 | Kurigram | Nageshwari | Kachakata | 18303 | Very | Char |
| 17 | Kurigram | Rajarhat | Rajarhat | 34238 | Very | Char |
| 18 | Kurigram | Raumari | Bandaber | 47623 | Extremely | Char |
| 19 | Kurigram | Raumari | Saulmari | 25311 | Extremely | Char |
| 20 | Kurigram | Raumari | Raumari | 42945 | Extremely | Char |
| 21 | Kurigram | Raumari | Dantbhanga | 36482 | Very | Char |
| 22 | Kurigram | Ulipur | Begumganj | 15088 | Extremely | Char |
| 23 | Kurigram | Ulipur | Durgapur | 37141 | Extremely | Char |
| 24 | Kurigram | Ulipur | Hatia | 30798 | Extremely | Char |
| 25 | Kurigram | Ulipur | Tabakpur | 34688 | Extremely | Char |
| 26 | Kurigram | Ulipur | Bazra | 32207 | Extremely | Char |
| 27 | Kurigram | Ulipur | Buraburi | 29481 | Very | Char |
| 28 | Kurigram | Ulipur | Daldalia | 25268 | Very | Char |
| 29 | Kurigram | Ulipur | Dhamserni | 21079 | Very | Char |
|  |  |  |  |  |  |  |
| **Sl No.** | **District** | **Upazila** | **Union** | **Population** | **HtR level** | **Physiographic Condition** |
| 1 | Satkhira | Assasuni | Kulla | 24468 | Extremely | Coastal |
| 2 | Satkhira | Assasuni | Durgapur | 18647 | Extremely | Coastal |
| 3 | Satkhira | Assasuni | Budhhata | 27668 | Extremely | Coastal |
| 4 | Satkhira | Assasuni | Baradal | 30093 | Extremely | Coastal |
| 5 | Satkhira | Assasuni | Assasuni | 25104 | Extremely | Coastal |
| 6 | Satkhira | Assasuni | Khajra | 28526 | Extremely | Coastal |
| 7 | Satkhira | Assasuni | Anula | 24639 | Very | Coastal |
| 8 | Satkhira | Debhata | Parulia | 32222 | Extremely | Coastal |
| 9 | Satkhira | Debhata | Noapara | 29177 | Extremely | Coastal |
| 10 | Satkhira | Kaliganj | Nalta | 36246 | Extremely | Coastal |
| 11 | Satkhira | Kaliganj | Tarali | 22319 | Extremely | Coastal |
| 12 | Satkhira | Kaliganj | Champaphul | 16953 | Extremely | Coastal |
| 13 | Satkhira | Kaliganj | Dakshin | 18375 | Extremely | Coastal |
| 14 | Satkhira | Kaliganj | Mathureshpur | 30207 | Extremely | Coastal |
| 15 | Satkhira | Satkhira Sadar | Bali | 17503 | Extremely | Coastal |
| 16 | Satkhira | Satkhira Sadar | Labsa | 32248 | Extremely | Coastal |
| 17 | Satkhira | Satkhira Sadar | Shibpur | 19074 | Extremely | Coastal |
| 18 | Satkhira | Satkhira Sadar | Pourashava | 106395 | Extremely | Coastal |
| 19 | Satkhira | Satkhira Sadar | Brahma Rajpur | 21748 | Extremely | Coastal |
| 20 | Satkhira | Satkhira Sadar | Alipur | 28619 | Extremely | Coastal |
| 21 | Satkhira | Satkhira Sadar | Fingri | 32931 | Extremely | Coastal |
| 22 | Satkhira | Shyamnagar | Kashimari | 28769 | Extremely | Coastal |
| 23 | Satkhira | Shyamnagar | Padma Pukur | 25551 | Extremely | Coastal |
| 24 | Satkhira | Shyamnagar | Atulia | 33704 | Extremely | Coastal |
| 25 | Satkhira | Shyamnagar | Shyamnagar | 30106 | Extremely | Coastal |
| 26 | Satkhira | Shyamnagar | Ishwaripur | 35340 | Extremely | Coastal |
| 27 | Satkhira | Shyamnagar | Buri Goalini | 27167 | Extremely | Coastal |
| 28 | Satkhira | Shyamnagar | Kaikhali | 28350 | Extremely | Coastal |
| 29 | Satkhira | Shyamnagar | Ramjan Nagar | 25155 | Extremely | Coastal |
| 30 | Satkhira | Shyamnagar | Gabura | 36236 | Extremely | Coastal |
| 31 | Satkhira | Shyamnagar | Munshiganj | 36867 | Extremely | Coastal |
| 32 | Satkhira | Shyamnagar | Buri Goalini range |  | Extremely | Coastal |
| 33 | Satkhira | Tala | Khalishkhali | 29321 | Very | Beel/wetland |
| 34 | Satkhira | Tala | Khesra | 28619 | Very | Beel/wetland |
