# Supplementary material for: Bottleneck analysis of maternal and newborn health services in hard-to-reach areas of Bangladesh using ‘TANAHASHI’ framework’: An explanatory mixed-method study
Source: PLoS One. 2022 May 12;17(5):e0268029. doi: 10.1371/journal.pone.0268029 (PMC9098042; doi:10.1371/journal.pone.0268029)
Supplement: S3 File — (PDF) [file pone.0268029.s003.pdf]

Annex 4: Data collection tools

Tool 1: Facility assessment tools (maternal and neonatal health services)

Facility assessment tools (maternal and neonatal health services)

Information about observation

Date of observation: ☐☐-☐☐-☐☐☐☐ (D D |M M| Y Y Y Y)	Time of onset: ☐☐:☐☐ (24 hour)
Name of the observer : ______________________________	Code: ☐☐☐☐

Facility identification

001	Name and code of facility	________________________	☐☐☐☐☐ ☐
002	Address	____	
004	District	________________________	☐☐
005	Upazila	________________________	☐☐
006	Union	________________________	☐☐
007	Type of facility		
	Upazila health complex (UHC)		1
	Union health and child family planning welfare center (UH&FWC)		2
008	Ownership (management authority)		
	Government/public (MOHFW)		1
	Local government		2

Information of the respondents:

Name of the respondents	Designation	Phn no:
1.	______________________	
2.	______________________	
3.	______________________	
4.	______________________	
5.	______________________	

Module 1: General information and service availability

Section 1: General Service availability and in patient services

Service availability:

100	Does this facility offer any of the following client services? In other words, is there any location in this facility where clients can receive any of the following services:	**Yes**	**No**	**Comment**
01	Antenatal care (ANC) services	1	2	
02	Normal delivery	1	2	
03	Caesarean delivery (caesarean section)	1	2	
04	Laboratory diagnostic services	1	2	
05	Blood transfusion services	1	2	
06	Family planning services	1	2	

Services and basic amenities:

110	Does this facility provide in-patient care?	Yes	1	→112
		No	2	
111	Does this facility have beds for overnight observation?	Number of beds	☐☐☐	
112	Total number of beds in the facility have, both for adults and children (except delivery bed)	# of inpatient beds	☐☐☐	
		Don't know	98	

Laboratory services:

120	Check Q100.04, if laboratory services available or not	Yes	1	
		No	2	→next section

121	Check whether following tests can be done in this facility or not in last 6 months:	**Yes**	**No**
01	Blood grouping and typing	1	2
02	Urine albumin	1	2
03	Blood culture	1	2
04	Urine R/M/E	1	2
05	Ultra sonogram	1	2
06	X ray	1	2
07	Blood lipid profile	1	2
08	Fasting blood glucose	1	2
09	Rapid diagnostic test	1	2
10	Test to diagnosis the TORCH infection	1	2

Section 2: General filter questions

Storage of medicines:

200	Does this facility store or keep any medicines (including antibiotics, analgesics), vaccines or contraceptive commodities in the facility? I am referring to medicines/commodities that are meant to be given to clients when a provider prescribes them.	Yes	1	
		Stocks no medicine	2	
	CHECK Q100.06, if "yes" then ask:			
201	Are contraceptive commodities generally stored in the family planning service area, or are they stored in a common area with other medicines?	Stored in FP service area	1	
		Stored with other medicines	2	
		FP commodities not stocked	3	

Module 2: General service Readiness

Section 3: Hour Staff Coverage – Infrastructure, Communication, External Supervision, User Fees, Sources of Revenue

Infrastructure & 24- Hour staff coverage:

300	Outlook of the hospital as a whole	Poor (congested, very old building, no specification)	1	
		Satisfactory	2	
		Good	3	
		Excellent	4	
301	Overall cleanliness of the facility	Very clean	1	
		Mostly clean	2	
		Mostly dirty	3	
		Very dirty	4	
302	*Please observe the following points:*	**Yes**	**No**	
01	Specified parking area	1	2	
02	Availability of a reception desk at the entrance of the hospital	1	2	
03	Availability of a well-organized registration desk	1	2	
04	Specific waiting area with sitting arrangements	1	2	
05	Citizen Charter displayed in facility mentioning rights of mother and newborn	1	2	
06	Display board / Sign posts showing location and availability of services	1	2	
07	Availability of complain box	1	2	
08	Availability of well-organized dispensing counter for patients	1	2	
09	Adequate space for emergency, outdoor, pathology and indoor services	1	2	
303	Is there a health care worker present at the facility at all times, or officially on call for the facility at all times (24 hours a day) for emergencies? Specifically, I am referring to medical specialists, medical officers, nurses and paramedics.	Yes, staff present in 24 hour	1	
		No 24-hour staff don't present, but on call service present	2	→310
304	Is there a duty schedule or call list for 24-hour staff coverage?	Yes	1	
		Duty schedule not maintained	2	→310
305	Separate maternity ward	Yes	1	
		No	2	
306	Separate paediatric ward	Yes	1	
		No	2	

Communication:

310	Does this facility have a ***land line telephone*** that is available to call outside at all times client services are offered?<br><br>*CLARIFY THAT IF FACILITY OFFERS 24-HOUR EMERGENCY SERVICES, THEN THIS REFERS TO 24-HOUR AVAILABILITY*	Yes	1	
		No	2	→312
311	Is it functioning?	Yes	1	

	ACCEPT REPORTED RESPONSE	No	2	
312	Does this facility have a ***cellular telephone or a private cellular phone*** that is supported by the facility	Yes	1	
		No	2	→314
313	Is it functioning?<br><br>*ACCEPT REPORTED RESPONSE*	Yes	1	
		No	2	
314	Does this facility have ***a computer?***	Yes	1	
		No	2	→316
315	Is it functioning?<br><br>*ACCEPT REPORTED RESPONSE*	Yes	1	
		No	2	
316	Is there access to email or internet via computer and/or mobile phone within the facility?<br><br>*ACCEPT REPORTED RESPONSE.*	Yes	1	
		No	2	→320
317	Is the email or internet routinely available for ***at least 2 hours*** on days that client services are offered?<br><br>*ACCEPT REPORTED RESPONSE.*	Yes	1	
		No	2	

Source of water and power supply:

320	What is the **most commonly** used source of water **at this time**?	Piped into facility	01	
		Piped onto facility ground	02	
		Public tap/ standpipe	03	
		Tube well/borehole	04	
		Protected dug well	05	
		Unprotected dug well	06	
		Protected spring	07	
		Unprotected spring	08	
		Rainwater	09	
		Bottled water	10	
		Cart w/small tank/drum	11	
		Tanker truck	12	
		Surface water (river/dam/lake/pond)	13	
		Other (specify)____________________	96	
		Don't know	98	
		No water source	99	
321	Is there routinely a time of year when the facility has a severe shortage or lack of water?	Yes	1	
		No	2	
322	Is this facility connected to the national electricity grid?	Yes	1	
		No	2	→324
		Don't know	98	→324
323	During the past 7 days, was electricity (excluding any back-up generator) available during the times when the facility was open for services, or was it ever interrupted **for more than 2 hours at a time?**<br><br>*CONSIDER ELECTRICITY TO BE ALWAYS*	Always available	1	
		Sometimes interrupted.	2	
		Don't know	98	

	AVAILABLE<br>*IF INTERUPTED FOR LESS THAN 2 HOURS AT A TIME.*			
324	Does this facility have other sources of electricity, such as a generator or solar system?	Yes	1	
		No	2	→330
325	What other sources of electricity does this facility have?<br><br>*PROBE FOR ANSWERS AND CIRCLE ALL THAT APPLY*	Fuel-operated generator	A	
		Battery-operated generator	B	
		Solar system	C	→330
326	Is the generator functional?<br><br>*ACCEPT REPORTED RESPONSE*	Yes	1	
		No	2	

External supervision:

330	Does this facility receive any external supervision from the any upper level facility?	Yes	1	
		No	2	→334
331	When was the last time a supervisor from any upper level office came here on a supervisory visit? Was it within the past 6 months or more than 6 months ago?	Within the past 6 months	1	
		More than 6 months ago	2	→334
332	During the past 6 months, how many supervisory visits has this facility received from an upper level office?	No. of supervisory visits ☐☐		
		Don't know	98	
333	Does this facility maintain records of written comments made by supervisors from outside the facility when they make their supervisory visits?	Yes	1	
		No	2	
334	Now I would like to ask a few questions about community level supervision of community Health Workers. Does this facility have, or work with Community Health Workers (CHWs)?	Yes	1	
		Facility does not have CHW	2	→340
335	Do staff from this facility do community level supervision of the CHWs?	Yes	1	
		No	2	→340
336	Is there an annual community level supervision schedule created by health facility staff?	Yes	1	
		No	2	
337	How many supervision visits to community level in the past six months were carried out by health facility staff?	No. of supervisory visits ☐☐		
		Don't know	98	

Leadership and governance:

340	Is any internal supervision and monitoring of staff's performance done in this facility?	Yes	1	
		No	2	→343
341	Who is responsible for this internal monitoring?	Manager	1	
		UH&FPO	2	
		Civil surgeon	3	
		Others (specify)	96	
342	How frequently is this monitoring activities/ meeting held?	Monthly	1	
		Bi- monthly	2	
		Quarterly	3	
		Yearly	4	
343	Is there any public support to the management/Involvement of the community in	Yes	1	
		No	2	

	the management process			
344	Use of checklist for supervision and monitoring	Yes	1	
		No	2	
345	Any specific check list to measure quality of care in the facility	Yes	1	
		No	2	
346	Periodical Performance review	Yes	1	
		No	2	
347	Availability of job description and job performance according to job description	Yes	1	
		No	2	
348	Individual performance management system	Yes	1	
		No	2	
349	Regular visit to the different section by the concern manager	Yes	1	
		No	2	
350	If staffing levels do not meet the needs of the maternity or newborn wards, what actions are taken?	Re-assign staff	1	
		Opt for on-call staff	2	
		Contract-in staff	3	
351	What happens if a unit or person is performing exceptionally well? Are there rewards systems?	Reward as cash	1	
		Material award	2	
		Appraisal	3	
		Picture on wall	4	
		Up gradation of post	5	
		Nothing done	6	
		Others (specify)	96	
352	What happens if a unit or person is found not to be performing well?	Sanction	1	
		Reprimand	2	
		Discussion to find the causes	3	
		Nothing	4	
353	Availability of any committee in the facility	Yes	1	
		No	2	→355
354	What are these committees?<br><br>*READ OUT EACH RESPONSE CATEGORY AND CIRCLE ALL MENTIONED*	Infection prevention control program(IPC)	A	
		Quality improvement committee (QIc)	B	
		Any internal committee to monitor staff's performance	C	
		Others (specify)___________	Y	
355	Is task shifting done in this facility?	Yes	1	
		No	2	
	If Q354→A, ask next question, otherwise skip Q356.			
356	Is there any IPC focal person?	Yes	1	
		No	2	
357	Is there any system for regular meeting regarding infection control?	Yes	1	
		No	2	
358	Is there a complaint system?	Yes	1	
		No	2	→360
359	Is there any person involved in receiving complaints?	Yes	1	
		No	2	
360	Are community stakeholders involved in quality improvement efforts in your facility?	Yes	1	
		No	2	
361	Are there women's support groups in community	Yes	1	

	served by this facility?	No	2	→370
362	Do you have any formal link/communication with these groups?	Yes	1	
		No	2	

User fees:

370	Does this facility have any **routine user-fees or charges** for client services, including charges for health cards and for client registration? These could be routine fees for some services or for medicines.	Yes		1	
		No		2	→380
371	Does the facility charge a fixed fee that covers all services that a client receives, or are there separate fees for different components of the services provided by the facility?<br><br>*PROBE.*	Fixed fee covering all services		1	
		No charge fee for separate items		2	
372	Does this facility have a fee for the following items:<br><br>*READ OUT EACH RESPONSE CATEGORY AND CIRCLE APPROPRIATELY*	**Yes**	**No**		
01	Client health card	1	2		
02	Registration	1	2		
03	Consultation	1	2		
04	Medicines	1	2		
05	Vaccines (TT)	1	2		
06	Contraceptive commodities	1	2		
07	Normal deliveries	1	2		
08	Syringe and needles	1	2		
09	Caesarean section	1	2		
10	Laboratory tests	1	2		
373	Are the official fees posted or displayed so that the client can easily see them?	Yes		1	
		No		2	
374	Does this facility ever exempt clients from user fees? In other words, are any of this facility's clients exempted from user fees?	Yes		1	
		No		2	
		Don't know		98	
375	Does this facility follow any written guidelines on exemption of user fees?	Yes		1	
		No		2	

Sources of revenue:

380	Now, I would like to ask you about the sources of revenue or funding for this facility. Tell me if the facility received any revenue or funding from any of the listed resources during the session 2015-2016 financial year.<br><br>*CIRCLE ALL THAT APPLY. PROBE FOR EACH.*	Ministry of health	A	
		Others public ministries	B	
		Medical schemes (insurance)	C	
		Social security fund	D	
		Reimbursement by employer	E	
		Govt. contribution to private	F	
		Donor agencies/ NGOs	G	
		Faith-based	H	
		Community programs	I	

		User fees	J	
		None	X	
		Others (specify)____________________	Y	

General medicines and specific medicines for maternal and child health:

381	Are any of the following **medicines and logistics** available in the facility?	**Available always**	**Mostly available**	**Sometimes available**	**Never available**
01	Diclofenac tablets (50 mg) or sustained release tabs (100mg)	1	2	3	4
02	Paracetamol tablets	1	2	3	4
03	Paracetamol syrup or suspension	1	2	3	4
04	Paracetamol dispersible paediatric-dozed tablets	1	2	3	4
05	Indomethacin capsules	1	2	3	4
06	Drotaverine tablet	1	2	3	4
07	Diclofenac injection	1	2	3	4
08	Pethidine injection (100mg/2ml or 25mg/1ml)	1	2	3	4
09	Ibuprofen tablets	1	2	3	4
10	Normal saline / sodium chloride injectable solution	1	2	3	4
11	Ringers lactate (Hartsol)	1	2	3	4
12	5% dextrose - normal saline	1	2	3	4
13	Calcium gluconate injection	1	2	3	4
14	Folic acid tablets	1	2	3	4
15	Iron tablets	1	2	3	4
16	Iron + folic acid combination tablet	1	2	3	4
17	Magnesium sulphate injection	1	2	3	4
18	Misoprostol tablets/capsules	1	2	3	4
19	Oxytocin or other injectable uterotonic	1	2	3	4
20	Tetanus toxoid vaccine	1	2	3	4
21	Oral rehydration salts (ORS) sachets	1	2	3	4
22	Vitamin a capsules	1	2	3	4
23	Zinc dispersible tablets	1	2	3	4
24	Calcium lactate tablet	1	2	3	4
25	Ferrous sulphate + folic acid + zinc capsules	1	2	3	4
26	Ferrous fumerate + folic acid tablet	1	2	3	4
27	Zinc sulphate syrup	1	2	3	4
28	Methyl ergometrine injection	1	2	3	4
29	Ergometrine tablet	1	2	3	4

30	Gentian violet 1%	1	2	3	4
31	Nystatin drops	1	2	3	4
32	Single-use standard disposable syringes with needles or auto-destruct syringes with needles	1	2	3	4
33	Infusion set for iv solution	1	2	3	4
34	Cannula for administering iv fluids	1	2	3	4
35	Latex gloves	1	2	3	4
36	Alcohol-based hand rub	1	2	3	4
37	Hand washing soap	1	2	3	4
38	Disinfecting solution	1	2	3	4

Storage condition: Medicines

382	Observe the place where the medicines assessed so far are stored and indicate the presence (or absence) of each of the following storage conditions.	Yes	No
01	Medicines off the floor	1	2
02	Medicines protected from water	1	2
03	Medicines protected from the sun	1	2
04	Room clean of evidence of rodents (bats, rats) or pests (roaches, etc)?	1	2
05	The storage room well ventilated	1	2

383	Are the medicines organized according to date of expiration ("first expire, first out")?	Yes, all medicines	1	
		Yes, only some medicines	2	
		No	3	
384	What system does this facility use to monitor the amount of medicines received, the amount issued, and the amount present?	Computer system updated daily	1	
		Ledger/stock card updated daily	2	
		Computer system not updated daily, but there is daily record of distributed medicines.	3	
		Ledger /stock card not updated daily, but there is daily record of distributed medicines	4	
		Other system (specify) ___________________________	96	

Section 4: Staffing- management- Client opinion- Transport –HMIS and Health statistics

Staffing:

Please tell me: a) How many staff in each of the following qualification / occupational categories are sanctioned (expected) to work in this facility. b) For each qualification / occupational category, how many are currently posted and working in the facility (i.e., assigned to, employed by, or seconded to facility) in total, either full time or part time c) For each qualification / occupational category, among those currently posted and working in the facility, how many are part-time					
400	Name of the post	Sanctioned posts	Posted	# present on assessment day	Comment
01	Consultant (OBS. & GYN.)	☐☐	☐☐	☐☐	
02	Consultant (Paediatrics)	☐☐	☐☐	☐☐	
03	Consultant (Neonatology)	☐☐	☐☐	☐☐	
04	Medical Officer (Total)	☐☐	☐☐	☐☐	
05	Medical Officer (Maternity ward)	☐☐	☐☐	☐☐	
06	Medical Officer (Paediatrics)	☐☐	☐☐	☐☐	
07	Medical Officer (Neonatology)	☐☐	☐☐	☐☐	
08	Staff nurses (Total)	☐☐	☐☐	☐☐	
09	Staff nurses (Maternity ward)	☐☐	☐☐	☐☐	
10	Staff nurses (Paediatrics ward)	☐☐	☐☐	☐☐	
11	Staff nurses (Neonatology ward)	☐☐	☐☐	☐☐	
12	Other nurses	☐☐	☐☐	☐☐	
13	Anaesthetist	☐☐	☐☐	☐☐	
14	Midwives	☐☐	☐☐	☐☐	
15	Medical assistants (Total)	☐☐	☐☐	☐☐	
16	Medical assistants (Maternity ward)	☐☐	☐☐	☐☐	
17	Medical assistants (Paediatrics ward)	☐☐	☐☐	☐☐	
18	Medical assistants (Neonatology ward)	☐☐	☐☐	☐☐	
19	Other 1	☐☐	☐☐	☐☐	
20	Other 2	☐☐	☐☐	☐☐	
21	Other 3	☐☐	☐☐	☐☐	

Client opinion and feedback:

410	Does this facility have any system for determining clients' opinions about the health facility or its services?	Yes	1	
		No	2	→420
411	Please tell me all the methods that this facility uses to elicit client opinion<br><br>*CIRCLE ALL METHODS MENTIONED AND PROBE: ANY MORE?*	Suggestion box	A	
		Client survey form	B	
		Client interview form	C	
		Official meeting with community leaders	D	
		Informal discussion with clients or the community	E	
		EMAIL	F	
		Facility's website	G	
		Letters from clients/ community	H	
		Text/ SMS	I	
		Others (specify)_____________________	Y	
		Don't know	X	

Transport and emergencies:

420	Does this facility have a **functional ambulance** or other vehicle for emergency transportation for clients that is stationed at this facility and that operates from this facility?	Yes	1	
		No	2	→430
421	May I see the ambulance (or other vehicle)?	Observed	1	
		Reported but not seen	2	
422	Does this facility have access to an ambulance or other vehicle for emergency transportation for clients that is stationed at another facility or that operates from another facility?	Yes	1	
		No	2	

HMIS:

<table>
<tr><td rowspan="2">430</td><td rowspan="2" colspan="3">Does this facility have a system in place to regularly collect health services data?</td><td colspan="4">Yes</td><td>1</td><td colspan="2"></td></tr>
<tr><td colspan="4">No</td><td>2</td><td colspan="2">→440</td></tr>
<tr><td rowspan="3">431</td><td rowspan="3">Please tell me:
a) If this facility regularly compiles the following reports containing health services information
b) How frequently the reports are compiled, and
c) Finally, I would like to see a copy of the most recent one.</td><td colspan="2">(a)</td><td colspan="4">(b)</td><td colspan="2">(c)</td></tr>
<tr><td colspan="2">Report compiled(if yes→(b),(c)</td><td colspan="4">How frequently are reports compiled?</td><td colspan="2">Copy of recent report</td></tr>
<tr><td>Yes</td><td>No</td><td>Monthly or more often</td><td>Every 2-3 months</td><td>Every 4- 6 months</td><td>More than 6 months</td><td>observed</td><td>Reported, not seen</td></tr>
<tr><td>01</td><td>Hospital activity report</td><td>1</td><td>2</td><td>1</td><td>2</td><td>3</td><td>4</td><td>1</td><td>2</td></tr>
<tr><td>02</td><td>Family planning report</td><td>1</td><td>2</td><td>1</td><td>2</td><td>3</td><td>4</td><td>1</td><td>2</td></tr>
<tr><td>03</td><td>Laboratory report</td><td>1</td><td>2</td><td>1</td><td>2</td><td>3</td><td>4</td><td>1</td><td>2</td></tr>
<tr><td>04</td><td>Morbidity/ mortality report</td><td>1</td><td>2</td><td>1</td><td>2</td><td>3</td><td>4</td><td>1</td><td>2</td></tr>
<tr><td>05</td><td>Equipment status report</td><td>1</td><td>2</td><td>1</td><td>2</td><td>3</td><td>4</td><td>1</td><td>2</td></tr>
<tr><td rowspan="2">432</td><td rowspan="2" colspan="3">Does this facility have a designated person, such as a data manager, who is responsible for health services data in this facility?</td><td colspan="4">Yes</td><td>1</td><td colspan="2"></td></tr>
<tr><td colspan="4">No</td><td>2</td><td colspan="2">→434</td></tr>
<tr><td rowspan="3">433</td><td rowspan="3" colspan="3">Who is responsible for health services data in this facility?

PROBE TO DETERMINE WHO THIS PERSON IS</td><td colspan="4">Data manager/HMIS person/statistic</td><td>1</td><td colspan="2"></td></tr>
<tr><td colspan="4">Facility in-charge</td><td>2</td><td colspan="2"></td></tr>
<tr><td colspan="4">Other service provider (specify)_________________</td><td>96</td><td colspan="2"></td></tr>
<tr><td rowspan="2">434</td><td rowspan="2" colspan="3">Is the data entered on real time?</td><td colspan="4">Yes</td><td>1</td><td colspan="2"></td></tr>
<tr><td colspan="4">No</td><td>2</td><td colspan="2"></td></tr>
<tr><td rowspan="7">435</td><td rowspan="7" colspan="3">What is the frequency of reporting to the central system?</td><td colspan="4">Fortnightly</td><td>1</td><td colspan="2"></td></tr>
<tr><td colspan="4">Monthly</td><td>2</td><td colspan="2"></td></tr>
<tr><td colspan="4">Bi-monthly</td><td>3</td><td colspan="2"></td></tr>
<tr><td colspan="4">Quarterly</td><td>4</td><td colspan="2"></td></tr>
<tr><td colspan="4">Bi- annually</td><td>5</td><td colspan="2"></td></tr>
<tr><td colspan="4">Annually</td><td>6</td><td colspan="2"></td></tr>
<tr><td colspan="4">Others (specify) ______________________</td><td>96</td><td colspan="2"></td></tr>
</table>

Module 3: Service specific readiness
Section 5: Antenatal care

500	*CHECK Q.100.01:* ANC services available in facility available in facility	Yes		1	
		No		2	→next section
501	Number of days in a month antenatal care services offered at this facility? *USE A 4-WEEK MONTH TO CALCULATE # OF DAYS*	☐☐ No. of days/ month			
502	Do ANC providers provide any of the following services to pregnant women as part of routine ANC?	**Yes**	**No**		
01	Iron supplementation	1	2		
02	Folic acid supplementation	1	2		
03	Combination iron+ folate (IFA) supplementation	1	2		
04	Intermittent preventive treatment (IPT) for malaria	1	2		
05	Tetanus toxoid vaccination	1	2		
06	Provide misoprostol tablet/capsule for home-based deliveries	1	2		
07	Provide vitamin a within 42 days of delivery	1	2		
	Check Q502.05: if no→ go to Q506				
503	Is tetanus toxoid vaccination available on all days that ANC services are available in this facility?	Yes		1	
		No		2	
504	How many days each week are tetanus toxoid vaccinations available at this facility?	Days per week		☐	
		Less often than once/week		2	
505	Do ANC providers in this facility provide any of the following **tests** from this site to pregnant women as part of ANC?	**Always Available & functioning**	**Available but not functioning always**	**Not always available**	**Never available**
01	Urine protein test	1	2	3	4
02	Urine glucose test	1	2	3	4
03	Any rapid test for haemoglobin	1	2	3	4
04	Syphilis rapid diagnostic test	1	2	3	4
05	Blood grouping & typing	1	2	3	4
06	Ultrasonogram	1	2	3	4
506	Do ANC providers in this facility routinely diagnose and treat RTIs / STIs, or are RTI/STI clients referred to another provider or location for diagnosis and treatment?	Routinely diagnose and treat STIs		1	
		Diagnose but refer elsewhere for treatment		2	
		Refer elsewhere in facility for diagnosis & treatment		3	
		Refer outside facility for diagnosis & treatment		4	
		No diagnosis / treatment / referral		5	
507	Availability of national ANC guideline	Yes		1	→509

		No	2	
508	Availability of any other ANC guideline	Yes	1	
		No	2	
509	Availability of visual aids for client education on subjects related to pregnancy or antenatal care	Yes	1	
		No	2	
510	Are individual client cards or records for ANC maintained at this service site?	Yes	1	
		No	2	
511	Are individual client cards or records for PNC maintained at this service site?	Yes	1	
		No	2	

Equipment and supplies for routine ANC:

520	Describe the setting of the ANC service room or area.	Private room	1	
		Other room with auditory and visual privacy	2	
		Visual privacy only	3	
		No privacy	4	

521	If the following items are available in this service area and are functioning-	**Available and functioning**	**Available but not functioning**	**Not available**
01	Manual BP apparatus	1	2	3
02	Stethoscope	1	2	3
03	examination light	1	2	3
04	Fetal stethoscope	1	2	3
05	Adult weighing scale	1	2	3
06	Adult height scale	1	2	3
07	Examination bed or couch	1	2	3
08	Tape measure for fundal height	1	2	3
522	**Standard precautions and conditions for client examination**	**Always available**	**Not always available**	**Never available**
01	Running water (piped, bucket with tap or pour pitcher)	1	2	3
02	Hand-washing soap (may be liquid soap)	1	2	3
03	Alcohol-based hand rub	1	2	3
04	Waste receptacle (pedal bin) with lid and plastic bin liner	1	2	3
05	Sharps container ("safety box")	1	2	3
06	Disposable latex gloves	1	2	3
07	Disinfectant/antiseptics [e.g., chlorine, hibitane, alcohol]	1	2	3
08	Single-use standard disposable syringes with needles or 1 2 3 auto-disable syringes with needles	1	2	3
09	Medical masks	1	2	3
10	Gowns	1	2	3
11	Guidelines for standard precautions	1	2	3
12	Eye protection [goggles or face protection]	1	2	3

Section 6: Delivery and newborn care

600	*CHECK Q.100.02:*<br><br>Normal delivery available in facility	Yes	1	
		No	2	→next section
601	Is a person skilled in conducting deliveries present at the facility today or on call at all times (24 hours a day), including weekends, to provide care?<br><br>(Medical specialists, medical officers, nurses, and family welfare assistants)	Yes	1	
		No	2	→603
602	Is there a duty schedule or call list for 24-hr staff assignment?	Yes	1	
		No	2	

603	Please tell me if any of the following (B) PROVIDED IN PAST 6 MONTHS interventions have ever been carried out by providers as part of their work in this facility, and if so, whether the intervention has been carried out at least once during the past 3 months.	(a) Ever provided in facility (if yes→ (b); if no/ don't know → next question.			(b) Provided in past 6 months		
		Yes	No	Don't know	Yes	No	Don't know
01	Parenteral administration of antibiotics (IV or IM)	1	2	3	1	2	3
02	Parenteral administration of oxytocic (IV or IM)	1	2	3	1	2	3
03	Parenteral administration of anticonvulsant for hypertensive disorders of pregnancy (IV or IM)	1	2	3	1	2	3
04	Assisted vaginal delivery	1	2	3	1	2	3
05	Manual removal of placenta	1	2	3	1	2	3
06	Removal of retained products of conception	1	2	3	1	2	3
07	Neonatal resuscitation	1	2	3	1	2	3
08	Corticosteroids for pre-term labour	1	2	3	1	2	3

604	Do you have the national guidelines for BEmONC available in this service site?	Yes	1	
		No	2	
605	Do you have the national guidelines for CEmOC?<br><br>ACCEPTABLE IF PART OF ANOTHER GUIDELINE.	Yes	1	
		No	2	
606	Do you have guidelines or protocols on management of pre-term labour?<br><br>ACCEPTABLE IF PART OF ANOTHER GUIDELINE.	Yes	1	
		No	2	
607	Does this facility practice Kangaroo Mother Care for low birth weight babies?	Yes	1	
		No	2	→609
608	Is there a separate room or space for Kangaroo	Yes, separate room	1	

	Mother Care or is it integrated into the main postnatal ward?	Yes, integrated	2	
609	Do providers of delivery services in this facility use partograph to monitor labour and delivery?	Yes	1	
		No	2	→611
610	Are partographs used routinely (for all cases) or selectively (only for some cases) to monitor labour and delivery in this facility?	Yes, routinely	1	
		Yes, selectively	2	
611	Number of dedicated maternity beds are available in this facility	☐☐☐ beds		
612	Number of dedicated delivery beds are available in this facility	☐☐☐ beds		
613	Does the facility conduct regular reviews of maternal or newborn deaths or "near-misses"?	Yes	1	
		No	2	→ 627
614	Are reviews done for mothers only, newborns only, or for both mothers and newborns?	For mothers only	1	
		For newborns only	2	→616
		For both mothers and newborns	3	
615	How often are reviews of maternal deaths or "near misses" carried out?	Every ☐☐ weeks	1	
		Only when case occurs	2	
		Don't know	98	
	CHECK Q614: if "3" circled then ask next question, otherwise skip next question.			
616	How often are reviews of newborn deaths or "near misses" carried out?	Every ☐☐ weeks	1	
		Only when case occurs	2	
		Don't know	98	

Equipment and supplies for routine deliveries:

	ASK TO BE SHOWN THE LOCATION IN THE FACILITY WHERE NORMAL DELIVERY SERVICES ARE PROVIDED			
617	Describe the setting of the ANC service room or area.	Private room	1	
		Other room with auditory and visual privacy	2	
		Visual privacy only	3	
		No privacy	4	

618	If the following items are available in the delivery area and are functioning	**Available and functioning**	**Available but not functioning**	**Not available**
01	Incubator	1	2	3
02	Other external heat source	1	2	3
03	Examination light	1	2	3
04	Suction apparatus with catheter	1	2	3
05	Suction bulb or penguin sucker	1	2	3
06	Manual vacuum extractor	1	2	3
07	Vacuum aspiration lit or D&C kit	1	2	3
08	Newborn bag and mask (AMBU bag and mask)	1	2	3
09	Thermometer	1	2	3

10	Thermometer for low body temperature	1	2	3
11	Infant scale	1	2	3
12	Fetal stethoscope	1	2	3
13	Blood pressure machine	1	2	3
14	Stethoscope	1	2	3
15	Delivery bed complete with rods and stirrups	1	2	3
16	Delivery pack	1	2	3
17	Cord clamp	1	2	3
18	Speculum	1	2	3
19	Episiotomy scissors	1	2	3
20	Scissors or blade to cut cord	1	2	3
21	Suture material with needle	1	2	3
22	Needle holder	1	2	3
23	Forceps (large)	1	2	3
24	Forceps (medium)	1	2	3
25	Sponge holder	1	2	3
26	Blank partograph	1	2	3
27	Stairs (for climbing onto delivery bed)	1	2	3
619	If any of the following medicines or items are available at this service site on observation day			
01	Tetracycline eye ointment for newborn	1	2	3
02	Injectable antibiotic (e.g., ceftriaxone)	1	2	3
03	Injectable uterotonic (e.g., oxytocin)	1	2	3
04	Magnesium sulphate	1	2	3
05	Injectable diazepam	1	2	3
06	IV solution (ringer lactate) with infusion set	1	2	3
07	Skin disinfectant (other than chlorhexidine)	1	2	3
08	7.1% chlorhexidine solution (umbilical cord cleansing)	1	2	3
09	Hydralazine injection	1	2	3
10	Gentamycin eye ointment	1	2	3
620	**Standard precautions and conditions for client examination**	**Always available**	**Not always available**	**Never available**
01	Running water (piped, bucket with tap or pour pitcher)	1	2	3
02	Hand-washing soap (may be liquid soap)	1	2	3
03	Alcohol-based hand rub	1	2	3
04	Waste receptacle (pedal bin) with lid and plastic bin liner	1	2	3
05	Sharps container ("safety box")	1	2	3

06	Disposable latex gloves	1	2	3
07	Disinfectant/antiseptics [e.g., chlorine, hibitane, alcohol]	1	2	3
08	Single-use standard disposable syringes with needles or 1 2 3 auto-disable syringes with needles	1	2	3
09	Medical masks	1	2	3
10	Gowns	1	2	3
11	Guidelines for standard precautions	1	2	3
12	Eye protection [goggles or face protection]	1	2	3

Immediate newborn practice:

621	Ask the responsible or knowledgeable person of the facility / RMO/UHFPO/ Manager whether the facility performs following post-partum or newborn related practices:			
		**Yes**	**No**	**Don't know**
01	Delivery to the abdomen (Skin to Skin)	1	2	3
02	Drying and wrapping newborns to keep them warm	1	2	3
03	Initiation of breastfeeding within the first hour	1	2	3
04	Routine, complete (head-to-toe) examination of newborn 1 2 8<br>before discharge	1	2	3
05	Suction of the newborn by means of catheter	1	2	3
06	Suction of the newborn by means of suction bulb or penguin sucker	1	2	3
07	Weigh the newborn immediately	1	2	3
08	Administer Vitamin K to newborn	1	2	3
09	Apply Tetracycline or Gentamycin eye ointment to both eyes	1	2	3
10	Give full bath (immerse newborn in water) shortly (i.e., within a few minutes/hours) after birth	1	2	3
11	Give the newborn prelacteal liquids	1	2	3
12	Give the newborn OPV (oral polio vaccine/ polio zero vaccine) prior to discharge	1	2	3
13	Give the newborn BCG prior to discharge	1	2	3
14	Provide maternal Vitamin A	1	2	3

Section 7: Caesarean delivery

700	*CHECK Q.100.03:*<br><br>Normal delivery available in facility	Yes	1	
		No	2	→next section
701	Does the facility have a health worker who can perform Caesarean delivery (section) present at the facility or on call 24 hours a day (including weekends and on public holidays)?	Yes	1	
		No	2	→703
702	Is there a duty schedule or call list for 24-hr staff assignment?	Yes	1	
		No	2	
703	Does this facility have an anaesthetist present in the facility or on all 24 hours a day (including weekends and on public holidays)?	Yes	1	
		No	2	→705
704	Is there a duty schedule or call list?	Yes	1	
		No	2	
705	Have Caesarean deliveries been performed in this facility during the past 6 months?	Yes	1	
		No	2	
	ASK TO SEE THE ROOM OR AREA WHERE CESAREAN DELIVERIES ARE DONE AND ASK TO SEE THE ITEMS BELOW			

706	If the following equipment are available and functioning at this site on observation day	**Available and functioning**	**Available but not functioning**	**Not available**
01	Anaesthesia machine	1	2	3
02	Tubings and connectors (to connect endotracheal tube)	1	2	3
03	Oropharyngeal airway (adult)	1	2	3
04	Oropharyngeal airway (paediatric)	1	2	3
05	Endotracheal tube cuffed sizes 3.0 - 5.0	1	2	3
06	Endotracheal tube cuffed sizes 5.5 - 9.0	1	2	3
07	Intubating stylet	1	2	3
08	Spinal needle	1	2	3
09	OR table	1	2	3
10	OR light	1	2	3
11	IV stand	1	2	3
12	Emergency power supply	1	2	3
13	Instrument set for caesarean delivery	1	2	3
14	Air conditioner	1	2	3
15	Oxygen cylinder	1	2	3
16	Sterile gloves	1	2	3
17	Disinfectant	1	2	3
18	Autoclave area	1	2	3
19	Autoclave machine	1	2	3

Section 8: Family planning services

800	*CHECK Q.100.06:*<br><br>Normal delivery available in facility	Yes		1	
		No		2	→next section
	ASK TO BE SHOWN THE LOCATION IN THE FACILITY WHERE FAMILY PLANING SERVICES ARE PROVIDED				
801	Describe the setting of the family planning service room or area.	Private room		1	
		Other room with auditory and visual privacy		2	
		Visual privacy only		3	
		No privacy		4	
802	How many days in a month are family planning services offered at this facility?	**Provide (stock the commodity)**	**Prescribe/ counsel, or refer**	**No**	
803	Does this facility ***provide*** (i.e., stock the commodity) or ***prescribe, counsel or refer clients for*** any of the following modern methods of family planning:	1	2	3	
01	Combined oral contraceptive pills	1	2	3	
02	Progestin-only contraceptive pills	1	2	3	
03	Combined injectable contraceptives	1	2	3	
04	Progestin-only injectable contraceptives (depo)	1	2	3	
05	Male condoms	1	2	3	
06	Intrauterine contraceptive device (iucd)	1	2	3	
07	Implant	1	2	3	
08	Emergency contraceptive pills (e.g., prostinol 2)	1	2	3	
09	Counsel clients on periodic abstinence	1	2	3	
10	Vasectomy (male sterilization)	1	2	3	
11	Tubal ligation (female sterilization)	1	2	3	
12	Other methods (e.g., spermicide or diagphragm)	1	2	3	
13	Counsel clients on lactation amenorrhea (lam)	1	2	3	
804	Availability of **national family planning guidelines** available at this facility	Yes		1	
		No		2	
805	Are individual records or cards (e.g., IUCD card, IMPLANT card, etc.,) maintained at this service site for family planning clients?	Yes		1	
		No		2	
806	Does this facility have a system whereby certain observations and parameters are routinely carried out on family planning clients before the consultation takes place?	Yes		1	
		No		2	
807	If yes, ask whether following activities are done:	**Activity routinely done**	**Activity not routinely done**	**Don't know**	

01	Weighing of clients	1	2	3
02	Taking blood pressure	1	2	3
03	Conducting group health education sessions	1	2	3

808	Do family planning providers in this facility routinely diagnose and treat RTIs/STIs, or are RTIs/STIs clients referred to another provider or location for STI diagnosis and treatment?	Routinely diagnose and treat STIs	1	
		Diagnose but refer elsewhere for treatment	2	
		Refer elsewhere in facility for diagnosis and treatment	3	
		Refer outside facility for diagnosis and treatment	4	
		No diagnosis/ treatment referral	5	

End time of observation:☐☐: ☐☐ (24 hour)

Tool 2: Interview guideline for KII of Health Facility Manager

Objective(s) of the interview:

1. Understand the availability and utilization of services
2. Understand the coverage of service from service provider's view point
3. Understand challenges facing to provide adequate & quality service (ANC, PNC, EmOC, C-section, OPD)
4. Understanding bottlenecks of different management issues (MIS, HR, supply)
5. Understand the level of awareness about health policies
6. Suggestion to improve services.

Guiding questions

1. Which health care services are provided from this facility? (ANC, PNC, EmOC, Normal delivery, C-section, OPD)
2. Are these services provided regularly? (If not, why, where is the problem)
3. Can please say about structural facilities of this health center?
    - Adequate numbers of room
    - Separate room for each service ((ANC, PNC, EmOC, Normal delivery, C-section, OPD)
    - Waiting area for patients & their attendants
    - Number of beds
    - Availability of night guard
    - If 24/7 then generator is available or not
    - Availability of fresh water. If not available then how managed?
4. How many patients seek care per month from this facility? (Day→Week→Month)
5. Do you any idea about the highest distance from where patients come to seek care?
6. Those who come from a very far area what types of difficulties they have to face to reach the facility?
7. What hinders providing proper service? (HR, Medicine)
8. Can you please tell about staffs working at this facility? (Number & position)
9. How frequently staffs transfer from this facility? Does it create any problem?
10. Do you think HR is enough? (If not, why)
11. Are there enough field level staff working in the hard to reach areas?
    - Necessary number of HA/FWA in respective areas
    - How managed where staff in not available?
12. Is there enough staff available for operating delivery? (if not, how managed)
13. If referral needed during delivery, do you have the arrangement? (What; if not how managed)
14. Is ambulance accessible to all the corner of this area?
15. If not, how people of that area reach hospital in case of emergency?
16. How many reports you have to send from here and to whom/which office?

17. Is there any monitoring system to monitor staff's daily activity?
    - If yes, what?
    - If not, do you think it's necessary?
    - Which areas should be focused in that case?
18. Is there any problem arise to send report preparation and sending?
19. Is there any arrangement of Local Level Planning and solving problem locally?
    - If yes, is there any influence of local government in this planning?
    - Does these planning influences the quality of service? (how)
20. What kinds of services are available for babies at this facility?
    - KMC, IMCI corner, Sepsis management
    - What kind of challenges you have to face to providing these services?
21. Is vaccination service is available at this facility?
    - How many babies take vaccine from this facility?
    - Is there any arrangement of providing vaccine in camp mode?
    - Is there any problem to arrange camp? (What)
22. Is there any policy or guideline of HtR areas healthcare system?
    - If yes, can please tell something about that?
    - If no, do you think there should be a policy? (Why)
    - What should be addressed in that policy or guideline?

THANK YOU for taking the time to participate in this interview.

Objective(s) of the interview:

7. Understand the availability and utilization of services
8. Understand the coverage of service from service provider's view point
9. Understand challenges facing to provide adequate & quality service (ANC, PNC, EmOC, C-section, OPD)
10. Understanding bottlenecks of different management issues (MIS, HR, supply)
11. Understand the level of awareness about health policies
12. Suggestion to improve services.

Guiding questions

২. আপনার স্বাস্থ্যকেন্দ্র থেকে সাধারনত কি কি সেবা প্রদান করা হয়? (ANC, PNC, EmOC, Normal delivery, C-section, OPD)

৩. এই সেবাগুলো কি নিয়মিত দেয়া হয়? (না হলে, কেন হয় না, সমস্যা কোথায়)

৪. সেবা কেন্দ্রের কাঠামোগত সুবিধা সম্পর্কে কিছু বলুন।

- যথেষ্ট কক্ষ আছে কি না?
- প্রতিটি সেবা প্রদানের জন্য আলাদা কক্ষ বরাদ্দ আছে কি না? (ANC, PNC, EmOC, Normal delivery, C-section, OPD)
- রোগীদের বসার জন্য অপেক্ষা করা জায়গা আছে কি না?
- কতগুলো বেড আছে?
- নাইট গার্ড আছে কি না?
- ২৪/৭ সেবা কেন্দ্র হলে রাতে জেনারেটর এর ব্যবস্থা আছে কি না?
- সুপেয় পানির ব্যবস্থা আছে কি? না থাকলে কিভাবে কাজ করা হয়?

৫. প্রতি মাসে আনুমানিক কি পরিমান রোগী এই স্বাস্থ্য কেন্দ্র থেকে সেবা গ্রহণ করেন? (দিন--->সপ্তাহ--->মাস)

৬. আপনার কি কোন ধারনা আছে যে সর্বোচ্চ কতদূর থেকে মানুষ এখানে সেবা নিতে আসে?

৭. যেসব রোগী অনেক দূর থেকে আসেন তাদের এখানে পৌছাতে কি ধরনের সমস্যার সম্মখীন হতে হয়?

৮. সেবা প্রদান করার ক্ষেত্রে আপনি কি ধরনের সমস্যার সম্মুখীন হনদ? (জনবল,ঔষধ, অন্যান্য)

৯. আপনার এই সেবা কেন্দ্রে মোট কতজন এবং কি কি পদবীর কর্মকর্তা কর্মরত আছেন?

১০. আপনার এখানে বদলি হওয়ার হার/প্রবনতা কেমন? এর ফলে কি কোন ধরনের সমস্যা হয়?

১১. এই পরিমান জনবল কি যথেষ্ট বলে মনে করেন? (না হলে, কেন/কোথায় সমস্যা হয়)

১২. দুর্গম এলাকায় কি প্রয়োজনীয় সংখ্যক সরকারি মাঠকর্মী কর্মরত আছেন?

- প্রতিটি ওর্য়াডে কি প্রয়োজনীয় সংখ্যক স্বাস্থ্য সহকারি/পরিবার পরিকল্পনা সহকারী আছেন?
- যেসব জায়গায় নাই সেসব জায়গায় কিভাবে কাজ চলে?

১৩. ডেলিভারি করানোর জন্য কি প্রয়োজনীয় সংখ্যক জনবল আছে? (না থাকলে কিভাবে ম্যানেজ হয়)

১৪. ডেলিভারির সময় যদি রেফার করার দরকার হয় তাহলে কি তার জন্য প্রয়োজনীয় ব্যবস্থা আছে? (কি ব্যবস্থা, না থাকলে কিভাবে ম্যানেজ করা হয়)

১৫. এই এলাকায় সব জায়গায় কি এ্যম্বুলেন্স যা্ওয়ার মতো রাস্তা আছে?

১৬. যেসব জায়গায় এ্যম্বুলেন্স পৌছানো সম্ভব না তারা কিভাবে স্বাস্থ্য কেন্দ্রে পৌছান?

১৭. আপনার এখান থেকে কোথায় এবং কি কি ধরনের রিপোর্ট দিতে হয়?

১৮. কর্মকর্তা/কর্মচারীদের প্রাত্যহিক কাজ মনিটরিং এর কি কোন ব্যবস্থা আছে?

- থাকলে কি?
- না থাকলে, আপনি কি মনে করেন এই ধরনের ব্যবস্তা থাকা প্রয়োজন?
- এই ধরনের মনিটরিং এর ক্ষেত্রে কি কি বিষয় গুরুত্ব দেয়া উচিৎ?

১৯. রিপোর্ট তৈরি করা এবং পাঠানোর ক্ষেত্রে কি কোন ধরনের জটিলতা/সমস্যা হয়? (হলে কি ধরনের)

২০. আপনাদের এখানে কি স্থানীয় পর্যায়ে পরিকল্পনা প্রনয়ন করে স্থানীয়ভাবে সমস্যা সমাধানের কোন উদ্যোগ নেয়া হয়?

- (হ্যাঁ হলে) এই পরিকল্পনায় কি স্থানীয় সরকারের কোন ভুমিকা আছে? (হ্যাঁ হলে, কি ধরনের)
- এই পরিল্পনা দ্বারা কি সেবার মান কোনভাবে প্রভাবিত হয়েছে? (কিভাবে, কি ধরনের)

২১. এই স্বাস্থ্যকেন্দ্রে শিশু স্বাস্থ্য এর জন্য কি কি সেবা চালু আছে?

- KMC, IMCI কর্ণার, সেপসিস ম্যানেজমেন্ট
- এই সেবাগুলো প্রদানের ক্ষেত্রে কি ধরনের সমস্যার সম্মুখীন হতে হয়?

২২. এই স্বাস্থ্য কেন্দ্রে কি শিশুদের টিকা দেয়ার ব্যবস্থা আছে?

- কি পরিমান বাচ্চা প্রতি মাসে টিকা গ্রহন করে?
- ক্যাম্প এর মাধ্যমে কি টিকা দেয়া হয়?
- সেক্ষেত্রে কি কোন ধরনের সমস্যা হয়? (হ্যাঁ হলে কি)

২৩. আপনার জানামতে দুর্গম এলাকায় স্বাস্থ্যসেবা প্রদানের জন্য কি কোন ধরনের বিশেষ পলিসি বা গাইডলাইন আছে?

- (হ্যাঁ হলে) উক্ত গাইড লাইনে কি ধরনের নির্দেশনা আছে সে সম্পর্কে কি একটু বলবেন?
- না হলে, আপনার কি মনে হয় এ সংক্রান্ত বিশেষ নির্দেশনামূলক কিছু থাকা উচিৎ?
- যদি নতুন গাইড লাইন তৈরি করা হয় তাহলে সেখানে কোন বিষয়গুলো প্রাধান্য দেওয়া উচিৎ বলে আপনি মনে করেন?

(গবেষনায় অংশগ্রহন করা এবং সময় দেয়ার জন্য আপনাকে ধন্যবাদ)

Tool 4: Household survey questionnaire

সম্প্রতি প্রসবকারী মহিলাদের জন্য প্রশ্নপত্র Questionnaire for Recently Delivered Women (RDW)

Information about interviwere:

সাক্ষাতকারগ্রহনকারীর নাম ও কোড: ______________________	□□□□	তারিখ: □□-□□-□□ দিন মাস বছর	
কারখানার নাম ও কোড Name and Code of workplace ________________________________			□□
সাক্ষাৎকার শুরুর সময় (২৪ ঘন্টা)	□□:□□ ঘন্টা : মিনিট	সাক্ষাৎকার শেষের সময় (২৪ ঘন্টা)	□□ :□□ ঘন্টা : মিনিট

Identification of the household:

	নাম Name	কোড Code
জেলা District		□
উপজেলা Sub-district		□□
ইউনিয়ন Union		□□
ক্লাস্টার নং Cluster number		□□□□
গ্রামের নাম ও কোড Name of village & code		□□□
বাড়ির নাম ও নং Name of the house & number		□□□□
খানা প্রধানের নাম ও খানা নং Name of household head & HH #		□□
সম্প্রতি প্রসবকারী মহিলার নাম ও নম্বর Name of RDW & number		□□
সম্প্রতি প্রসবকারী মহিলার স্বামীর নাম Name of husband of RDW		

(Supervision) তত্ত্বাবধায়ন	(Name) নাম	(Code) কোড	(Date) তারিখ
Reviewed by Supervisor	____________________	|___|___|	|___|___|-|___|___|-|___|___|
Checked by Field Editor	____________________	|___|___|	|___|___|-|___|___|-|___|___|
Reviewed by Office Editor	____________________	|___|___|	|___|___|-|___|___|-|___|___|
Keyed by	____________________	|___|___|	|___|___|-|___|___|-|___|___|

Section A: Household Section (খানার তথ্য)

এখন আমি আপনার এবং আপনার খানার সম্পর্কে কিছু তথ্য জানতে চাই। Now I would like to know some information about you and your household.

No.	Questions	Responses		Code	skip
11	আপনার খানায় সাধারণত কতজন লোক বাস করে? অর্থাৎ আপনাদের খানার মোট সদস্য সংখ্যা কতজন?<br>How many members usually live in your household? Please give the number of household member	খানার মোট সদস্য সংখ্যা<br>Total # of household member		☐☐	
12	আপনাদের খানায় কোন্ বয়সের কতজন পুরম্নষ এবং মহিলা আছে বলুন<br>Now tell me how many male and female members are there in your household:<br>কোন বয়সের পুরম্নষ এবং মহিলা সদস্য না থাকলে বক্সে '00' লিখুন।<br>If none, write '00' in box.<br>*(পুরম্নষ এবং মহিলার সংখ্যা যোগ করে মোট এর বক্সে লিখুন)*<br>Calculate the total number of male and female and write in the Total box		পুরম্নষ<br>Male	মহিলা<br>Female	
		0-4 বছর **Y**ears	☐☐	☐☐	
		5-14 বছর **Y**ears	☐☐	☐☐	
		15-29 বছর **Y**ears	☐☐	☐☐	
		30-49 বছর **Y**ears	☐☐	☐☐	
		50 বছর বা বেশী ≥50 **Y**ears	☐☐	☐☐	
		মোট Total	☐☐	☐☐	
13	<u>সাক্ষাৎকারগ্রহণকারীঃ</u> প্রশ্ন ১২ থেকে পুরম্নষ এবং মহিলার মোট সংখ্যা বক্সে লিখে প্রশ্ন ১১ এর সাথে মিলিয়ে দেখুন। অসামঞ্জস্য হলে প্রয়োজনে প্রশ্ন ১১ এবং/বা প্রশ্ন ১২ সংশোধন করম্নন।<br>Write the total of male and female members from Q12 in the box and reconcile with Q11	মোট পুরম্নষ এবং মহিলা<br>Total number of male and female		☐☐	
14	থালা বাসন ধোয়ার জন্য প্রধানতঃ আপনারা কোথাকার পানি ব্যবহার করেন?<br>What is the main source of water your household used for cleaning utensils?	<u>পাইপের পানি</u> Piped water<u>:</u>			
		বাড়ির ভিতরে ট্যাপের পানি Piped inside dwelling		11	
		বাড়ির বাহিরে ট্যাপের পানি Piped outside dwelling		12	
		<u>কূপের পানি</u> Well water<u>:</u>			
		নলকূপ Tubewell		21	
		অগভীর নলকূপ Shallow Tubewell		22	
		গভীর নলকূপ Deep Tubewell		23	
		কূয়া Surface Well/Other Well		24	
		<u>ভূ-পৃষ্ঠের পানি</u> Surface water<u>:</u>			
		পুকুর/বদ্ধ জলাশয়/হ্রদ Pond/Tank/Lake		31	
		নদী/খাল/ঝর্ণার পানি River/Stream		32	
		বৃষ্টির পানি Rain water		41	
		অন্যান্য Other___________________________		96	
		(নির্দিষ্ট করম্নন) Specify			
15	আপনার খানার সদস্যরা সাধারনত কি ধরণের পায়খানা/ল্যাট্রিন ব্যবহার করে?<br>What kind of toilet facility do members of your household usually use?	<u>ফ্লাশিং টয়লেট</u> Flush Toilet			
		ফ্লাশ করে সেপটিক ট্যাংকেপাঠানো / আধুনিক ল্যাট্রিন<br>Flush to Septic Tank/Modern Toilet		11	
		<u>গর্ত (পিট) টয়লেট/ল্যাট্রিন</u> Pit Toilet/Latrine<u>:</u>			
		জলাবদ্ধ/স্ল্যাব (স্যানিটারী) ল্যাট্রিন Water sealed/Slab Latrine		21	

No.	Questions	Responses				Code	skip
		জলাবদ্ধ নয়, গর্তের (পিট) ল্যাট্রিন Pit Latrine				22	
		খোলা/ঝুলন্ত ল্যাট্রিন Open/Hanging Latrine				23	
		ল্যাট্রিন নাই/ঝোপ-ঝাড়/মাঠ No Facility/Bush/Field				31	
		অন্যান্য Other______________________________ নির্দিষ্ট করুন (Specify)				96	
16	আপনার খানায় কি নিম্নের জিনিষগুলো ব্যবহার উপযোগী অবস্থায় আছে?<br>Does your household have following usuable goods?<br><br>বিদ্যুৎ/সৌর বিদ্যুৎ Electricity/Solar electricity?<br>রেডিও A radio?<br>টেলিভিশন A television<br>মোবাইল ফোন A mobile phone?<br>মোবাইল ছাড়া অন্য টেলিফোন A non-mobile phone?<br>ফ্রিজ A refrigerator<br>আলমারী/ওয়ার্ডরোব An almirah/wardrobe?<br>টেবিল A table?<br>চেয়ার/বেঞ্চ A chair?<br>বৈদ্যতিক পাখা An electric fan?<br>ডিভিডি/ভিসিডি প্লেয়ার A DVD/VCD player?<br>পানির পাম্প A water pump?<br>রিকসা/ভ্যান Rickshaw/Van<br>বাইসাইকেল Bicycle<br>মোটরসাইকেল Motorcycle<br>সি,এন,জি/টেম্পু/ইলেকট্রিক বাইক CNG/Tempo/Electric bike<br>নৌকা Boat<br>আই, পি, এস/জেনারেটর IPS/Generator<br>কমপিউটার/ল্যাপটপ Computer/Laptop<br><br>প্রত্যেকটি জিনিস সম্বন্ধে জিজ্ঞেস করুন<br>*Ask for every item*		**ITEM**	YES	NO	**#**	
		A	বিদ্যুৎ/সৌর বিদ্যুৎ Electricity/Solar electricity	1	2		
		B	রেডিও radio	1	2		
		C	টেলিভিশন television	1	2		
		D	মোবাইল ফোন mobile phone	1	2		
		E	অন্য টেলিফোন non-mobile phone	1	2		
		F	ফ্রিজ refrigerator	1	2		
		G	আলমারী/ওয়ার্ডরোব almirah/wardrobe	1	2		
		H	টেবিল table	1	2		
		I	চেয়ার/বেঞ্চ chair/bench	1	2		
		J	বৈদ্যতিক পাখা electric fan	1	2		
		K	ডিভিডি/ভিসিডি প্লেয়ার DVD/VCD player	1	2		
		L	পানির পাম্প water pump	1	2		
		M	রিকসা/ভ্যান Rickshaw/Van	1	2		
		N	বাইসাইকেল Bicycle	1	2		
		O	মোটরসাইকেল Motorcycle	1	2		
		P	সি,এন,জি/টেম্পু/ইলেকট্রিক বাইক CNG/Tempo/Electric bike	1	2		
		Q	নৌকা Boat	1	2		
		R	আই, পি, এস/জেনারেটর IPS/Generator	1	2		
		S	কমপিউটার/ল্যাপটপ Computer/Laptop	1	2		
17	এই খানার নিজস্ব কতগুলি নিম্নে বর্ণিত পশু-পাখি আছে?<br>How many of the following animals does this household own?		পশুপাখি Animal			সংখ্যা Number	
		A	মহিষ Buffaloes			☐☐	

No.	Questions		Responses	Code	skip
	জানা না থাকলে **‘97’** লিখুন If unknown, enter ‘97’<br>কোনটি না থাকলে **‘00’** লিখুন If none enter ‘00’<br>৯৫ টি বা তার বেশী থাকলে **‘95’** লিখুন If 95 or more, enter ‘95’<br><br>মহিষ Buffaloes?<br>ষাঁড়/গরু Bulls/ Milk cows?<br>ছাগল/ভেড়া Goat / sheep?<br>মুরগি/হাঁস Chicken / Duck?	B	ষাঁড় / গরু Bulls / Milk cows	☐☐	
		C	ছাগল / ভেড়া Goat / shee	☐☐	
		D	মুরগি / হাঁস Chicken / Duck	☐☐	
18	বসত ঘরের চালের/ছাদের প্রধান নির্মাণ-সামগ্রীঃ<br>(দেখে লিপিবদ্ধ করুন)<br><br>Main Material of the **Roof:**<br>(Record observation.)		কাঁচা ছাদ Natural Roof:		
			খর/পাতা (Thatch / leaf))	11	
			প্রাথমিক পর্যায়ের ছাদ Rudimentary Roof:		
			বাঁশ Bamboo	21	
			কাঠের তক্তা Wood planks	22	
			পরিপূর্ণ ছাদ Finished roof:		
			টিন Tin	31	
			পলিস করা কাঠ Polished Wood	32	
			সিরামিক টাইলস Ceramic tiles	33	
			সিমেন্ট, কনক্রিট Cement/Concrete	34	
			অন্যান্য Other	96	
			নির্দিষ্ট করুন (Specify)		
19	বসত ঘরের দেয়ালের প্রধান নির্মাণ-সামগ্রীঃ<br>(দেখে লিপিবদ্ধ করুন)<br>Main material of the **Walls**:<br>(Record observation.)		কাঁচা দেয়াল Natural Walls:		
			কোন দেয়াল নেই No walls	11	
			ছরি / পাতা / গাছের কান্ড Cane/Palm/Trunks	12	
			প্রাথমিক পর্যায়ের দেয়াল Rudimentary Walls:		
			মাটি Earth/clay/mud	21	
			বাঁশ Bamboo	22	
			পাথর Stone	23	
			কাঠের তক্তা Wood planks	24	
			পরিপূর্ণ দেয়াল Finished Walls:		
			টিন Tin	31	
			সিমেন্ট Cement	32	
			ইট Brick	33	
			অন্যান্য Other___________________________	96	
			নির্দিষ্ট করুন (Specify)		
20	বসত ঘরের মেঝের প্রধান নির্মাণ-সামগ্রীঃ<br>(দেখে লিপিবদ্ধ করুন)		কাঁচা মেঝে Natural floor:		
			মাটি / বালু Earth / Sand	11	

No.	Questions	Responses	Code	skip
	Main material of the **Floor**:<br>(Record observation.)	প্রাথমিক পর্যায়ের মেঝে Rudimentary floor:		
		কাঠের তক্তা Wood planks	21	
		বাঁশ Bamboo	22	
		পরিপূর্ণ মেঝে Finished floor:		
		পলিস করা কাঠ Polished Wood	31	
		সিরামিক টাইলস Ceramic tiles	32	
		সিমেন্ট, কনক্রিট Cement/Concrete	33	
		অন্যান্য Other______________________________<br>নির্দিষ্ট করুন (Specify)	96	
21	আপনাদের খানার মালিকানায় বসত ভিটা আছে কি?<br>যদি না হয়, PROBE করুনঃ<br>আপনাদের খানার অন্য কোথাও বসত ভিটা আছে কি?<br>Does your household own any homestead?<br>IF 'NO', PROBE:<br>Does your household own homestead at any other places?	হ্যাঁ Yes	1	
		না No	2	
22	(খানার বসত ভিটা ছাড়া) আপনাদের কোন জমি আছে কি? Does your household own any land (other than the homestead land)?	হ্যাঁ Yes	1	
		না No	2	
23	রান্না করার জন্য আপনার খানায় সাধারনত কি ধরনের জ্বালানি ব্যবহার করা হয়?<br><br>What type of fuel does your household mainly use for cooking?	বিদ্যুৎ Electricity	01	
		এল,পি,জি গ্যাস LPG gas	02	
		প্রাকিতিক গ্যাস Natural gas	03	
		জৈব গ্যাস Bio gas	04	
		কেরোসিন তেল Kerosine oil	05	
		খনিজ কয়লা Coal, Lignite	06	
		অন্য কয়লা Charcoal	07	
		কাঠ Wood	08	
		খর/কুটা/শুকনো ঘাস Straw/Shrubs/Grass	09	
		কৃষিজাত শস্য Agricultural crop	10	
		গোবর Cow dung	11	
		খানায় রান্না করা হয় না No food cooked in household	95	
		অন্যান্য Other (specify)__________________	96	

Section B: RDW and her husband's background সম্প্রতি প্রসবকারী মহিলা এবং তার স্বামীর তথ্য

এখন আমি আপনার (সম্প্রতি প্রসবকারী মহিলা) এবং আপনার স্বামী সম্পর্কে কিছু প্রশ্ন জিজ্ঞেস করতে চাই।

Now I would like to ask some questions about you (recently delivered woman) and your husband

No.	Questions	Responses	Code
101	আপনি কোন্ সালের কোন্ মাসে জন্মগ্রহন করেছিলেন?	মাস Month .... ☐☐	

No.	Questions	Responses		Code
	In what month and year were you born?	জানি না Don't know month 97		
		সাল Year .... ☐☐☐☐		
		জানি না Don't know year.....................................9997		
102	বর্তমানে আপনার বয়স কত?<br>How old are you now?<br>(১০২ এবং ১০১ মিলিয়ে দেখুন, অসামঞ্জস্য হলে ১০২ এবং/বা ১০১ সংশোধন করুন)<br>(Compare and correct 101 and/or 102 if inconsistent)	বয়স (পূর্ণ বছরে) Age in completed years .... ☐☐		
103	আপনি কি কখনও স্কুলে বা মাদ্রাসায় লেখাপড়া করেছেন?<br>Have you ever attended school/madrasa?	হ্যাঁ স্কুল Yes School	1	
		হ্যাঁ মাদ্রাসা Yes Madrasa	2	
		হ্যাঁ উভয়ই Yes Both	3	
		না No	4	→106
104	আপনি সর্বশেষ কোন্ বিদ্যালয়/স্তর পর্যন্ত পড়াশুনা করেছেন?<br>What is the highest level of school you have last attended: primary, secondary, or higher?	প্রাইমারী/প্রাথমিক Primary	1	
		মাধ্যমিক Secondary	2	
		কলেজ/বিশ্ববিদ্যালয় College/university	3	
105	আপনি সর্বোচ্চ কোন্ ক্লাশ পাশ করেছেন?<br>What is the highest class that you have completed?	ক্লাশ Class .... ☐☐<br>(কোন ক্লাশ পাশ না করলে '00' লিখুন।)<br>If completed less than one year, record "00"		
106	আপনার ধর্ম কি?<br>What is your religion?	ইসলাম Islam	1	
		হিন্দু Hinduism	2	
		বৌদ্ধ Buddhism	3	
		খৃষ্টান Christianity	4	
		অন্যান্য নির্দিষ্ট করুন Other:______________________	6	
107	আপনি হয়ত জানেন যে কোন কোন মহিলা নগদ টাকা বা জিনিসপত্রের বিনিময়ে কাজ করে, কেউ জিনিসপত্র বিক্রি করে, কেউ নিজের ছোট ব্যবসায় বা পারিবারিক খামারে কাজ করে। বর্তমানে আপনি এ ধরনের কিছু বা অন্য কোন কাজ করছেন কি?<br>As you know, some women take up jobs for which they are paid in cash or kind. Others sell things, have a small business or work on the family farm or in the family business. Are you doing any of this things or any work?	হ্যাঁ Yes	1	
		না No	2	→110
108	প্রধানতঃ আপনি কি কাজ করেন?<br>What is your primary occupation, that is, what kind of work do you mainly do?	শারীরিক পরিশ্রম ভিত্তিক কাজঃ Physical work:		
		নিজের জমিতে চাষাবাদ বা বর্গাচাষী<br>Work on own farm or as a share cropper	01	
		দিন মজুর/অদক্ষ শ্রমিক (গৃহস্থালী,কৃষি ভিত্তিক ইত্যাদি)	02	

No.	Questions	Responses		Code
	নির্দিষ্টপেশা নীচে লিখুনঃ<br>Write down the occupation below:<br><br>____________________________________<br><br>____________________________________	Day/unskilled laborer (domestic, agricultural and igrant)		
		দক্ষ শ্রমিক (দীর্ঘ মেয়াদে চুক্তিবদ্ধ/ কাঠমিস্ত্রী/রাজমিস্ত্রী/জেলে<br>Skilled worker (long term contracted laborer)	03	
		রিক্সা চালক/ভ্যান চালক/নৌকা চালক<br>Rickshaw/Van puller/Boat driver	04	
		অ-শারীরিক পরিশ্রম ভিত্তিক কাজঃ Non physical work:		
		নিজস্ব ব্যবসা (দোকান/সেলাই/খামার/কুটির শিল্প) Own business	05	
		চাকুরীজীবি/পেশাজীবি (ডাক্তার, প্রকৌশলী, উকিল, শিক্ষক)<br>Service holder/Professionals	06	
		অন্যান্য নির্দিষ্ট করুন Other: ________________	96	
109	আপনি কি সারা বছর ধরে কাজ করেন? নাকি নির্দিষ্ট মৌসুমে কাজ করেন? নাকি মাঝে মধ্যে কাজ করেন?<br>Do you usually work throughout the year, or do you work seasonally, or only once in a while?	সারা বছর ধরে Throughout the year	1	
		নির্দিষ্ট মৌসুমে Seasonally/Part of the year	2	
		মাঝে মধ্যে Once in a while	3	
110	আপনি বর্তমানে বিবাহিতা, বিচ্ছিন্না, পরিত্যক্তা, বিধবা না তালাকপ্রাপ্তা নাকি কখনও বিয়ে হয়নি?<br>Are you currently married or separated or deserted or divorced or widowed, or never been married?	বর্তমানে বিবাহিতা Currently married	1	
		বিচ্ছিন্না Separated	2	→201
		পরিত্যক্তা Deserted	3	→201
		তালাকপ্রাপ্তা Divorced	4	→201
		বিধবা Widowed	5	→201
		কখনও বিয়ে হয়নি Never married	6	**→END**
111	আপনার স্বামী কোন্ সালের কোন্ মাসে জন্মগ্রহন করেছিলেন?<br>In what month and year were your husband born?	মাস Month .... [ ][ ]		
		জানি না Don't know month 97		
		সাল Year .... [ ][ ][ ][ ]		
		জানি না Don't know year 9997		
112	বর্তমানে আপনার স্বামীর বয়স কত?<br>How old is he now?<br>(১১১ এবং ১১২ মিলিয়ে দেখুন, অসামঞ্জস্য হলে ১১১ এবং/বা ১১২ সংশোধন করুন)<br>(Compare and correct 111 and/or 112 if inconsistent)	বয়স (পূর্ণ বছরে) Age in completed years .... [ ][ ]		
113	আপনার স্বামী কখনও স্কুলে বা মাদ্রাসায় লেখাপড়া করেছেন কি?<br>Has your husband ever attended school /madrasa?	হ্যাঁ স্কুল Yes School	1	
		হ্যাঁ মাদ্রাসা Yes Madrasa	2	
		হ্যাঁ উভয়ই Yes Both	3	
		না No	4	→116
114	আপনার স্বামী সর্বশেষ কোন্ বিদ্যালয়/স্তর পর্যন্ত পড়াশুনা করেছেন?	প্রাইমারী/প্রাথমিক Primary	1	

No.	Questions	Responses		Code
	What is the highest level of school your husband attended: primary, secondary, or higher?	মাধ্যমিক Secondary	2	
		কলেজ/বিশ্ববিদ্যালয় College/university	3	
115	আপনার স্বামী সর্বোচ্চ কোন্ ক্লাশ পাশ করেছেন? What is the highest class your husband completed?	ক্লাশ Class .... ☐☐ (কোন ক্লাশ পাশ না করলে '00' লিখুন।) If completed less than one year, record "00"		
116	বর্তমানে আপনার স্বামী আয় রোজগারের জন্য কোন কাজ করেন কি? Does your husband do anything for living?	হ্যাঁ Yes	1	
		না No	2	→201
117	আপনার স্বামীর প্রধান পেশা কি? একাধিক পেশার সাথে জড়িত হলে প্রধান পেশার নাম নিচে লিখে ডান দিকের কোড বৃত্তায়িত করুন। What is his primary occupation, that is, what kind of work does he mainly do? নির্দিষ্টপেশা নীচে লিখুনঃ Write down the occupation below: ________________________ ________________________	শারীরিক পরিশ্রম ভিত্তিক কাজঃ Physical work:		
		নিজের জমিতে চাষাবাদ বা বর্গাচাষী Work on own farm or as a share cropper	01	
		দিন মজুর/অদক্ষ শ্রমিক (গৃহস্থালী,কৃষি ভিত্তিক ইত্যাদি) Day/unskilled laborer (domestic, agricultural and migrant)	02	
		দক্ষ শ্রমিক (দীর্ঘ মেয়াদে চুক্তিবদ্ধ/ কাঠমিস্ত্রী/রাজমিস্ত্রী/জেলে Skilled worker (long term contracted laborer)	03	
		রিক্সা চালক/ভ্যান চালক/নৌকা চালক Rickshaw/Van puller/Boat driver	04	
		অ-শারীরিক পরিশ্রম ভিত্তিক কাজঃ Non physical_work:		
		নিজস্ব ব্যবসা (দোকান/সেলাই/খামার/কুটির শিল্প) Own business	05	
		চাকুরীজীবি/পেশাজীবি (ডাক্তার, প্রকৌশলী, উকিল, শিক্ষক) Service holder/Professionals	06	
		অন্যান্য নির্দিষ্ট করুন Other: __________________	96	
118	আপনার স্বামী কি সারা বছর ধরে কাজ করেন? নাকি নির্দিষ্ট মৌসুমে কাজ করেন? নাকি মাঝে মধ্যে কাজ করেন? Does your husband usually work throughout the year, or does he work seasonally, or only once in a while?	সারা বছর ধরে Throughout the year	1	
		নির্দিষ্ট মৌসুমে Seasonally/Part of the year	2	
		মাঝে মধ্যে Once in a while	3	

Section C: Information about nearest health facility নকিটবর্তী স্বাস্থ্যকন্দ্রে সম্পর্কে তথ্য

No.	Questions and filters	Responses	Code	Skip
201	আপনার বাড়ি থকেে নকিটবর্তী স্বাস্থ্যকন্দ্রে কতদূরে অবস্থতি? How far the nearest helath care center is from your home?	আধা কমিি এর মাঝে < 0.5 km	1	
		০.৫- ১.০ কমিি এর মাঝে within 0.5- 1.0 km	2	
		১.0 -৫.০ কমিরি মাঝে within 1.0- 5.0 km	3	
		৫.০ কমিরি থকেে বশেি দূরুত্বে >0.5 km	4	
202	স্বাস্থ্যকন্দ্রে আপনি কভিাবে যাতায়াত করে থাকনে? How do you ususally gop to nearest health care center?	পায়ে হটেে on foot	1	→205
		যানবাহন by vehicle	2	
		উভয়ই both	3	
203	স্বাস্থ্যকেন্দ্রে যাওয়ার জন্য আপনাকে কি কি ধরনের যানবাহন ব্যবহার করতে হয়?<br>What is/ are the mode of transportation you used to reach to the health facility?<br>উত্তর পড়ে শোনাবেন না।<br>Do not read out the answers<br>জিজ্ঞেস করুনঃ আরও কিছু?<br>ASK: Anything else?<br>সব উত্তরের কোড বৃত্তায়িত করুন।<br>Circle code of all the answers	রিকশা / ভ্যান Rickshaw/Rickshaw van	A	
		ব্যাটারী রিকশা/টমটম/অটোরিকশা battery rickshaw/scooter	B	
		নৌকা Boat	C	
		ইঞ্জিন চালিত নৌকা Engine boat	D	
		বাস / টেম্পু Bus/Tempo	E	
		গাড়ি / মাইক্রোবাস Car/Microbus	F	
		অন্যান্য Other (নির্দিষ্ট করুন) Specify ___________________________	Y	
204	আপনার বাড়ি থকেে নকিটবর্তী স্বাস্থ্যকন্দ্রে যাতায়াত করতে আপনার সাধারনত কত খরচ হয়? How much do you have to spend ususally to reach the health center and again come to home?	□□□□ টাকা		
205	বাসা থেকে বের হবার পর স্বাস্থ্যকেন্দ্রে পৌছানো পর্যন্ত মোট কত সময় লাগে? What is the total time needed counting from when the patient started from home and to when the patient reached the referral health facility?	□□:□□<br>ঘন্টা : মিনিট		

Section D: Pregnancy and ante natal care গর্ভ এবং গর্ভকালীন সেবা

No.	Questions and filters	Responses	Code	Skip
301	সাক্ষাতকারগ্রহণকারীঃ<br>মহিলার কি কোন গর্ভ ০১ জুন ২০১৬ থেকে ৩১ □□ ২০১৭ এর মধ্যে শেষ হয়েছে?<br>Was there any pregnancy ended between 01 June 2016 to 31st May 2017 for this woman?	হ্যাঁ Yes	1	
		না No	2	→end
302	সাক্ষাতকারগ্রহণকারীঃ 'শেষে গর্ভ কত মাস স্থায়ী হয়েছিল?' এবং সঠিক কোড বৃত্তায়িত করুন।<br>Ask duration of the last pregnancy of the woman?	3 মাস বা তার কম Pregnancy last less than 3 months	1	→end
		3 মাসের বেশি Pregnancy last more than 3 months	2	

সাক্ষাতকারগ্রহণকারীঃ ০১ জুন ২০১৬ থেকে ৩১ □□ ২০১৭ এর মধ্যে উত্তরদাতার সর্বশেষ গর্ভের গর্ভকালীন যত্ন সম্পর্কে জিজ্ঞেস করতে হবে, সুতরাং উত্তরদাতাকে সেই গর্ভ সম্পর্কে ভাল করে বুঝিয়ে তারপর প্রশ্ন জিজ্ঞেস করুন।

Interviewer: You have to collect information about the last pregnancy outcome (live-birth) during the period of 01 May 2015 to 30

April 2016. So, make sure that the respondent understand and identify the pregnancy then ask questions.				
303	এই গর্ভকালীন সময়ে চেকআপ করার জন্য আপনি কোন স্বাস্থ্যকর্মীর কাছে গিয়েছিলেন কি?<br>সাক্ষাৎকারগ্রহনকারীঃ উত্তরদাতাকে বুঝিয়ে বলুন স্বাস্থ্যকর্মী বলতে ডাক্তারসহ সব ধরনের স্বাস্থ্যকর্মীর কথাই আপনি জানতে চাচ্ছেন।<br>Did you see anyone for antenatal care for this pregnancy?	হ্যাঁ Yes<br>না No	1<br>2	
304	আপনি এই গর্ভের জন্য কতবার গর্ভকালীন সেবা (চেকআপ) করিয়েছেন?<br>How many times did you receive antenatal care (ANC) during this pregnancy?	বার times<br>জানিনা/মনে নাই Don't know	☐☐<br>97	→309

305 আপনি যখন এই গর্ভের জন্য গর্ভকালীন সেবা (চেকআপ) করিয়েছেন, তখন আপনি কত মাসের গর্ভবতী ছিলেন?

How many months pregnant were you when you received antenatal care for this pregnancy?

সঠিক মাস বলতে না পারলে, গর্ভ মাসের ঘরে '99' লিখুন।

Enter '99' if can't remember months pregnant

ANC visit ‡PKAvc	হ্যাঁ YES	না NO	গর্ভ মাস Months pregnant
ANC-1	1	2	☐☐
ANC-2	1	2	☐☐
ANC-3	1	2	☐☐
ANC-4	1	2	☐☐
ANC-5	1	2	☐☐
ANC-6	1	2	☐☐
ANC-7	1	2	☐☐
ANC-8	1	2	☐☐

306 আপনি কাকে দেখিয়েছিলেন?

Whom did you see?

প্রতিটি ANC এর জন্য জিজ্ঞাসা করুন

Ask for each ANC visit

ব্যক্তি সর্ম্পকে নিশ্চিত হোন এবং সঠিক উত্তরের কোড বৃত্তায়িত করুন

Probe to identify each type of person and record all mentioned.

স্বাস্থ্যসেবাদানকারী	ANC 1	ANC 2	ANC 3	ANC 4	ANC 5	ANC 6	ANC 7	ANC 8
**স্বাস্থ্য পেশাজীবি (Health Personnel)**								
পাশ করা ডাক্তার Qualified doctor	11	11	11	11	11	11	11	11
নার্স/ধাত্রী/প্যারামেডিক Nurse/midwife/ Paramedic	12	12	12	12	12	12	12	12
পরিবার কল্যাণ পরিদর্শিকা FWV	13	13	13	13	13	13	13	13
স্যাকমো SACMO	14	14	14	14	14	14	14	14
সি,এস,বি,এ CSBA	15	15	15	15	15	15	15	15
সি,এইচ, সি, পি CHCP	16	16	16	16	16	16	16	16
স্বাস্থ্য সহকারী HA	17	17	17	17	17	17	17	17
পরিবার কল্যান সহকারী FWA	18	18	18	18	18	18	18	18
**অন্যান্য স্বাস্থ্য সেবাপ্রদানকারী (Other)**								
প্রশিক্ষণ প্রাপ্ত টিবিএ (প্রশিক্ষণ প্রাপ্ত ধন্নী, চাউনী, দাই) TBA	21	21	21	21	21	21	21	21
প্রশিক্ষণহীন টিবিএ (ধন্নী, চাউনী, দাই) UTBA(Dai/Dhorni/Chauni)	22	22	22	22	22	22	22	22
সনাতন /পাশ না করা ডাক্তার Unqualified doctor	23	23	23	23	23	23	23	23
ঔষধ বিক্রেতা Drug seller	24	24	24	24	24	24	24	24
এনজিও কর্মী NGO worker	25	25	25	25	25	25	25	25
অন্যান্য (নির্দিষ্ট করুন) Other:__________	98	98	98	98	98	98	98	98
জানিনা/মনে নাই Don't know/Can't remember	99	99	99	99	99	99	99	99

307	এই গর্ভকালীন সময়ে মেডিকেল চেকআপের জন্য আপনি কোথায় গিয়েছিলেন?<br>Where did you receive antenatal care for this pregnancy?<br><br>প্রতিটি ANC এর জন্য জিজ্ঞাসা করুন<br>Ask for each ANC visit<br>প্রোব করে নিশ্চিত হোন কি ধরনের উৎসে গিয়েছিলেন এবং সঠিক কোড লিপিবদ্ধ করুন।<br>Probe to identify each type of source.<br>যদি নিশ্চিত হতে না পারেন যে এটা সরকারি, নাকি প্রাইভেট হাসপাতাল, ক্লিনিক বা স্বাস্থ্যকেন্দ্র তবে স্থানের নাম লিখে রাখুন<br>If unable to determine if public or private sector, write the name of the place:<br><br>______________________<br>স্থানের নাম<br>(Name of place)	গর্ভকালীন চেকআপের স্থান	ANC 1	ANC 2	ANC 3	ANC 4	ANC 5	ANC 6	ANC 7	ANC 8	
		**বাড়ি Home:**									
		বাড়িতে Home	11	11	11	11	11	11	11	11	
		**সরকারী সেক্টর Public sector:**									
		মেডিকেল কলেজ হাসপাতাল Medical College	21	21	21	21	21	21	21	21	
		বিশেষায়িত হাসপাতাল Specialized hospital	22	22	22	22	22	22	22	22	
		জেলা হাসপাতাল District hospital	23	23	23	23	23	23	23	23	
		মাতৃমঙ্গল কেন্দ্র Maternal & Child Welfare Centre (MCWC)	24	24	24	24	24	24	24	24	
		উপজেলা স্বাস্থ্য কমপ্লেক্স Upazila Health Complex	25	25	25	25	25	25	25	25	
		পরিবার কল্যাণ কেন্দ্র Family Welfare Centre (FWC)	26	26	26	26	26	26	26	26	
		স্যাটেলাইট ক্লিনিক/ইপিআই কেন্দ্র Satellite clinic/EPI centre	27	27	27	27	27	27	27	27	
		কমিউনিটি ক্লিনিক Community clinic	28	28	28	28	28	28	28	28	
		**এন জি ও সেক্টর NGO sector:**									
		এন জি ও স্থায়ী ক্লিনিক NGO static clinic	31	31	31	31	31	31	31	31	
		এন জি ও স্যাটেলাইট ক্লিনিক NGO satellite clinic	32	32	32	32	32	32	32	32	
		**প্রাইভেট সেক্টর Private sector:**									
		প্রাইভেট হাসপাতাল / ক্লিনিক Private hospital/Clinic	41	41	41	41	41	41	41	41	
		পাশ করা ডাক্তার MBBS doctor (Qualified)	42	42	42	42	42	42	42	42	
		অপ্রশিক্ষিত ডাক্তার (কোয়াক/ পল-ী চিকিৎসক/ হোমিওপ্যাথ) Quack/ Village doctor /Aiurved /Homeopath	43	43	43	43	43	43	43	43	
		ফার্মেসী Pharmacy	44	44	44	44	44	44	44	44	
		প্রাইভেট মেডিকেল কলেজ হাসপাতাল Private Medical College	46	46	46	46	46	46	46	46	
		অন্যান্য (নির্দিষ্ট করুন) Other: ______________________________	98	98	98	98	98	98	98	98	
		জানিনা/মনে নাই DK/Can't remember	99	99	99	99	99	99	99	99	
308	গর্ভকালীন চেকআপের সময় নিম্নের বিষয়ের কোনটি দেখা হয়েছিল? (প্রত্যেকটি বিষয় পড়ে শোনান এবং হয়ে থাকলে ANC ভিজিট অনুযায়ী বৃত্তায়িত করুন।)<br>As part of your antenatal	**বিষয় Item**	ANC 1	ANC 2	ANC 3	ANC 4	ANC 5	ANC 6	ANC 7	ANC 8	
		ওজন Weight	11	11	11	11	11	11	11	11	
		ব্লাড প্রেসার Blood pressure	12	12	12	12	12	12	12	12	
		প্রস্রাব পরীক্ষা Urine test	13	13	13	13	13	13	13	13	
		রক্ত পরীক্ষা Blood test	14	14	14	14	14	14	14	14	
		আলট্রাসনোগ্রাম Ultrasonogram	15	15	15	15	15	15	15	15	

	care during this pregnancy, were any of the following done? Circle if done by ANC visit.<br>1. ওজন weight<br>2. ব্লাড প্রেসার Blood pressure<br>3. প্রস্রাব পরীক্ষা Urine test<br>4. রক্ত পরীক্ষা Blood test<br>5. আলট্রাসনোগ্রাম Ultra sonogram<br>6. পেটে হাত দিয়ে পরীক্ষা Abdominal examination?	বিপদচিহ্ন সম্পর্কে কাউন্সেল Counselled on danger signs	16	16	16	16	16	16	16	16	
		পেটে হাত দিয়ে পরীক্ষা Abdominal examination	17	17	17	17	17	17	17	17	

309	আপনি কি এই গর্ভকালীন সময়ে (নাম পেটে থাকাকালীন) নবজাতকের ধনুষ্টংকার প্রতিরোধে টি,টি ইনজেকশন নিয়েছেন? During this pregnancy, were you given an injection in the arm to prevent the baby from getting tetanus, that is, convulsions after birth?	হ্যাঁ Yes	1	
		না No	2	→312
		জানিনা/মনে নাই Don't know/Can't remember	97	→312
310	এই গর্ভকালীন সময়ে আপনি কতবার টি,টি ইনজেকশন নিয়েছেন? During this pregnancy, how many times did you get a tetanus injection (TT)?	বার times	☐☐	
		জানিনা/মনে নাই Don't know	97	
311	সাক্ষাতকারগ্রহণকারীঃ প্রশ্ন 310 দেখুন এবং সঠিক কোড বৃত্তায়িত করুন। Check Q-310 and circle appropriate code	দুই বা ততোধিক 2 or more times	1	→315
		অন্যান্য Other	2	
312	এই গর্ভের পূর্বে আপনি কখনও টি,টি ইনজেকশন নিয়েছেন? At any time before this pregnancy, did you receive any tetanus injections (TT)?	হ্যাঁ Yes	1	
		না No	2	→315
		জানিনা/মনে নাই Don't know/Can't remember	97	→315
313	এই গর্ভের পূর্বে আপনি কতবার টি,টি ইনজেকশন নিয়েছেন? Before this pregnancy, how many times did you receive a tetanus injection?<br>৭ বার বা তার বেশী হলে '07' লিখুন। If 7 or more times, record '07'.	বার times	☐☐	
		জানিনা/মনে নাই Don't know	97	
314	এই গর্ভের কত বছর আগে আপনি শেষ টি,টি টিকাটি নিয়েছেন? How many years ago did you receive the last tetanus injection before this pregnancy?	বছর আগে Years ago	☐☐	
315	এই গর্ভকালীন সময়ে আপনি (শরীরে রক্ত হওয়ার জন্য) আয়রন ট্যাবলেট বা আয়রন সিরাপ খেয়েছিলেন কি? Did you take iron tablets or syrup during this pregnancy?	হ্যাঁ Yes	1	
		না No	2	→319
		জানিনা/মনে নাই Don't know/Can't remember	97	→319
316	এই গর্ভকালীন সময়ে আপনি মোট কত দিন আয়রন-ফলেট ট্যাবলেট/ সিরাপ খয়েছেনে? How many days did you take the iron tablets/syrup during your this pregnancy?	দিন Days ☐	☐☐	
		জানিনা/মনে নাই Don't know	999	
317	এই গর্ভকালীন সময়ে মোট কতগুলো আয়রন-ফলেট ট্যাবলেট অথবা আয়রন সিরাপ <u>পেয়েছিলেন অথবা কিনিছেনে</u>? How many tablets/bottles did you buy or receive during your this pregnancy?	সংখ্যক ট্যাবলেট number of tablets	☐☐☐	
		সংখ্যক বোতল number of bottles	☐☐☐	
		একটাও পাইনা Received none	998	
		জানিনা/মনে নাই Don't know	999	
318	যখন আপনি গর্ভবতী ছিলেন, তখন আপনি কোথা থেকে আয়রন-ফলেট ট্যাবলেট <u>পেয়েছিলেন অথবা কিনিছেনে</u>?	**<u>বাড়ী (Home)</u>**		
		বাড়ীতে, সরকারী স্বাস্থ্যকর্মীর কাছ থেকে (Duing GoB health worker's home visit)	11	
		বাড়ীতে, বেসরকারী / এনজিও স্বাস্থ্যকর্মীর কাছ থেকে (Duing Private/NGO health worker's home visit)	12	
		বাড়ীতে, আত্মীয়ের কাছ থেকে (From realtive)	13	

		**<u>সরকারী স্বাস্থ্য কেন্দ্র (Govt Health center)</u>**		
		মেডিকেল কলেজ হাসপাতাল Medical College	21	
		বিশেষায়িত হাসপাতাল Specialized hospital	22	
		জেলা হাসপাতাল District hospital	23	
		মাতৃমঙ্গল কেন্দ্র Maternal & Child Welfare Centre (MCWC)	24	
		উপজেলা স্বাস্থ্য কমপ্লেক্স Upazila Health Complex	25	
		পরিবার কল্যাণ কেন্দ্র Family Welfare Centre (FWC)	26	
		স্যাটেলাইট ক্লিনিক/ইপিআই কেন্দ্র Satellite clinic/EPI centre	27	
		কমিউনিটি ক্লিনিক Community clinic	28	
		অন্যান্য(নির্দিষ্ট করুন) Other: ____	29	
		**<u>এন জি ও সেকটর NGO sector:</u>**		
		এনজিও হাসপাতাল (NGO hospital)	31	
		এন জি ও স্থায়ী ক্লিনিক NGO static clinic	32	
		এন জি ও স্যাটেলাইট ক্লিনিক NGO satellite clinic	33	
		অন্যান্য(নির্দিষ্ট করুন) Other: __________________________	34	
		**<u>প্রাইভেট সেকটর Private sector:</u>**		
		প্রাইভেট হাসপাতাল / ক্লিনিক Private hospital/Clinic	41	
		পাশ করা ডাক্তারের চেম্বার MBBS doctor (Qualified)	42	
		গ্রাম ডাক্তারের চেম্বার (Village doctor's chamber)	43	
		সনাতন/অপ্রশিক্ষিত ডাক্তারের চেম্বার Traditional doctor	44	
		ফার্মেসী/ওষুধের দোকান Pharmacy	45	
		প্রাইভেট মেডিকেল কলেজ হাসপাতালPrivate Medical College	46	
		অন্যান্য(নির্দিষ্ট করুন) Other: ____	47	
319	**এই গর্ভকালীন সময়ে আপনি ক্যালসিয়াম ট্যাবলেট বা সিরাপ খেয়েছিলেন কি?** Did you take calcium tablets or syrup during this pregnancy?	হ্যাঁ Yes	1	
		না No	2	→323
		জানিনা/মনে নাই Don't know/Can't remember	97	→323
320	**এই গর্ভকালীন সময়ে** আপনি মোট কত দিন **ক্যালসিয়াম ট্যাবলেট বা সিরাপ** খেয়েছেন? How many days did you take the calcium tablets/syrup during your this pregnancy?	দিন Days	☐☐☐	
		জানিনা/মনে নাই Don't know	999	
321	**এই গর্ভকালীন সময়ে** (পুরোটা সময়) মোট কতগুলো **ক্যালসিয়াম ট্যাবলেট বা সিরাপ <u>পেয়েছিলেন অথবা কিনিছেনে</u>**? How many calcium tablets/bottles did you buy or receive during your this pregnancy?	সংখ্যক ট্যাবলেট number of tablets	☐☐☐	
		সংখ্যক বোতল number of bottles	☐☐☐	
		একটাও পাইনি Received none	998	
		জানিনা/মনে নাই Don't know	999	
322	যখন আপনি গর্ভবতী ছিলেন, তখন আপনি কোথা থেকে **ক্যালসিয়াম ট্যাবলেট বা সিরাপ <u>পেয়েছিলেন অথবা কিনিছেনে</u>**? [মহিলাকে জিজ্ঞেস করুন] আরও কিছু? [মহিলার নিজে থেকে দেয়া সবগুলো উত্তরই বৃত্তায়িত করুন । উত্তরগুলো পড়ে শুনাবেন না। একাধিক উত্তর হতে পারে।] Where did you get this calcium tablets or syrup? [Do not read out	**<u>বাড়ী (Home)</u>**		
		বাড়ীতে, সরকারী স্বাস্থ্যকর্মীর কাছ থেকে (Duing GoB health worker's home visit)	11	
		বাড়ীতে, বেসরকারী / এনজিও স্বাস্থ্যকর্মীর কাছ থেকে (Duing Private/NGO health worker's home visit)	12	
		বাড়ীতে, আত্মীয়ের কাছ থেকে (From realtive)	13	
		**<u>সরকারী স্বাস্থ্য কেন্দ্র (Govt Health center)</u>**		
		মেডিকেল কলেজ হাসপাতাল Medical College	21	
		বিশেষায়িত হাসপাতাল Specialized hospital	22	
		জেলা হাসপাতাল District hospital	23	
		মাতৃমঙ্গল কেন্দ্র Maternal & Child Welfare Centre (MCWC)	24	

	the answers. Ask: Anything else? Circle all the answers]	উপজেলা স্বাস্থ্য কমপ্লেক্স Upazila Health Complex	25	
		পরিবার কল্যাণ কেন্দ্র Family Welfare Centre (FWC)	26	
		স্যাটেলাইট ক্লিনিক/ইপিআই কেন্দ্র Satellite clinic/EPI centre	27	
		কমিউনিটি ক্লিনিক Community clinic	28	
		অন্যান্য(নির্দিষ্ট করুন) Other:_______________________________	29	
		**এন জি ও সেকটর NGO sector:**		
		এনজিও হাসপাতাল (NGO hospital)	31	
		এন জি ও স্থায়ী ক্লিনিক NGO static clinic	32	
		এন জি ও স্যাটেলাইট ক্লিনিক NGO satellite clinic	33	
		অন্যান্য(নির্দিষ্ট করুন) Other: ________________________	34	
		**প্রাইভেট সেকটর Private sector:**		
		প্রাইভেট হাসপাতাল / ক্লিনিক Private hospital/Clinic	41	
		পাশ করা ডাক্তারের চেম্বার MBBS doctor (Qualified)	42	
		গ্রাম ডাক্তারের চেম্বার (Village doctor's chamber)	43	
		সনাতন/অপ্রশিক্ষিত ডাক্তারের চেম্বার Traditional doctor	44	
		ফার্মেসী/ওষুধের দোকান Pharmacy	45	
		প্রাইভেট মেডিকেল কলেজ হাসপাতালPrivate Medical College	46	
		অন্যান্য(নির্দিষ্ট করুন) Other:__________________________________	47	
323	গর্ভকালীন চেকআপের সময় (যে কোন ভিজিটে) স্বাস্থ্যকর্মী কখনও কি আপনাকে জন্মের পর পর নবজাতককে কি ভাবে যত্ন নিতে হয় সে কথা বলেছেন? During (any of) your antenatal care visit(s), were you told about how to take care of the newborn immediately after birth?	হ্যাঁ Yes	1	
		না No	2	→325
		জানিনা/মনে নাই Don't know/Can't remember	97	→325
324	আপনি কি মনে করতে পারেন যে স্বাস্থ্যকর্মী আপনাকে কি কি নবজাতকের যত্নের কথা বলেছেন যা জন্মের পর পর নিতে হয়?<br>What were you told about immediate newborn care?<br><br>উত্তর পড়ে শোনাবেন না।<br>Do not read out the answers<br>জিজ্ঞেস করুনঃ আরও কিছু?<br>ASK: Anything else?<br>সব উত্তরের কোড বৃত্তায়িত করুন।<br>Circle code of all the answers	জন্মের পর পরই বাচ্চাকে পরিস্কার করা/শুকিয়ে নেয়ার কথা Clean/dry the baby immediately after birth	A	
		জন্মের পর পরই বাচ্চাকে কাপড় দিয়ে মুড়িয়ে নেয়ার কথা Wrap the baby immediately after birth	B	
		ফুল পড়ার আগেই বাচ্চাকে বুকের দুধ খাওয়ানোর কথা Put the baby to breast before placenta delivery	C	
		নাভীর যত্নে ক্লোরোহেক্সিডিন ব্যবহার করা Use of chlorohexidine for umbilical care	D	
		ভারনিক্স / সাদা চামড়া না তোলার কথা Not to remove the vernix or the white skin	E	
		প্রসবের ৩ দিন পর প্রথম গোসল করানোর কথা First bath to be given 3 days after delivery	F	
		বুকের দুধ খাওয়ানোর আগে অন্য কিছু না দেয়ার কথা Not to give anything before breast milk	G	
		শুধুমাত্র বুকের দুধ খাওয়ানোর কথা Exclusive Breastfeeding	H	
		কম ওজনের বাচ্চাকে বুকে রেখেগরম রাখার কথা Skin-to-skin contact (KMC) for LBW baby	I	
		পরিবার পরিকল্পনার কথা About family planning	J	
		বুকের দুধ খাওয়ানোর মাধ্যমে প্রসব পরবর্তীকালে পরিবার পরিকল্পনা LAM	K	
		অন্যান্য (নির্দিষ্ট করুন) Others	X	
		জানিনা/মনে নাই Don't know/Can't remember	Y	
325	এই গর্ভকালীন সময়ে আপনার কোন সমস্যা বা জটিলতা হয়েছিল কি যার জন্য ডাক্তারী চিকিৎসার প্রয়োজন ছিল? During the pregnancy	হ্যাঁ Yes	1	
		না No	2	→
		জানিনা/মনে নাই Don't know/Can't remember	97	→

	with (NAME), did you develop any problem/complication that required medical treatment			
326	আপনার কি ধরণের সমস্যা বা জটিলতা হয়েছিল?	তীব্র মাথা ব্যাথা Severe Headache	A	
	Please tell me what was that problem or complication?	চোখে ঝাপসা দেখা Blurred Vision	B	
		গর্ভের বাচ্চার নড়াচড়া কমে যাওয়া/বন্ধ হওয়া Fetal movement reduced/absent	C	
		গর্ভের বাচ্চার নড়াচড়া কমে যাওয়া/বন্ধ হওয়া Fetal movement reduced/absent	D	
		মুখমন্ডলে পানি আসা/ফুলে যাওয়া Oedema of the face/swelling	E	
	উত্তর পড়ে শোনাবেন না।	হাতে পানি আসা/ফুলে যাওয়া Oedema of the hands/swelling	F	
	Do not read out the answers	খিঁচুনী/ফিট Convulsions/fits	G	
	জিজ্ঞেস করুনঃ আরও কিছু?	বাচ্চা হওয়ার রাস্তায় অতিরিক্ত রক্তস্রাব Excessive Vaginal Bleeding	H	
	ASK: Anything else?	তলপেটে তীব্র ব্যথা Severe abdominal pain	I	
	সব উত্তরের কোড বৃত্তায়িত করুন।	পায়ে পানি আসা Oedema of the legs	J	
	Circle code of all the answers	তীব্র জ্বর Fever	K	
		সময় পূর্ণ হওয়ার আগে পানি ভাঙ্গা Premature rupture of membrane	L	
		অচেতন হওয়া/জ্ঞান হারিয়ে ফেলা Loss of consciousness	M	
		কষ্ট করে শ্বাস নেয়া Difficulty breathing	N	
		প্রচন্ড দুর্বলতা Severe weakness	O	
		অতিরিক্ত বমি Excessive vomiting	P	
		অতিরিক্ত সাদা স্রাব Excessive whitish vaginal discharge	Q	
		অন্যান্য (নির্দিষ্ট করুন) Others	X	
		জানিনা/কিছুই উল্লেখ করেননি Don't know/None mentioned	Y	
327	এই সমস্যা/অসুবিধা/জটিলতার জন্য আপনি কোন চিকিৎসা করিয়েছেন কি?	হ্যাঁ Yes	1	
	Did you seek any sort of treatment for this problem/complication?	না No	2	→
		জানিনা/মনে নাই Don't know/Can't remember	97	→
328	আপনি কার কাছে চিকিৎসা করিয়েছেন?	পাশ করা ডাক্তার MBBS doctor (Qualified)	A	
	From whom you received treatment for this problem/complication?	নার্স/ ধাত্রী /প্যারামেডিক Nurse/Midwife/Paramedic	B	
		পরিবার কল্যাণ পরিদর্শিকা Family Welfare Visitor (FWV	C	
		কমিউনিটি ভিত্তিক দক্ষ ধাত্রী (CSBA)	D	
	জিজ্ঞেস করুনঃ আর কেউ? Anyone else?	কমিউনিটি ক্লিনিক ভিত্তিক স্বাস্থ্য সেবা প্রদানকারী (CHCP)	E	
	ব্যক্তি সর্ম্পকে নিশ্চিত হোন এবং সঠিক উত্তরের কোড বৃত্তায়িত করুন	চিকিৎসা সহকারী / উপসহকারী কমিউনিটি চিকিৎসা কর্মকর্তা Medical Assistant / SACMO	F	
	Probe to identify each type of person and check all mentioned.	স্বাস্থ্য সহকারী Health Assistant (HA)	G	
		পরিবার কল্যাণ সহকারী Family Welfare Assistant (FWA)	H	
		প্রশিক্ষণপ্রাপ্ত টিবিএ/দাই Trained TBA	I	
	যদি 'D' বৃত্তায়িত হয় তবে এর নাম লিখুন	অপ্রশিক্ষিত টিবিএ/দাই Untrained TBA	J	
	If 'D' mentioned, write the name of the CSBA:	অপ্রশিক্ষিত ডাক্তার (গ্রাম ডাক্তার/ হোমিওপ্যাথ/ কবিরাজ) Unqualified doctor	K	
		এন জি ও স্বাস্থ্যকর্মী NGO worker	L	
		পরিবারের সদস্য/আত্নীয়/প্রতিবেশী Family member/ Relative/ Neighbour	M	
	নাম Name:	ফার্মেসি Pharmacy	N	
	________________________________	অন্যান্য (নির্দিষ্ট করুন) Other	X	
		জানিনা/মনে নাই Don't know	Y	

329	এই চিকিৎসা আপনি কোথায় করিয়েছেন?<br>From where did you receive care for this problem/complication?<br><br>জিজ্ঞেস করুনঃ আর কোথাও? Anywhere else?<br><br>স্থান সর্ম্পকে নিশ্চিত হোন এবং সঠিক উত্তরের কোড বৃত্তায়িত করুন<br>Probe to identify each type of source.<br><br>নির্দিষ্ট কোডের বাহিরের কোন স্থান হলে, নীচে স্থানের নাম লিখুন<br>If unable to determine the place, write the name of the place below:<br><br>______________________<br>স্থানের নাম (Name of place)	বাড়িতে Home	A	→330
		**সরকারী সেকটর Public sector:**		
		মেডিকেল কলেজ হাসপাতাল Medical College	B	
		বিশেষায়িত হাসপাতাল<br>Specialized hospital	C	
		জেলা হাসপাতাল District hospital	D	
		মাতৃমঙ্গল কেন্দ্র Maternal & Child Welfare Centre (MCWC)	E	
		উপজেলা স্বাস্থ্য কমপ্লেক্স<br>Upazila Health Complex	F	
		পরিবার কল্যাণ কেন্দ্র<br>Family Welfare Centre (FWC)	G	
		স্যাটেলাইট ক্লিনিক/ইপিআই কেন্দ্র Satellite clinic/EPI centre	H	
		কমিউনিটি ক্লিনিক Community clinic	I	
		**এন জি ও সেকটর NGO sector:**		
		এন জি ও স্থায়ী ক্লিনিক NGO static clinic	J	
		এন জি ও স্যাটেলাইট ক্লিনিক NGO satellite clinic	K	
		**প্রাইভেট সেকটর Private sector:**		
		প্রাইভেট হাসপাতাল / ক্লিনিক Private hospital/Clinic	L	
		পাশ করা ডাক্তার MBBS doctor (Qualified)	M	
		অপ্রশিক্ষিত ডাক্তার (কোয়াক/ পল- ী চিকিৎসক/ হোমিওপ্যাথ) Quack/ Village doctor /Aiurved /Homeopath	N	
		ফার্মেসী Pharmacy	O	
		প্রাইভেট মেডিকেল কলেজ হাসপাতাল Private Medical College	P	
		অন্যান্য (নির্দিষ্ট করুন) Other: ___________________________	Q	
		অন্যান্য Other sources:		
		দোকান Shop	R	
		বন্ধু/আত্নীয় Friends/Relatives	S	
		টিবিএ / দাই TBA	T	
		অন্যান্য (নির্দিষ্ট করুন) Other ____________________________	X	

গর্ভকালীন জটিলতার চিকিৎসা বাবদ খরচ সমূহ Teatment cost in pregnancy period

No.	Questions and filters	Responses	Code	Skip
330	আপনার গর্ভকালীন সময়ে চিকিৎসার জন্য কি কোন খরচ হয়েছিল? Did you incur any cost for the treatment during pregnancy period?	হ্যাঁ Yes	1	
		না No	2	→400
এখন আমি আপনার কাছে সেবা গ্রহন বাবদ খরচ সমূহ সম্পর্কে বিস্তারিত জানতে চাইব। (ভিজিট অনুযায়ী উল্লেখ করুন)<br>I will now ask you details about the costs that you had incurred for care seeking (Please mention by visit).				

331	ডাক্তার বা সেবাপ্রদানকারীর ফি বাবদ আপনার	১ম ভিজিট<br>1st visit	২য় ভিজিট<br>2nd visit	৩য় ভিজিট<br>3rd visit	৪র্থ ভিজিট<br>4th visit	

	কোন খরচ হয়েছিল কি? হ্যাঁ হলে, ডাক্তার বা সেবাপ্রদানকারীর ফি বাবদ মোট কত টাকা খরচ হয়েছিল? (ভিজিট অনুযায়ী লিখুন)<br>Did you incur any cost for consulting a Doctor or a Service provider? How much did you pay to the doctor or service provider as fees?	হ্যাঁ Yes .... 1<br>না No .... 2<br>Tk.__________	হ্যাঁ Yes .... 1<br>না No .... 2<br>Tk.__________	হ্যাঁ Yes .... 1<br>না No .... 2<br>Tk.__________	হ্যাঁ Yes .... 1<br>না No .... 2<br>Tk.__________	
332	আপনাকে কি হাসপাতালে ভর্তি হতে হয়েছিল? আপনাকে কতদিন হাসপাতালে থাকতে হয়েছিল? বেড/কেবিন ভাড়া বাবদ কত খরচ হয়েছিল?<br>Were you admitted at the hospital? How long did you have to stay at the hospital? How much Did you pay for the bed/cabin?	১ম ভিজিট<br>1st visit	২য় ভিজিট<br>2nd visit	৩য় ভিজিট<br>3rd visit	৪র্থ ভিজিট<br>4th visit	
		হ্যাঁ Yes .... 1<br>না No .... 2<br>হ্যাঁ হলে, হাসপাতালে কতদিন ছিলেন?<br>Days of hospital stay<br>|___|___| Tk.__________	হ্যাঁ Yes .... 1<br>না No .... 2<br>হ্যাঁ হলে, হাসপাতালে কতদিন ছিলেন?<br>Days of hospital stay<br>|___|___| Tk.__________	হ্যাঁ Yes .... 1<br>না No .... 2<br>হ্যাঁ হলে, হাসপাতালে কতদিন ছিলেন?<br>Days of hospital stay<br>|___|___| Tk.__________	হ্যাঁ Yes .... 1<br>না No .... 2<br>হ্যাঁ হলে, হাসপাতালে কতদিন ছিলেন?<br>Days of hospital stay<br>|___|___| Tk.__________	
333	আপনার কি কোন অপারেশন হয়েছিল? অপারেশন বাবদ কত খরচ হয়েছিল?<br>Did you undergo any surgery? How much did you have to pay for operation?	১ম ভিজিট<br>1st visit	২য় ভিজিট<br>2nd visit	৩য় ভিজিট<br>3rd visit	৪র্থ ভিজিট<br>4th visit	
		হ্যাঁ Yes .... 1<br>না No .... 2<br>হ্যাঁ হলে, কত খরচ হয়েছিল?<br>Tk.__________	হ্যাঁ Yes .... 1<br>না No .... 2<br>হ্যাঁ হলে, কত খরচ হয়েছিল?<br>Tk.__________	হ্যাঁ Yes .... 1<br>না No .... 2<br>হ্যাঁ হলে, কত খরচ হয়েছিল?<br>Tk.__________	হ্যাঁ Yes .... 1<br>না No .... 2<br>হ্যাঁ হলে, কত খরচ হয়েছিল?<br>Tk.__________	
334	আপনাকে কি কোন ঔষধ /ইঞ্জেকশান/ স্যালাইন খেতে হয়েছিল?<br>এসব ক্রয় বাবদ কত খরচ হয়েছিল?<br>Did you take any drugs/ medicine/ injection/ saline? How much did you pay for buying them?	১ম ভিজিট<br>1st visit	২য় ভিজিট<br>2nd visit	৩য় ভিজিট<br>3rd visit	৪র্থ ভিজিট<br>4th visit	
		হ্যাঁ Yes .... 1<br>না No .... 2<br>হ্যাঁ হলে, কত খরচ হয়েছিল?<br>Tk.__________	হ্যাঁ Yes .... 1<br>না No .... 2<br>হ্যাঁ হলে, কত খরচ হয়েছিল?<br>Tk.__________	হ্যাঁ Yes .... 1<br>না No .... 2<br>হ্যাঁ হলে, কত খরচ হয়েছিল?<br>Tk.__________	হ্যাঁ Yes .... 1<br>না No .... 2<br>হ্যাঁ হলে, কত খরচ হয়েছিল?<br>Tk.__________	
335	হাসপাতালে যাওয়ার অথবা ফিরে আসার পথে যাতায়াত বাবদ কোন খরচ হয়েছিল কি? যাতায়াত বাবদ মোট কত খরচ হয়েছিল?<br>Did you incur any transport cost while travelling to and returning from the provider? How much did you pay for transport cost in total?	১ম ভিজিট<br>1st visit	২য় ভিজিট<br>2nd visit	৩য় ভিজিট<br>3rd visit	৪র্থ ভিজিট<br>4th visit	
		হ্যাঁ Yes .... 1<br>না No .... 2<br>হ্যাঁ হলে, কত খরচ হয়েছিল?<br>Tk.__________	হ্যাঁ Yes .... 1<br>না No .... 2<br>হ্যাঁ হলে, কত খরচ হয়েছিল?<br>Tk.__________	হ্যাঁ Yes .... 1<br>না No .... 2<br>হ্যাঁ হলে, কত খরচ হয়েছিল?<br>Tk.__________	হ্যাঁ Yes .... 1<br>না No .... 2<br>হ্যাঁ হলে, কত খরচ হয়েছিল?<br>Tk.__________	
336	এই সেবাগ্রহণ করার সময়ে খাবার অথবা পানীয় কিনে খেতে কোন খরচ হয়েছিল কি? খাবার অথবা পানীয় কিনে খেতে কত খরচ হয়েছিল?<br>Did you incur any cost on food or beverages? How much did you pay for food or beverages?	১ম ভিজিট<br>1st visit	২য় ভিজিট<br>2nd visit	৩য় ভিজিট<br>3rd visit	৪র্থ ভিজিট<br>4th visit	
		হ্যাঁ Yes .... 1<br>না No .... 2<br>হ্যাঁ হলে, কত খরচ হয়েছিল?<br>Tk.__________	হ্যাঁ Yes .... 1<br>না No .... 2<br>হ্যাঁ হলে, কত খরচ হয়েছিল?<br>Tk.__________	হ্যাঁ Yes .... 1<br>না No .... 2<br>হ্যাঁ হলে, কত খরচ হয়েছিল?<br>Tk.__________	হ্যাঁ Yes .... 1<br>না No .... 2<br>হ্যাঁ হলে, কত খরচ হয়েছিল?<br>Tk.__________	

337	চিকিৎসা সেবা নিতে যাওয়ার পথে অথবা সেখান থেকে ফিরে আসার পথে আপনাকে হোটেল বা অন্যকোথাও রাত কাটাতে হয়েছিল কি? হোটেল বা অন্যকোথাও থাকা বাবদ কত খরচ হয়েছিল? Did you spend night away from home either while going there or while coming back? Did you incur any cost for staying over? How much did you pay?	১ম ভিজিট<br>1st visit	২য় ভিজিট<br>2nd visit	৩য় ভিজিট<br>3rd visit	৪র্থ ভিজিট<br>4th visit	
		হ্যাঁ Yes .... 1<br>না No .... 2<br>হ্যাঁ হলে, কত খরচ হয়েছিল?<br>Tk.__________	হ্যাঁ Yes .... 1<br>না No .... 2<br>হ্যাঁ হলে, কত খরচ হয়েছিল?<br>Tk.__________	হ্যাঁ Yes .... 1<br>না No .... 2<br>হ্যাঁ হলে, কত খরচ হয়েছিল?<br>Tk.__________	হ্যাঁ Yes .... 1<br>না No .... 2<br>হ্যাঁ হলে, কত খরচ হয়েছিল?<br>Tk.__________	
338	আপনাকে কি রক্ত/মল/মুত্র/আল্ট্রাসনোগ্রাম পরীক্ষা করাতে হয়েছিল কি? এসব পরীক্ষা করাতে কত খরচ হয়েছিল?<br>Did you spend any money for blood/stool/urine test?<br>How much did you spend for each of these tests?<br>যদি উত্তর না জানা থাকে তবে 'DK' লিখুন<br>If not known then write'DK'	১ম ভিজিট<br>1st visit	২য় ভিজিট<br>2nd visit	৩য় ভিজিট<br>3rd visit	৪র্থ ভিজিট<br>4th visit	
		হ্যাঁ Yes .... 1<br>না No .... 2<br>১। রক্ত পরীক্ষা<br>Blood test<br>Tk.____________<br>২। মুত্র পরীক্ষা Urine test<br>Tk.____________<br>৩। মল পরীক্ষা Stool test<br>Tk.____________<br>৪। আল্ট্রাসনোগ্রাম<br>Ultra sonogram<br>Tk.____________	হ্যাঁ Yes .... 1<br>না No .... 2<br>১। রক্ত পরীক্ষা<br>Blood test<br>Tk.____________<br>২। মুত্র পরীক্ষা Urine test<br>Tk.____________<br>৩। মল পরীক্ষা Stool test<br>Tk.____________<br>৪। আল্ট্রাসনোগ্রাম<br>Ultra sonogram<br>Tk.____________	হ্যাঁ Yes .... 1<br>না No .... 2<br>১। রক্ত পরীক্ষা<br>Blood test<br>Tk.___________<br>২। মুত্র পরীক্ষা Urine test<br>Tk.___________<br>৩। মল পরীক্ষা Stool test<br>Tk.____________<br>৪। আল্ট্রাসনোগ্রাম<br>Ultra sonogram<br>Tk.__________	হ্যাঁ Yes .... 1<br>না No .... 2<br>১। রক্ত পরীক্ষা<br>Blood test<br>Tk.___________<br>২। মুত্র পরীক্ষা Urine test<br>Tk.___________<br>৩। ৩। মল পরীক্ষা Stool test<br>Tk.____________<br>৪। আল্ট্রাসনোগ্রাম<br>Ultra sonogram<br>Tk.___________	
339	আপনাকে কি স্যালাইন/রক্ত/অক্সিজেন দিতে হয়েছিল? এগুলো বাবদ কত খরচ হয়েছিল?<br>Did you spend any money for saline infusion or blood transfusion or oxygen inhalation during your stay at health facility? How much did you sopned for each of these?<br>যদি উত্তর না জানা থাকে তবে 'DK' লিখুন<br>If not known then write'DK'	১ম ভিজিট<br>1st visit	২য় ভিজিট<br>2nd visit	৩য় ভিজিট<br>3rd visit	৪র্থ ভিজিট<br>4th visit	
		হ্যাঁ Yes .... 1<br>না No .... 2<br>১। স্যালাইন Saline<br>Tk.__________<br>২। রক্ত<br>Blood transfusion<br>Tk.__________<br>৩। অক্সিজেন Oxygen<br>Tk.__________	হ্যাঁ Yes .... 1<br>না No .... 2<br>১। স্যালাইন Saline<br>Tk.__________<br>২। রক্ত<br>Blood transfusion<br>Tk.__________<br>৩। অক্সিজেন Oxygen<br>Tk.__________	হ্যাঁ Yes .... 1<br>না No .... 2<br>১। স্যালাইন Saline<br>Tk.__________<br>২। রক্ত<br>Blood transfusion<br>Tk.__________<br>৩। অক্সিজেন Oxygen<br>Tk.__________	হ্যাঁ Yes .... 1<br>না No .... 2<br>১। স্যালাইন Saline<br>Tk.__________<br>২। রক্ত<br>Blood transfusion<br>Tk.__________<br>৩। অক্সিজেন Oxygen<br>Tk.__________	
340	ওয়ার্ড বয়, এটেনডেন্ট (সাহায্যকারী)/ দাড়োয়ানদের উপহার/বকশিশ বাবদ কোন খরচ হয়েছিল কি? কত খরচ হয়েছিল?<br>Did you pay for tips to ward boy, attendant, gate keeper? How much cost did you incur?	১ম ভিজিট<br>1st visit	২য় ভিজিট<br>2nd visit	৩য় ভিজিট<br>3rd visit	৪র্থ ভিজিট<br>4th visit	
		হ্যাঁ Yes .... 1<br>না No .... 2<br>হ্যাঁ হলে, কত খরচ হয়েছিল?<br>Tk.__________	হ্যাঁ Yes .... 1<br>না No .... 2<br>হ্যাঁ হলে, কত খরচ হয়েছিল?<br>Tk.__________	হ্যাঁ Yes .... 1<br>না No .... 2<br>হ্যাঁ হলে, কত খরচ হয়েছিল?<br>Tk.__________	হ্যাঁ Yes .... 1<br>না No .... 2<br>হ্যাঁ হলে, কত খরচ হয়েছিল?<br>Tk.__________	
341	আপনার কোন সাহায্যকারী ছিল কি?<br>Did you have any attendant?	হ্যাঁ Yes .... 1<br>না No .... 2→	হ্যাঁ Yes .... 1<br>না No .... 2→	হ্যাঁ Yes .... 1<br>না No .... 2→	হ্যাঁ Yes .... 1<br>না No .... 2→	If 2 →<br>343
342	আপনার এটেনডেন্ট (সাহায্যকারী) আপনাকে	১ম ভিজিট<br>1st visit	২য় ভিজিট<br>2nd visit	৩য় ভিজিট<br>3rd visit	৪র্থ ভিজিট<br>4th visit	

	সঙ্গ দেওয়ার সময়কালীন কোন অর্থনৈতিক ক্ষতির সম্মুখিন হয়েছিল? (অর্থাৎ সাহায্যকারী কোন কাজ করে এবং আপনার সাথে থাকার কারনে সে পারিশ্রমিক থেকে বঞ্চিত) হয়ে থাকলে তার পরিমাণ?<br>Did your attendant lose any income as a result of time spent with you on seeking care? If yes, how much did he/she lose?	হ্যাঁ Yes .... 1<br>না No .... 2<br>হ্যাঁ হলে, কত টাকা থেকে বঞ্চিত হয়েছিল?<br>Tk.__________	হ্যাঁ Yes .... 1<br>না No .... 2<br>হ্যাঁ হলে, কত টাকা থেকে বঞ্চিত হয়েছিল?<br>Tk.__________	হ্যাঁ Yes .... 1<br>না No .... 2<br>হ্যাঁ হলে, কত টাকা থেকে বঞ্চিত হয়েছিল?<br>Tk.__________	হ্যাঁ Yes .... 1<br>না No .... 2<br>হ্যাঁ হলে, কত টাকা থেকে বঞ্চিত হয়েছিল?<br>Tk.__________	

343	আপনার এই চিকিৎসাকালীন সময়ে যে সকল খরচ হয়েছিল, তা কিভাবে মিটিয়েছিলেন?<br>(একাধিক উত্তর গ্রহণযোগ্য)<br>How did you meet the expenditure that was incurred due to careseeking for your treatment?<br>(multiple answers acceptable)	সুদ সহ ঋণ এর মাধ্যমে/ধার করে Loan with Interest	A	→**for any option other than A & B go to next section**
		সুদ বিহীন ঋণ এর মাধ্যমে/ধার করে Loan without interest	B	
		সঞ্চয় থেকে বা বাড়ীর অন্যান্য খরচ বাচিয়ে Savings after all household expenses	C	
		সম্পত্তি বিক্রয় করে Selling land and assets	D	
		আত্মীয় / বন্ধু এর কাছ থেকে সাহায্য হিসাবে Help from Relatives/ Friends	E	
		নিয়মিত আয় থেকে Regular Income	F	
		নিজস্ব জিনিস বিক্রি করে Sold Personal Belongings	G	
		গৃহ পালিত পশু বিক্রি করে Sold Livestock.	H	
		গাছ অথবা উৎপাদিত শস্য বিক্রি কওে Sold agricultural product/tree	I	
		স্থায়ী সম্পদ বিক্রি করে Sold permanent assets	J	
		জমি বা সম্পদ জামানত রেখে Mortgage of Assets/land	K	
		অন্যান্য, উল্লেখ করচ্ন Others Specify ________________________________	X	
344	আপনি কোথা থেকে ধার করেছিলেন/ঋণ নিয়েছিলেন?<br>Where did you take the loan from?<br>(একাধিক উত্তর গ্রহণযোগ্য)<br>(multiple answers acceptable)	মাইক্রোক্রেডিট সংস্থা (এন জি ও) Microcredit (NGO) ব্যাংক Bank	A	
		গ্রামের মহাজন Village Mohajon	B	
		আত্মীয় Relative	C	
		প্রতিবেশী Neighbour	D	
		গ্রামের লোক Villagers	E	
		অন্যান্য, উল্লেখ করুন Others Specify ____________________________	X	
345	আপনি মোট কত টাকা ধার করেছিলেন/ঋণ নিয়েছিলেন?<br>How much money did you borrow?	☐☐☐☐☐☐ টাকা		
346	এই ঋণ এ সুদ এরহার কত ধরা হয়েছিল?<br>What was the rate of interest?<br>(কোন সুদ না থাকলে 00 এবং জানিনা হলে পুণরায় জিজ্ঞাসা করুন যে পরিবারের কেউ জানে কিনা, কেউ জানলে তার কাছ থেকে শুনে লিখুন, না হলে 99 লিখুন)<br>(put 00 if no interest, if answer is "don't know" ask whether anyone else in the family would know, write down the amount if anyone else knows, otherwise put 99)	১০০ টাকায় ☐☐ টাকা মাসিক/ বাৎসরিক		

Section E: Delivery প্রসব

No.	Questions and filters	Responses	Code	Skip
400	সাক্ষাতকারগ্রহনকারীঃ মহিলার সর্বশেষ গর্ভের ফলাফল সম্পর্কে সঠিক কোড বৃত্তায়িত করুন। What was the outcome of her last pregnancy?	জীবিত জন্ম Live birth	1	
		মৃত জন্ম Still birth	2	
		৭ মাসের আগে নষ্ট Lost before 7 months	3	→409
এখন আমি আপনার ০১ মে ২০১৫ থেকে ৩০ এপ্রিল ২০১৬ এর মধ্যে হওয়া সর্বশেষ ডেলিভারি সম্পর্কে কিছু প্রশ্ন জিজ্ঞেস করব। Now I shall ask few questions about delivery of (NAME) born between 01 May 2015 to 30 April 2016				
401	ডেলিভারি টি কোথায় হয়েছিল? Where did the birth /delvery take place?	বাড়ি Home	11	
		সরকারী স্বাস্থ্য কেন্দ্র Public sector:		
		কমিউনিটি ক্লিনিক Community clinic	21	
		ইউনিয়ন স্বাস্থ্য ও পরিবার কল্যাণ কেন্দ্র H&FWC	22	
		উপজেলা স্বাস্থ্য কমপ্লেক্স Upazila Health Complex (UHC)	23	
		জেলা হাসপাতাল District hospital	24	
		মেডিকেল কলেজ হাসপাতাল Medical College Hospital	25	
		মাতৃমঙ্গল কেন্দ্র MCWC / Maternity centre	26	
		অন্যান্য সরকারী স্বাস্থ্য কেন্দ্র_ Other public health centre	27	
		এন,জি,ও/প্রাইভেট স্বাস্থ্য কেন্দ্র NGO/Private health centre		
		এন জি ও ক্লিনিক/হাসপাতাল NGO clinic/hospital	31	
		প্রাইভেট হাসপাতাল/ক্লিনিক Private hospital/clinic	32	
		স্বাস্থ্য কেন্দ্র ছাড়া অন্য কোথাও (নির্দিষ্ট করুন) Others___________	33	
402	ডেলিভারি কে করিয়েছিলেন? Who assisted with the delivery of (NAME)? সাক্ষাতকারগ্রহণকারীঃ ডেলিভারিতে প্রধানত যিনি সাহায্য করেছিলেন অর্থ্যাৎ যার ভূমিকা সবচেয়ে বেশি ছিল শুধুমাত্র তার নাম লিখুন এবং তার কোড বৃত্তায়িত করুন। নামঃ___________________________ *শুধুমাত্র একটি কোড বৃত্তায়িত করুন।* Record the name who was mainly responsible or mostly involved in assisting the delivery. Interviewer: Ask and record the name of the person who assisted in delivery Name: ____	স্বাস্থ্য পেশাজীবিঃ Health personnel:		
		পাশ করা ডাক্তার MBBS doctor (Qualified)	11	
		নার্স/ ধাত্রী /প্যারামেডিক Nurse/Midwife/Paramedic	12	
		পরিবার কল্যাণ পরিদর্শিকা Family Welfare Visitor (FWV)	13	
		চিকিৎসা সহকারী / উপসহকারী কমিউনিটি চিকিৎসা কর্মকর্তা Medical Assistant / SACMO	14	
		কমিউনিটি ভিত্তিক দক্ষ ধাত্রী (CSBA)	15	
		কমিউনিটি ক্লিনিক ভিত্তিক স্বাস্থ্য সেবা প্রদানকারী (CHCP)	16	
		স্বাস্থ্য সহকারী Health Assistant (HA)	17	
		পরিবার কল্যাণ সহকারী Family Welfare Assistant (FWA)	18	
		অন্যান্য ব্যক্তিঃ Other person:		
		প্রশিক্ষণপ্রাপ্ত টিবিএ Trained TBA	21	
		অপ্রশিক্ষিত টিবিএ Untrained TBA	22	
		অপ্রশিক্ষিত ডাক্তার (গ্রাম ডাক্তার/ পলস্নী চিকিৎসক/হোমিওপ্যাথ/ কবিরাজ) Unqualified doctor	23	
		এন জি ও স্বাস্থ্যকর্মী NGO worker	24	
		পরিবারের সদস্য/আত্মীয় Family member / Relative	25	

	Circle only one from the list	প্রতিবেশী/বন্ধু Neighbour / Friend	26	
		অন্যান্য (নির্দিষ্ট করুন) Other: ___________________	27	
		জানিনা/মনে নাই Don't know/Can't remember		
403	প্রসবের ধরন কি ছিল? What was the mode of delivery?	স্বাভাবিক প্রসব Normal delivery	1	
		ফরসেপস ডেলিভারি Forceps delivery	2	
		ভ্যাকুয়াম/ ভেনটোস Vacuum/Ventouse	3	
		সিজারিয়ান সেকশন Caesarean section	4	
404	সাক্ষাতকারগ্রহনকারীঃ প্রশ্ন 401 দেখুন এবং ডেলিভারিটি কোথায় হয়েছে তা বৃত্তায়িত করুন। Check question 401 and circle code of place of delivery.	বাড়িতে At Home (Code 01)	1	
		স্বাস্থ্যকেন্দ্রে At Health Facility (Code 02-10)	2	→409
		অন্যান্য Any other place (Code 96)	3	
405	ডেলিভারিতে যিনি সাহায্য করেছিলেন, ডেলিভারির পূর্বে তিনি তার হাত সাবান দিয়ে ধুয়েছিলেন কি? Did the person who assisted with the delivery of (NAME) wash hands with soap prior to delivery?	হ্যাঁ Yes	1	
		না No	2	
		জানিনা/মনে নাই Don't know/Can't remember	97	
406	আপনার কি ডেলিভারি ব্যাগ/কিট ছিল? Did you have a delivery bag / birth kit?	হ্যাঁ Yes	1	
		না No	2	→409
		জানিনা/মনে নাই Don't know/Can't remember	97	→409
407	ডেলিভারি ব্যাগ/কিট কোথায় পেয়েছেন? Where did you get birth kit?	কমিউনিটি ক্লিনিক Community clinic	01	
		ইউনিয়ন স্বাস্থ্য ও পরিবার কল্যাণ কেন্দ্র (H&FWC)	02	
		স্যাটেলাইট ক্লিনিক Satellite clinic	03	
		কমিউনিটি ভিত্তিক দক্ষ ধাত্রী (CSBA)	04	
		উপজেলা স্বাস্থ্য কমপ্লেক্স Upazila Health Complex (UHC)	05	
		জেলা হাসপাতাল District hospital	06	
		মেডিকেল কলেজ হাসপাতাল Medical College Hospita..........	07	
		মাতৃমঙ্গল কেন্দ্র MCWC / Maternity centre	08	
		প্রাইভেট হাসপাতাল/ ক্লিনিক Private hospital/clinic	09	
		এন জি ও ক্লিনিক NGO clinic	10	
		স্বাস্থ্য সেবিকা (ব্রাক) BRAC Health volunteer	11	
		টিবিএ (দাই) Traditional Birth Attendant (TBA)	12	
		দোকান Shop	13	
		অন্যান্য (নির্দিষ্ট করুন) Other: ___________________	96	
		জানিনা/মনে নাই Don't know/Can't remember	97	

408	ডেলিভারি ব্যাগ/কিট এর কি কি জিনিস আপনার ডেলিভারির সময় ব্যবহার করা হয়েছিল? What were the items from the birth kit that were used during the delivery? উত্তরদাতা স্বত:স্ফূর্তভাবে যে সব জিনিসের নাম উল্লেখ করবেন তার কোড প্রথম কলামে বৃত্তায়িত করুন অতঃপর যেগুলি বলবেন না,		স্বত:স্ফূর্ত ভাবে বলেছে Stated promptly	দেখানোর পর হ্যাঁ বলেছে Stated after showing	জানিনা/ না Not stated	
		প্লাস্টিক শিট Plastic sheet	1	2	3	
		ব্লেড Blade	1	2	3	

	CDK এর জিনিসগুলি এক এক করে দেখান এবং জিজ্ঞেস করুন এটি ব্যবহার করা হয়েছিল কি-না, উত্তর হ্যাঁ হলে দ্বিতীয় কলামে, না হলে তৃতীয় কলামে কোড বৃত্তায়িত করুন।	সুতা Thread	1	2	3	
		সাবান Soap	1	2	3	
409	আপনার ডেলিভারির সময় কোন সমস্যা বা জটিলতা হয়েছিল? During the time of the birth of (Name) or abortion did you have any problem or complication? (প্রত্যেকটি সমস্যা/জটিলতা সম্পর্কে জিজ্ঞেস করুন) (Ask all of the problems/complications)			Yes	No	
		সমস্যা/জটিলতা Problem/Complication		1	2	
		বাচ্চা হওয়ার রাস্তা দিয়ে অতিরিক্ত রক্ত গিয়েছিল Excessive Vaginal Bleeding		1	2	
		দুর্গন্ধযুক্ত স্রাব গিয়েছিল Foul-Smelling Discharge		1	2	
		তীব্র জ্বর হয়েছিল High Fever		1	2	
		শিশুর হাত পা আগে বের হয়ে এসেছিল Baby's Hand or Feet Coming out First		1	2	
		(পেটের মধ্যে) শিশুর অস্বাভাবিক অবস্থান ছিল Baby is in abnormal position		1	2	
		দীর্ঘ প্রসব (১২ ঘন্টার বেশি) ব্যথা ছিল Prolong Labor (>12 hours)		1	2	
		পানি ভেঙ্গেছে কিন্তু ব্যাথা উঠেনি Membrane ruptured but no labour pain		1	2	
		প্লাসেন্টা বা ফুল পড়ে নি Retained Placenta		1	2	
		বাচ্চা থাকার থলি বা গর্ভদানী বা জন্ম দ্বার ছিঁড়ে গিয়েছিল Rupture uterus/Cervical tear/Vaginal tear		1	2	
		(শিশুর) নাড়ী বেরিয়ে এসেছিল Cord Prolapse		1	2	
		(শিশুর গলায়) নাড়ী পেঁচিয়ে গিয়েছিল Cord around neck		1	2	
		খিঁচুনী হয়েছিল Convulsion		1	2	
		তীব্র মাথা ব্যথা হয়েছিল Severe headache		1	2	
		বাচ্চা হওয়ার রাস্তা দিয়ে সবুজাভ কিছু বের হয়েছিল Greenish vaginal discharge		1	2	
		পা/মুখ ফুলে গিয়েছিল Swelling of feet or face		1	2	
		আরও কোন সমস্যা হয়েছিল Any other problem (specify) ___________________________		1	2	
410	সাক্ষাতকারগ্রহনকারীঃ প্রশ্ন 409 দেখুন এবং সঠিক কোড বৃত্তায়িত করুন। Interviewer: Check Question 409 and circle appropriate code.	এক বা একাধিক কোড 1 বৃত্তায়িত One or more codes circled			1	
		সবগুলো কোড 2 বৃত্তায়িত All codes circled			2	→500
411	এই সমস্যা/জটিলতার জন্য আপনি কোন চিকিৎসা করিয়েছেন কি? Did you seek treatment for this complication?	হ্যাঁ Yes			1	
		না No			2	→500
		জানিনা/মনে নাই Don't know/Can't remember			97	→500
412	আপনি কার কার কাছ থেকে চিকিৎসা নিয়েছেন? Whom did you see? উত্তর পড়ে শোনাবেন না।	স্বাস্থ্য পেশাজীবিঃ Health personnel:				
		পাশ করা ডাক্তার MBBS doctor (Qualified)			A	
		নার্স/ ধাত্রী /প্যারামেডিক Nurse/Midwife/Paramedic			B	
		পরিবার কল্যাণ পরিদর্শিকা Family Welfare Visitor (FWV)			C	

	Do not read out the answers<br>জিজ্ঞেস করুনঃ আরও কিছু?<br>ASK: Anything else?<br>সব উত্তরের কোড বৃত্তায়িত করুন।<br>Circle code of all the answers	চিকিৎসা সহকারী / উপসহকারী কমিউনিটি চিকিৎসা কর্মকর্তা Medical Assistant / SACMO	D	
		কমিউনিটি ভিত্তিক দক্ষ ধাত্রী (CSBA)	E	
		কমিউনিটি ক্লিনিক ভিত্তিক স্বাস্থ্য সেবা প্রদানকারী (CHCP)	F	
		স্বাস্থ্য সহকারী Health Assistant (HA)	G	
		পরিবার কল্যাণ সহকারী Family Welfare Assistant (FWA)	H	
		<u>অন্যান্য ব্যক্তিঃ Other person:</u>		
		প্রশিক্ষণপ্রাপ্ত টিবিএ Trained TBA	I	
		অপ্রশিক্ষিত টিবিএ Untrained TBA	J	
		অপ্রশিক্ষিত ডাক্তার (গ্রাম ডাক্তার/ পল্লী চিকিৎসক/হোমিওপ্যাথ/ কবিরাজ) Unqualified doctor	K	
		এন জি ও স্বাস্থ্যকর্মী NGO worker	L	
		পরিবারের সদস্য/আত্মীয় Family member / Relative	M	
		প্রতিবেশী/বন্ধু Neighbour / Friend	N	
		অন্যান্য (নির্দিষ্ট করুন) Other: ___________________	X	
		জানিনা/মনে নাই Don't know/Can't remember	Y	
413	এই চিকিৎসা আপনি কোথায় করিয়েছেন?<br>Where did you seek treatment for the complication?	বাড়িতে At home	A	→414
		<u>সরকারী সেক্টরঃ Govt. Sector:</u>		
		হাসপাতাল/মেডিকেল কলেজ Hospital/Medical college	B	
		স্বাস্থ্য ও পরিবার কল্যাণ কেন্দ্র H&FWC	C	
		উপজেলা স্বাস্থ্য কমপ্লেক্স Upazila Health Complex	D	
		স্যাটেলাইট ক্লিনিক Satellite clinic/ EPI centre	E	
		মাতৃমঙ্গল কেন্দ্র MCWC / Maternity centre	F	
		পরিবার কল্যাণ সহকারী FWA	G	
		কমিউনিটি দক্ষ ধাত্রী CSBA	H	
		কম্যুউনিটি ক্লিনিক Community clinic	I	
		অন্যান্য (নির্দিষ্ট করুন) Other:	J	
		<u>এন জি ও সেক্টরঃ NGO Sector:</u>		
		এন জি ও স্থায়ী কেন্দ্র NGO Static Clinic	K	
		এন জি ও অস্থায়ী কেন্দ্র NGO Satellite Clinic	L	
		অন্যান্য (নির্দিষ্ট করুন) Other:	M	
		<u>প্রাইভেট মেডিকেল সেক্টরঃ Private Medical Center:</u>		
		প্রাইভেট ক্লিনিক/হাসপাতাল Private clinic/Hospital	N	
		পাশকরা ডাক্তার Qualified doctor (MBBS)	O	
		অপ্রশিক্ষিত ডাক্তার (গ্রাম ডাক্তার/কবিরাজ) Unqualified doctor	P	
		ফার্মেসী Pharmacy	Q	
		অন্যান্য (নির্দিষ্ট করুন) Other:	R	
		<u>অন্যান্য Other sources:</u>		

		দোকান Shop	S	
		বন্ধু/আত্মীয় Friends/Relatives	T	
		টিবিএ / দাই TBA	U	
		অন্যান্য (নির্দিষ্ট করুন) Other ___________________________	X	

প্রসবকালীন (ডেলিভারী সংক্রান্ত) জটিলতায় চিকিৎসা বাবদ খরচ সমূহ **Teatment cost for delivery complication**

No.	Questions and filters	Responses		Code	Skip
414	আপনার প্রসবকালীন (ডেলিভারীর) সময়ে চিকিৎসার জন্য কি কোন খরচ হয়েছিল? Did you incur any cost for the treatment during delivery period?	হ্যাঁ Yes		1	
		না No		2	→500
এখন আমি আপনার কাছে সেবা গ্রহন বাবদ খরচ সমূহ সম্পর্কে বিস্তারিত জানতে চাইব। (ভিজিট অনুযায়ী উল্লেখ করুন)<br>I will now ask you details about the costs that you had incurred for care seeking (Please mention by visit).					
415	ডাক্তার বা সেবাপ্রদানকারীর ফি বাবদ আপনার কোন খরচ হয়েছিল কি? হ্যাঁ হলে, ডাক্তার বা সেবাপ্রদানকারীর ফি বাবদ মোট কত টাকা খরচ হয়েছিল? Did you incur any cost for consulting a Doctor or a Service provider? How much did you pay to the doctor or service provider as fees?	১ম ভিজিট<br>1st visit	২য় ভিজিট<br>2nd visit		
		হ্যাঁ Yes .... 1<br>না No .... 2<br><br>Tk.__________	হ্যাঁ Yes........ 1<br>না No .... 2<br><br>Tk.__________		
416	ডেলিভারীর সরঞ্জাম (যেমন: বার্থ কিট, নতুন ব্লেড, কাপড়, সুতা ইত্যাদি) কেনা বাবদ আপনার কোন খরচ হয়েছিল কি?<br>Did you incur any cost to buy materials for delivery like; Birth-kit, new blade, new cloth, thread etc?	হ্যাঁ Yes .... 1<br>না No 2	হ্যাঁ হলে, কত টাকা?<br>Tk.____________		
417	আপনাকে কি হাসপাতালে ভর্তি হতে হয়েছিল? আপনাকে কতদিন হাসপাতালে থাকতে হয়েছিল? বেড/কেবিন ভাড়া বাবদ কত খরচ হয়েছিল?<br>Were you admitted at the hospital? How long did you have to stay at the hospital? How much Did you pay for the bed/cabin?	১ম ভিজিট<br>1st visit	২য় ভিজিট<br>2nd visit		
		হ্যাঁ Yes .... 1<br>না No .... 2<br>হ্যাঁ হলে, হাসপাতালে কতদিন ছিলেন?<br>Days of hospital stay |___|___| Tk.__________	হ্যাঁ Yes........ 1<br>না No .... 2<br>হ্যাঁ হলে, হাসপাতালে কতদিন ছিলেন?<br>Days of hospital stay<br>|___|___| Tk.__________		
418	আপনার কি কোন অপারেশন হয়েছিল? অপারেশন বাবদ কত খরচ হয়েছিল?<br>Did you undergo any surgery? How much did you have to pay for operation?	১ম ভিজিট<br>1st visit	২য় ভিজিট<br>2nd visit		
		হ্যাঁ Yes 1<br>না No 2<br>হ্যাঁ হলে, কত খরচ হয়েছিল?<br>Tk._______________	হ্যাঁ Yes 1<br>না No 2<br>হ্যাঁ হলে, কত খরচ হয়েছিল?<br>Tk._______________		
419	আপনাকে কি কোন ঔষধ /ইঞ্জেকশান/ স্যালাইন খেতে হয়েছিল? এসব ক্রয় বাবদ কত খরচ হয়েছিল?<br>Did you take any drugs/ medicine/ injection/ saline? How much did you pay for buying them?	১ম ভিজিট<br>1st visit	২য় ভিজিট<br>2nd visit		
		হ্যাঁ Yes 1<br>না No 2<br>হ্যাঁ হলে, কত খরচ হয়েছিল?	হ্যাঁ Yes 1<br>না No 2<br>হ্যাঁ হলে, কত খরচ হয়েছিল?		

		Tk.______________	Tk.______________	
420	হাসপাতালে যাওয়ার অথবা ফিরে আসার পথে যাতায়াত বাবদ কোন খরচ হয়েছিল কি? যাতায়াত বাবদ মোট কত খরচ হয়েছিল?<br>Did you incur any transport cost while travelling to and returning from the provider? How much did you pay for transport cost in total?	১ম ভিজিট<br>1st visit<br>হ্যাঁ Yes 1<br>না No 2<br>হ্যাঁ হলে, কত খরচ হয়েছিল?<br>Tk.______________	২য় ভিজিট<br>2nd visit<br>হ্যাঁ Yes 1<br>না No 2<br>হ্যাঁ হলে, কত খরচ হয়েছিল?<br>Tk.______________	
421	এই সেবাগ্রহণ করার সময়ে খাবার অথবা পানীয় কিনে খেতে কোন খরচ হয়েছিল কি? খাবার অথবা পানীয় কিনে খেতে কত খরচ হয়েছিল?<br>Did you incur any cost on food or beverages? How much did you pay for food or beverages?	১ম ভিজিট<br>1st visit<br>হ্যাঁ Yes 1<br>না No 2<br>হ্যাঁ হলে, কত খরচ হয়েছিল?<br>Tk._______________	২য় ভিজিট<br>2nd visit<br>হ্যাঁ Yes 1<br>না No 2<br>হ্যাঁ হলে, কত খরচ হয়েছিল?<br>Tk.______________	
422	চিকিৎসা সেবা নিতে যাওয়ার পথে অথবা সেখান থেকে ফিরে আসার পথে আপনাকে হোটেল বা অন্যকোথাও রাত কাটাতে হয়েছিল কি? হোটেল বা অন্যকোথাও থাকা বাবদ কত খরচ হয়েছিল?<br>Did you spend night away from home either while going there or while coming back? Did you incur any cost for staying over? How much did you pay?	১ম ভিজিট<br>1st visit<br>হ্যাঁ Yes 1<br>না No 2<br>হ্যাঁ হলে, কত খরচ হয়েছিল?<br>Tk._________	২য় ভিজিট<br>2nd visit<br>হ্যাঁ Yes 1<br>না No 2<br>হ্যাঁ হলে, কত খরচ হয়েছিল?<br>Tk._________	
423	আপনাকে কি রক্ত/মল/মুত্র/আলট্রাসনোগ্রাম পরীক্ষা করাতে হয়েছিল কি? এসব পরীক্ষা করাতে কত খরচ হয়েছিল?<br>Did you spend any money for blood/stool/urine test?<br>How much did you spend for each of these tests?<br>যদি উত্তর না জানা থাকে তবে 'DK' লিখুন<br>If not known then write'DK'	১ম ভিজিট<br>1st visit<br>হ্যাঁ Yes 1<br>না No 2<br>১। রক্ত পরীক্ষা Blood test<br>Tk.___________<br>২। মুত্র পরীক্ষা Urine test<br>Tk.___________<br>৩। মল পরীক্ষা Stool test<br>Tk.___________<br>৪। আলট্রাসনোগ্রাম Ultra sonogram<br>Tk.___________	২য় ভিজিট<br>2nd visit<br>হ্যাঁ Yes 1<br>না No 2<br>১। রক্ত পরীক্ষা Blood test<br>Tk.___________<br>২। মুত্র পরীক্ষা Urine test<br>Tk.___________<br>৩। মল পরীক্ষা Stool test<br>Tk.___________<br>৪। আলট্রাসনোগ্রাম Ultra sonogram<br>Tk.___________	
424	আপনাকে কি স্যালাইন/রক্ত/অক্সিজেন দিতে হয়েছিল? এগুলো বাবদ কত খরচ হয়েছিল?<br>Did you spend any money for saline infusion or blood transfusion or oxygen inhalation during your stay at health facility? How much	১ম ভিজিট<br>1st visit<br>হ্যাঁ Yes 1<br>না No 2	২য় ভিজিট<br>2nd visit<br>হ্যাঁ Yes 1<br>না No 2	

	did you sopned for each of these?<br><br>যদি উত্তর না জানা থাকে তবে ‘DK’ লিখুন<br>If not known then write’DK’	১। স্যালাইন Saline<br>Tk.__________<br>২। রক্ত Blood transfusion<br>Tk.__________<br>৩। অক্সিজেন Oxygen<br>Tk.__________	১। স্যালাইন Saline<br>Tk.__________<br>২। রক্ত Blood transfusion<br>Tk.__________<br>৩। অক্সিজেন Oxygen<br>Tk.__________	
425	ওয়ার্ড বয়, এটেনডেন্ট (সাহায্যকারী)/ দাড়োয়ানদের উপহার/বকশিশ বাবদ কোন খরচ হয়েছিল কি ? কত খরচ হয়েছিল?<br><br>Did you pay for tips to ward boy, attendant, gate keeper? How much cost did you incur?	১ম ভিজিট<br>1st visit<br><br>হ্যাঁ Yes 1<br>না No 2<br>হ্যাঁ হলে, কত খরচ হয়েছিল?<br>Tk._________	২য় ভিজিট<br>2nd visit<br><br>হ্যাঁ Yes 1<br>না No 2<br>হ্যাঁ হলে, কত খরচ হয়েছিল?<br>Tk._________	
426	আপনার কোন সাহায্যকারী ছিল কি?<br>Did you have any attendant?	হ্যাঁ Yes .... 1<br>না No......... 2	হ্যাঁ Yes........1<br>না No 2	If 2 → 427
	আপনার এটেনডেন্ট (সাহায্যকারী) আপনাকে সঙ্গ দেওয়ার সময়কালীন কোন অর্থনৈতিক ক্ষতির সম্মুখিন হয়েছিল? (অর্থাৎ সাহায্যকারী কোন কাজ করে এবং আপনার সাথে থাকার কারনে সে পারিশ্রমিক থেকে বঞ্চিত)<br>হয়ে থাকলে তার পরিমাণ?<br>Did your attendant lose any income as a result of time spent with you on seeking care? If yes, how much did he/she lose?	১ম ভিজিট<br>1st visit<br><br>হ্যাঁ Yes 1<br>না No 2<br>হ্যাঁ হলে, কত টাকা থেকে বঞ্চিত হয়েছিল?<br>Tk._____________	২য় ভিজিট<br>2nd visit<br><br>হ্যাঁ Yes 1<br>না No 2<br>হ্যাঁ হলে, কত টাকা থেকে বঞ্চিত হয়েছিল?<br>Tk._____________	

427	আপনার এই চিকিৎসাকালীন সময়ে যে সকল খরচ হয়েছিল, তা কিভাবে মিটিয়েছিলেন?<br><br>(একাধিক উত্তর গ্রহণযোগ্য)<br><br>How did you meet the expenditure that was incurred due to careseeking for your treatment?<br>(multiple answers acceptable)	সুদ সহ ঋণ এর মাধ্যমে/ধার করে Loan with Interest	A	→for any option other than A &B go to next section
		সুদ বিহীন ঋণ এর মাধ্যমে/ধার করে Loan without interest	B	
		সঞ্চয় থেকে বা বাড়ীর অন্যান্য খরচ বাচিয়ে Savings after all household expenses	C	
		সম্পত্তি বিক্রয় করে Selling land and assets	D	
		আত্মীয় / বন্ধু এর কাছ থেকে সাহায্য হিসাবে Help from Relatives/ Friends	E	
		নিয়মিত আয় থেকে Regular Income	F	
		নিজস্ব জিনিস বিক্রি করে Sold Personal Belongings	G	
		গৃহ পালিত পশু বিক্রি করে Sold Livestock.	H	
		গাছ অথবা উৎপাদিত শস্য বিক্রি কওে Sold agricultural product/tree	I	
		স্থায়ী সম্পদ বিক্রি করে Sold permanent assets	J	
		জমি বা সম্পদ জামানত রেখে Mortgage of Assets/land	K	
		অন্যান্য, উল্লেখ করুন Others Specify<br>______________________________	X	
428	আপনি কোথা থেকে ধার করেছিলেন/ঋণ নিয়েছিলেন?<br>Where did you take the loan from?<br><br>(একাধিক উত্তর গ্রহণযোগ্য)<br><br>(multiple answers acceptable)	মাইক্রোক্রেডিট সংস্থা (এন জি ও) Microcredit (NGO) ব্যাংক Bank	A	
		গ্রামের মহাজন Village Mohajon	B	
		আত্মীয় Relative	C	
		প্রতিবেশী Neighbour	D	
		গ্রামের লোক Villagers	E	
		অন্যান্য, উল্লেখ করুন Others Specify	X	

		______________________________		
429	আপনি মোট কত টাকা ধার করেছিলেন/ঋণ নিয়েছিলেন?<br>How much money did you borrow?	☐☐☐☐☐☐ টাকা		
430	এই ঋণ এ সুদ এরহার কত ধরা হয়েছিল?<br>What was the rate of interest?<br>(কোন সুদ না থাকলে 00 এবং জানিনা হলে পুণরায় জিজ্ঞাসা করুন যে পরিবারের কেউ জানে কিনা, কেউ জানলে তার কাছ থেকে শুনে লিখুন, না হলে 99 লিখুন)<br>(put 00 if no interest, if answer is "don't know" ask whether anyone else in the family would know, write down the amount if anyone else knows, otherwise put 99)	১০০ টাকায় ☐☐ টাকা মাসিক/ বাৎসরিক		

Section F: Immediate newborn care জন্মের পর পর নবজাতকের যত্ন

No.	Questions and filters	Responses	Code	Skip
500	সাক্ষাতকারগ্রহনকারীঃ মহিলার সর্বশেষ গর্ভের ফলাফল সম্পর্কে সঠিক কোড বৃত্তায়িত করুন। What was the outcome of her last pregnancy?	জীবিত জন্ম Live birth	1	
		মৃত জন্ম Still birth	2	→600
		৭ মাসের আগে নষ্ট Lost before 7 months	3	→600
501	ডেলিভারীর পর পরই বাচ্চার জন্য কিছু করণীয় থাকে। সে সম্পর্কে সুনির্দিষ্ট কিছু প্রশ্ন এখন আমি আপনাকে জিজ্ঞেস করব। অর্থাৎ (নাম) এর জন্মের পর পরই (নাম) কে কি করা হয়েছিল সে সম্পর্কে এখন আমি আপনাকে কিছু প্রশ্ন জিজ্ঞেস করব। After the birth of a baby there is something essential to do. Now I would ask you few questions regarding what was done immediately after the birth of (NAME)			
502	সাক্ষাতকারগ্রহনকারীঃ প্রশ্ন 401 দেখুন (ডেলিভারীর স্থান) এবং সঠিক কোড বৃত্তায়িত করুন। Check Question 401 and circle appropriate code	কোড 01 বা 96 বৃত্তায়িত Code 01 or 96 circled	1	
		কোড 02-10 বৃত্তায়িত Code 02-10 circled	2	→515
503	নাম _______ এর জন্মের সাথে সাথে অর্থাৎ পেট থেকে বের হওয়ার পর পরই কোন্ কাজটি প্রথম করা হয়েছিল? What was the very first thing done immediately after delivery? ভাল করে প্রোব করুন। Probe adequately শুধুমাত্র একটি উত্তর বৃত্তায়িত করুন। Circle only one answer.	নাড়ী কাটা Cut cord	01	
		মায়ের পেটের/বুকের উপর বাচ্চাকে রাখা Placed the child on mother's abdomen/ chest	02	
		একা ফেলে রাখা Left alone	03	
		গা শুকানো Dried	04	
		কাপড় দিয়ে মুড়ানো Wrapped	05	
		গোসল করানো Bathed	06	
		বাচ্চাকে ঘুমাতে দেয়া Let the baby sleep	07	
		বুকের দুধ খাওয়ানো Breast fed	08	
		চিনির পানি বা অন্য কিছু খাওয়ানো Fed sugar water or other thing	09	
		অন্যান্য (নির্দিষ্ট করুন) Other _______________	96	
		জানিনা/মনে নাই Don't know/Can't remember	97	
504	নাম _______ এর জন্মের কত মিনিট পর ফুল পড়েছিল? How many minutes after delivery of (Name) the placenta was delivered?	মিনিট Minutes	☐☐	
		ফুল পড়ে নি, আমাকে হাসপাতালে নিয়ে যাওয়া হয়েছিল Placenta was not delivered, I was moved to the hospital	95	
		জানি না Don't know	97	
505	নাম _______ কে কখন মোছানো বা শুকানো হয়েছিল, ফুল পড়ার আগে না-কি ফুল পড়ার পরে? When the baby's body was wiped (dried), before delivery of placenta or after delivery of placenta?	ফুল পড়ার আগে Before delivery of placenta	1	
		ফুল পড়ার পর After delivery of placenta	2	
		মোছানো/শুকানো হয় নি Wasn't wiped / dried	3	→509
		জানি না/মনে নেই Don't know/Can't remember	97	→509
506	জন্মের কতক্ষণ পর নাম _______ কে মোছানো বা শুকানো হয়েছিল? How many minutes after delivery of (Name) he/she was wiped/dried?	মিনিট Minutes	☐☐	
		জানি না/মনে নেই Don't know/Can't remember	97	
507	কে নাম _______ এর গা মুছিয়েছিল বা শুকিয়েছিল? Who wiped (dried) the baby?	**স্বাস্থ্য পেশাজীবিঃ Health personnel:**		
		পাশ করা ডাক্তার MBBS doctor (Qualified)	01	

	নামঃ Name ___________________________<br><br>শুধুমাত্র একটি উত্তর বৃত্তায়িত করুন। **Circle only one code.**	নার্স/ ধাত্রী /প্যারামেডিক Nurse/Midwife/Paramedic	02	
		পরিবার কল্যাণ পরিদর্শিকা Family Welfare Visitor (FWV)	03	
		কমিউনিটি ভিত্তিক দক্ষ ধাত্রী (CSBA)	04	
		কমিউনিটি ক্লিনিক ভিত্তিক স্বাস্থ্য সেবা প্রদানকারী (CHCP)	05	
		চিকিৎসা সহকারী / উপসহকারী কমিউনিটি চিকিৎসা কর্মকর্তা Medical Assistant / SACMO	06	
		স্বাস্থ্য সহকারী Health Assistant (HA)	07	
		পরিবার কল্যাণ সহকারী Family Welfare Assistant (FWA)	08	
		অন্যান্য ব্যক্তিঃ **Other person:**		
		প্রশিক্ষণপ্রাপ্ত টিবিএ Trained TBA	09	
		অপ্রশিক্ষিত টিবিএ Untrained TBA	10	
		অপ্রশিক্ষিত ডাক্তার (গ্রাম ডাক্তার/ পল্লী চিকিৎসক/হোমিওপ্যাথ/ কবিরাজ) Unqualified doctor	11	
		এন জি ও স্বাস্থ্যকর্মী NGO worker	12	
		পরিবারের সদস্য Family member	13	
		আত্মীয় / প্রতিবেশী / বন্ধু Relative / Neighbour / Friend	14	
		মা নিজেই Mother herself	15	→509
		অন্যান্য (নির্দিষ্ট করুন) Other:____________________	96	
		জানি না/মনে নেই Don't know/Can't remember	97	
509	জন্মের পর কখন নাম _______ কে কাপড় দিয়ে মুড়িয়ে নেয়া হয়েছিল, ফুল পড়ার আগে না-কি ফুল পড়ার পরে?<br>After delivery when was the baby (NAME) wrapped with a cloth? Before delivery of placenta or after delivery of placenta?	ফুল পড়ার আগে Before delivery of placenta	1	
		ফুল পড়ার পর After delivery of placenta	2	
		মোছানো/শুকানো হয় নি Wasn't wiped / dried	3	→513
		জানি না/মনে নেই Don't know/Can't remember	97	→513
510	জন্মের কত মিনিট পরে নাম _______ কে কাপড় দিয়ে মুড়িয়ে নেয়া হয়েছিল?<br>How many minutes after delivery the baby was wrapped?	মিনিট Minutes	☐☐	
		জানি না/মনে নেই Don't know/Can't remember	97	
511	কে নাম _______ কে কাপড় দিয়ে মুড়িয়ে নিয়েছিল?<br>Who wrapped the baby with a cloth?<br><br>নামঃ Name ___________________________<br><br>শুধুমাত্র একটি উত্তর বৃত্তায়িত করুন।<br>Circle only one code.	স্বাস্থ্য পেশাজীবিঃ Health personnel:		
		পাশ করা ডাক্তার MBBS doctor (Qualified)	01	
		নার্স/ ধাত্রী /প্যারামেডিক Nurse/Midwife/Paramedic	02	
		পরিবার কল্যাণ পরিদর্শিকা Family Welfare Visitor (FWV)	03	
		কমিউনিটি ভিত্তিক দক্ষ ধাত্রী (CSBA)	04	
		কমিউনিটি ক্লিনিক ভিত্তিক স্বাস্থ্য সেবা প্রদানকারী (CHCP)	05	
		চিকিৎসা সহকারী / উপসহকারী কমিউনিটি চিকিৎসা কর্মকর্তা Medical Assistant / SACMO	06	
		স্বাস্থ্য সহকারী Health Assistant (HA)	07	
		পরিবার কল্যাণ সহকারী Family Welfare Assistant (FWA)	08	
		অন্যান্য ব্যক্তিঃ Other person:		
		প্রশিক্ষণপ্রাপ্ত টিবিএ Trained TBA	09	

		অপ্রশিক্ষিত টিবিএ Untrained TBA	10	
		অপ্রশিক্ষিত ডাক্তার (গ্রাম ডাক্তার/ পল্লী চিকিৎসক/হোমিওপ্যাথ/ কবিরাজ) Unqualified doctor	11	
		এন জি ও স্বাস্থ্যকর্মী NGO worker	12	
		পরিবারের সদস্য Family member	13	
		আত্মীয় / প্রতিবেশী / বন্ধু Relative / Neighbour / Friend	14	
		মা নিজেই Mother herself	15	
		অন্যান্য (নির্দিষ্ট করুন) Other:______________________	96	
		জানি না/মনে নেই Don't know/Can't remember	97	
513	নাম _______ এর নাড়ী কি দিয়ে কাটা হয়েছিল?<br>What was used to cut the cord?	ডেলিভারী কিট এর ব্লেড Blade from the delivery kit	01	
		নতুন ব্লেড New blade..	02	
		বাড়ীর পুরাতন ব্লেড Old blade in the house	03	
		বাঁশের কঞ্চি/বাতা/চল Bamboo strips	04	
		কাঁচি Scissor	05	
		অন্যান্য (নির্দিষ্ট করুন) Other: ________________________	96	
		নাড়ী কাটা হয় নি Cord was not cut	95	→526
		জানি না/মনে নেই Don't know/can't remember	97	→515
514	নাড়ী কাটার আগে **(513 এর উত্তর)** টি সিদ্ধ করে/পানিতে ফুটিয়ে নেয়া হয়েছিল কি? Was the instrument (answer of Q-513) boiled before cutting the cord?	হ্যাঁ Yes	1	
		না No	2	
		জানিনা/মনে নাই Don't know/Can't remember	97	
515	নাড়ী কাটা এবং বাঁধার পর পরই তাতে কিছু দেয়া হয়েছিল কি?<br>Was anything applied to the cord immediately after cutting and tying it?	হ্যাঁ Yes	1	
		না No	2	→518
		জানিনা/মনে নাই Don't know/Can't remember	97	→518
516	নাড়ী কাটা এবং বাঁধার পর পরই তাতে কি দেয়া হয়েছিল?<br>What was applied to the cord after cutting and tying the cord?<br><br>উত্তর পড়ে শোনাবেন না।<br>Do not read out the answers<br>জিজ্ঞেস করুনঃ আরও কিছু?<br>ASK: Anything else?<br>সব উত্তরের কোড বৃত্তায়িত করুন।<br>Circle code of all the answers	অ্যান্টিবায়োটিক (পাউডার/মলম) Antibiotics(Powder/Ointment)	A	**In all answer except the answer "C" → 526**
		ডেটল/স্যাভলন Detol/savlon	B	
		হেক্সিসল/ ক্লোরোহেক্সিডিন Chlorohexidine/Hexisol	C	
		স্পিরিট/অ্যালকোহল Spirit/Alcohol	D	
		সরিষার তেল (রসুন সহ বা বাদে) Mustard oil (with or without garlic)	E	
		চিবানো চাল Chewed rice	F	
		হলুদের রস/গুড়া Turmeric juice/powder	G	
		আদার রস Ginger juice	H	
		সিঁদুর Shidur	I	
		বরিক পাউডার Boric powder	J	
		জেনসিআন ভায়োলেট/নীল কালি Gentian violet	K	
		ট্যালকম পাউডার Talcum Powder	L	
		ছাই Ash	M	

		নারিকেল তেল Coconut oil	N	
		চুলার মাটি Dust of earth-burner	O	
		অন্যান্য (নির্দিষ্ট করুন) Other: ______________________	X	
		জানি না/মনে নেই Don't know/can't remember	Y	
517	হেক্সিকর্ড/ ক্লোরোহেক্সিডিন বোতল দেখিয়ে জিজ্ঞাসা করুনঃ এই বোতলের জিনিষ ই কি বাচ্চার নাড়ীতে লাগানো হয়েছিল?	হ্যাঁ Yes	1	
		না No	2	
		জানিনা/মনে নাই Don't know/Can't remember	97	
518	নাড়ী কাটা এবং বাঁধার ৭ দিনের মধ্যে নাড়ীতে কিছু দিয়েছিলেন কি? Was anything applied to the cord within seven days after cutting and tying it?	হ্যাঁ Yes	1	
		না No	2	→526
		বাচ্চা ৭ দিন বয়সের আগেই মারা গিয়েছে Baby died before 7 days	3	→526
		জানিনা/মনে নাই Don't know/Can't remember	97	→526
519	নাড়ী কাটা এবং বাঁধার ৭ দিনের মধ্যে নাড়ীতে কি দেয়া হয়েছিল? What was applied to the cord within seven days after cutting and tying the cord?<br><br>উত্তর পড়ে শোনাবেন না। Do not read out the answers<br>জিজ্ঞেস করুনঃ আরও কিছু? ASK: Anything else?<br>সব উত্তরের কোড বৃত্তায়িত করুন। Circle code of all the answers	অ্যান্টিবায়োটিক (পাউডার/মলম) Antibiotics(Powder/ Ointment)	A	**In all answer except the answer "C" → 526**
		ডেটল/স্যাভলন Detol/savlon	B	
		হেক্সিকর্ড/ক্লোরোহেক্সিডিন Chlorohexidine/Hexicord	C	
		স্পিরিট/অ্যালকোহল Spirit/Alcohol	D	
		সরিষার তেল (রসুন সহ বা বাদে) Mustard oil (with or without garlic)	E	
		চিবানো চাল Chewed rice	F	
		হলুদের রস/গুড়া Turmeric juice/powder	G	
		আদার রস Ginger juice	H	
		সিঁদুর Shidur	I	
		বরিক পাউডার Boric powder	J	
		জেনসিআন ভায়োলেট/নীল কালি Gentian violet	K	
		ট্যালকম পাউডার Talcum Powder	L	
		ছাই Ash	M	
		নারিকেল তেল Coconut oil	N	
		চুলার মাটি Dust of earth-burner	O	
		অন্যান্য (নির্দিষ্ট করুন) Other: ______________________	X	
		জানি না/মনে নেই Don't know/can't remember	Y	
520	ডেলিভারির সময় বা জন্মের সময় যিনি সাহায্য করেছিলেন তিনিই কি বাচ্চার নাড়িতে ক্লোরহেক্সিডিন দিয়েছিলেন? Was the person who assisted in the delivery also applied Chlorhexidine to the cord?	হ্যাঁ Yes	1	→522
		না No	2	
		জানিনা/মনে নাই Don't know/Can't remember	97	
521	কে বাচ্চার নাড়িতে ক্লোরহেক্সিডিন দিয়েছিলেন? Who applied Chlorhexidine to the cord?	<u>স্বাস্থ্য পেশাজীবিঃ Health personnel:</u>		
		পাশ করা ডাক্তার MBBS doctor (Qualified)	01	
		নার্স/ ধাত্রী /প্যারামেডিক Nurse/Midwife/Paramedic	02	
		পরিবার কল্যাণ পরিদর্শিকা Family Welfare Visitor (FWV)	03	
		কমিউনিটি ভিত্তিক দক্ষ ধাত্রী (CSBA)	04	
		কমিউনিটি ক্লিনিক ভিত্তিক স্বাস্থ্য সেবা প্রদানকারী (CHCP)	05	

		চিকিৎসা সহকারী / উপসহকারী কমিউনিটি চিকিৎসা কর্মকর্তা Medical Assistant / SACMO	06	
		স্বাস্থ্য সহকারী Health Assistant (HA)	07	
		পরিবার কল্যাণ সহকারী Family Welfare Assistant (FWA)	08	
		অন্যান্য ব্যক্তিঃ Other person:		
		প্রশিক্ষণপ্রাপ্ত টিবিএ Trained TBA	09	
		অপ্রশিক্ষিত টিবিএ Untrained TBA	10	
		অপ্রশিক্ষিত ডাক্তার (গ্রাম ডাক্তার/ পলস্নী চিকিৎসক/হোমিওপ্যাথ/ কবিরাজ) Unqualified doctor	11	
		এন জি ও স্বাস্থ্যকর্মী NGO worker	12	
		পরিবারের সদস্য Family member	13	
		আত্নীয় / প্রতিবেশী / বন্ধু Relative / Neighbour / Friend	14	
		মা নিজেই Mother herself	15	
		অন্যান্য (নির্দিষ্ট করম্নন) Other: ______________________	96	
		জানি না/মনে নেই Don't know/Can't remember	97	
522	মোট কতবার নাড়িতে ক্লোরহেক্সিডিন দেয়া হয়েছিল?	বার Times	☐☐	
		জানি না/মনে নেই Don't know/Can't remember	97	
523	বাচ্চার জন্মের কতক্ষণ পর প্রথম নাড়িতে ক্লোরহেক্সিডিন দেয়া হয়েছিল?<br>How long after birth was Chlorhexidine first applied to the cord?	জন্মের সাথে সাথেই (১ ঘন্টার মধ্যে) Just after birth (within 1 hour)	1	
		১-২৪ ঘন্টার মধ্যে 1-24 hours	2	
		২৪-৪৮ ঘন্টার মধ্যে 24-48 hours	3	
		৪৮ ঘন্টার পর After 48 hours	4	
		জানি না/মনে নাই Don't know/Can't remember	97	
524	বাচ্চার নাড়িতে যে ক্লোরহেক্সিডিন দেয়া হয়েছিল সেটি কার কাছ থেকে পেয়েছিলেন?<br>From whom you received Chlorhexidine that was applied to the cord?	স্বাস্থ্য পেশাজীবিঃ Health personnel:		
		পাশ করা ডাক্তার MBBS doctor (Qualified)	01	
		নার্স/ ধাত্রী /প্যারামেডিক Nurse/Midwife/Paramedic	02	
		পরিবার কল্যাণ পরিদর্শিকা Family Welfare Visitor (FWV)	03	
		কমিউনিটি ভিত্তিক দক্ষ ধাত্রী (CSBA)	04	
		কমিউনিটি ক্লিনিক ভিত্তিক স্বাস্থ্য সেবা প্রদানকারী (CHCP)	05	
		চিকিৎসা সহকারী / উপসহকারী কমিউনিটি চিকিৎসা কর্মকর্তা Medical Assistant / SACMO	06	
		স্বাস্থ্য সহকারী Health Assistant (HA)	07	
		পরিবার কল্যাণ সহকারী Family Welfare Assistant (FWA)	08	
		অন্যান্য ব্যক্তিঃ Other person:		
		প্রশিক্ষণপ্রাপ্ত টিবিএ Trained TBA	09	
		অপ্রশিক্ষিত টিবিএ Untrained TBA	10	
		অপ্রশিক্ষিত ডাক্তার (গ্রাম ডাক্তার/ পলস্নী চিকিৎসক/হোমিওপ্যাথ/ কবিরাজ) Unqualified doctor	11	
		এন জি ও স্বাস্থ্যকর্মী NGO worker	12	
		পরিবারের সদস্য Family member	13	
		আত্নীয় / প্রতিবেশী / বন্ধু Relative / Neighbour / Friend	14	

		ওষুধ বিক্রেতা Drug seller	15	
		অন্যান্য (নির্দিষ্ট করুন) Other: ______________________	96	
		জানি না/মনে নেই Don't know/Can't remember	97	
525	বাচ্চার নাড়িতে যে ক্লোরহেক্সিডিন দেয়া হয়েছিল সেটি কোথা থেকে পেয়েছিলেন?<br>From where you received Chlorhexidine that was applied to the cord?	সরকারী সেকটর **Public sector:**		
		জেলা হাসপাতাল District hospital	11	
		মাতৃমঙ্গল কেন্দ্র Maternal & Child Welfare Centre (MCWC)	12	
		উপজেলা স্বাস্থ্য কমপ্লেক্স Upazila Health Complex	13	
		পরিবার কল্যাণ কেন্দ্র Family Welfare Centre (FWC)	14	
		স্যাটেলাইট ক্লিনিক/ইপিআই কেন্দ্র Satellite clinic/EPI centre	15	
		কমিউনিটি ক্লিনিক Community clinic	16	
		এন জি ও সেকটর **NGO sector:**		
		এন জি ও স্থায়ী ক্লিনিক NGO static clinic	21	
		এন জি ও স্যাটেলাইট ক্লিনিক NGO satellite clinic	22	
		প্রাইভেট সেকটর **Private sector:**		
		প্রাইভেট হাসপাতাল/ক্লিনিক Private hospital/Clinic	31	
		ডাক্তারের চেম্বার Chamber of doctor	32	
		ফার্মেসী/ওষুধের দোকান Pharmacy	33	
		অন্যান্য(নির্দিষ্ট করুন) Other:	96	
526	জন্মের পর পরই (নাম) স্বাভাবিকভাবে কেঁদেছিল বা শ্বাস নিয়েছিল কি?<br>Did your baby cry/ breathe normally immediately after birth?	হ্যাঁ Yes	1	→529
		না No	2	
		জানিনা/মনে নাই Don't know/Can't remember	97	
527	জন্মের পর পরই (নাম) _____ কে কাঁদানোর জন্য বা শ্বাস নেয়ানোর জন্য কিছু করতে হয়েছিল কি?<br>Was anything done to help the baby cry or breath immediately after birth?<br><br>উত্তর পড়ে শোনাবেন না।<br>Do not read out the answers<br>জিজ্ঞেস করুনঃ আরও কিছু?<br>ASK: Anything else?<br>সব উত্তরের কোড বৃত্তায়িত করুন।<br>Circle code of all the answers	কোন উত্তর দেয় নি Didn't give any answer	A	→529
		বাচ্চার গা শুকানো হয়েছে Dried the baby	B	
		বাচ্চাকে মুড়িয়ে নেয়া হয়েছে Wrapped the baby	C	
		বাচ্চার পিঠে ঘষা দিয়ে উত্তেজিত করা হয়েছে Rubbed the back for stimulation	D	
		বাচ্চার পায়ের পাতা ঘষা দিয়ে উত্তেজিত করা হয়েছে Rubbed the feet for stimulation	E	
		মুখ থেকে মুখে শ্বাস নেয়ানোর চেষ্টা করা হয়েছে Mouth to mouth respiration	F	
		নাড়ীতে তাপ দেয়া হয়েছে Heated the cord	G	
		বাচ্চাকে থাপ্পড় দেয়া হয়েছে Slapped the baby	H	
		বাচ্চার মাথা নিচ দিকে দিয়ে ঝুলানো হয়েছে Hold the baby upside down	I	
		ব্যাগ/মাস্ক দিয়ে শ্বাস দেয়া হয়েছে Used bag and mask	J	
		অন্যান্য (নির্দিষ্ট করুন) Other: ______________________	X	
		জানি না/মনে নেই Don't know/can't remember	Y	→529
		কিছুই করা হয় নি Nothing done	Z	→529

528	কে (নাম) ______ কে কাঁদানোর বা শ্বাস নেয়ানোর জন্য চেষ্টা বা কিছু করেছিলেন?<br>Who took initiative to resuscitate or to help the baby cry?<br><br>Circle only one code.<br>শুধুমাত্র একটি কোড বৃত্তায়িত করুন।	স্বাস্থ্য পেশাজীবিঃ Health personnel:		
		পাশ করা ডাক্তার MBBS doctor (Qualified)	01	
		নার্স/ ধাত্রী /প্যারামেডিক Nurse/Midwife/Paramedic	02	
		পরিবার কল্যাণ পরিদর্শিকা Family Welfare Visitor (FWV)	03	
		কমিউনিটি ভিত্তিক দক্ষ ধাত্রী (CSBA)	04	
		কমিউনিটি ক্লিনিক ভিত্তিক স্বাস্থ্য সেবা প্রদানকারী (CHCP)	05	
		চিকিৎসা সহকারী / উপসহকারী কমিউনিটি চিকিৎসা কর্মকর্তা Medical Assistant / SACMO	06	
		স্বাস্থ্য সহকারী Health Assistant (HA)	07	
		পরিবার কল্যাণ সহকারী Family Welfare Assistant (FWA)	08	
		অন্যান্য ব্যক্তিঃ Other person:		
		প্রশিক্ষণপ্রাপ্ত টিবিএ Trained TBA	09	
		অপ্রশিক্ষিত টিবিএ Untrained TBA	10	
		অপ্রশিক্ষিত ডাক্তার (গ্রাম ডাক্তার/ পলস্নী চিকিৎসক/হোমিওপ্যাথ/ কবিরাজ) Unqualified doctor	11	
		এন জি ও স্বাস্থ্যকর্মী NGO worker	12	
		পরিবারের সদস্য Family member	13	
		আত্মীয় / প্রতিবেশী / বন্ধু Relative / Neighbour / Friend	14	
		মা নিজেই Mother herself	15	
		অন্যান্য (নির্দিষ্ট করুন) Other: ___________________	96	
		জানি না/মনে নেই Don't know/can't remember	97	
529	বাচ্চা (নাম) কে জন্মের পর কখন প্রথম গোসল করানো হয়েছিল?<br>When was (NAME) given a bath for the first time?<br>জন্মের 1 ঘন্টার মধ্যে হলে '000' বৃত্তায়িত করুন।<br>24 ঘন্টার মধ্যে হলে '1' বৃত্তায়িত করে বক্সে সঠিক ঘন্টা লিখুন।<br>24 ঘন্টা বা তার অধিক হলে কোড '2' বৃত্তায়িত করে উত্তর দিনে লিখুন।<br>If before/within an hour circle "000", If before/ within 24 hours then circle 1 and write in the space for hour, If 24 hours/after 24 hours then circle 2 and write in the space for days.	সাথে সাথেই Immediately	000	
		ঘন্টা Hours ☐☐	ঘন্টা পর	
		দিন Days ☐☐	দিন পর	
		গোসল করানো হয় নাই Did not give bath	996	
		জানি না/মনে নেই Don't know/can't remember	997	
530	বাচ্চা (নাম) কে কখনও বুকের দুধ খাইয়েছিলেন কি?<br>Have you ever breastfed (NAME)?	হ্যাঁ Yes	1	
		না No	2	→534
531	ফুল পড়া বা বের হওয়ার আগেই কি বাচ্চা (নাম) কে বুকের দুধ খাওয়ানো হয়েছিল?<br>Was the baby put to breast before delivery of the placenta?	হ্যাঁ Yes	1	
		না No	2	
		জানিনা/মনে নাই Don't know/Can't remember	97	
532	জন্মের কত সময় পর বাচ্চা (নাম) কে প্রথম বুকের দুধ খাওয়ানো হয়েছিল?	জন্মের পর পর Immediately after birth	00	
		জন্মের ☐☐ ঘন্টার মধ্যে How many hours after birth	1	

	When was the baby breast-fed for the first time after birth?<br>জন্মের 1 ঘন্টার মধ্যে হলে '000' বৃত্তায়িত করুন।<br>24 ঘন্টার মধ্যে হলে '1' বৃত্তায়িত করে বক্সে সঠিক ঘন্টা লিখুন।<br>24 ঘন্টা বা তার অধিক হলে কোড '2' বৃত্তায়িত করে উত্তর দিনে লিখুন।<br>If before/within an hour circle "000", If before/ within 24 hours then circle 1 and write in the space for hour, If 24 hours/after 24 hours then circle 2 and write in the space for days.	জন্মের ☐☐ দিন পর How many days after birth	2	
533	জন্মের সাথে সাথে (নাম) ______ কে শাল দুধ খাওয়ানো হয়েছিল কি? Was (NAME) given colostrum immediately after his/her birth?	হ্যাঁ Yes	1	
		না No	2	
534	বুকের দুধ খাওয়ানোর পূর্বে অথবা জন্মের পর প্রথম তিন দিন (নাম) ______ কে অন্য কোন খাবার বা পানীয় খাওয়ানো হয়েছিল কি?<br>Before giving breastfeeding or in the first three days after delivery was (NAME) given any other food or liquid?	হ্যাঁ Yes	1	
		না No	2	→536
		জানিনা/মনে নাই Don't know/Can't remember	97	→536
535	নাম _______ এর জন্মের পর প্রথম তিন দিনের মধ্যে যে কোন সময় (বুকের দুধ ছাড়া অন্য) কি কি খাবার বা পানীয় খাওয়ানো হয়েছিল?<br>What other food or liquid was given besides breast milk at any point during first 3 days?<br>জিজ্ঞেস করুনঃ আরও কিছু খাইয়েছিলেন কি?<br>Ask: Did you feed any other things?<br>সব উত্তরের কোড বৃত্তায়িত করুন।<br>Write down all the answers.	মধু Honey	A	
		মিস্রীর পানি Misri water	B	
		চিনির পানি Sugar water	C	
		পানি Water	D	
		ফলের রস Fruit juice	E	
		টিনজাত দুধ/শিশু খাদ্য (বেবী ফর্মুলা) Tinned milk/ infant formula from shop	F	
		গরুর দুধ Cow's milk	G	
		অন্যান্য তরল Other liquids	H	
		লেই (পানিতে মিশানো চালের গুড়া, আটা, ময়দা) Powdered rice with water (Lei)	I	
		কলা/পেঁপে/আম Banana / Papaya/ Mango	J	
		সবুজ শাকসব্জী Green leafy vegetable	K	
		ভাত/রুটি Rice/bread	L	
		ডাল Lentil/pulse/dal	M	
		অন্যান্য (নির্দিষ্ট করুন) Other: ________________________	X	
536	জন্মের পর পর নাম_______এর শরীর গরম রাখার জন্য কি করেছিলেন?<br>What have you done to keep (NAME) warm following delivery<br>উত্তর পড়ে শোনাবেন না।<br>Do not read out the answers<br>জিজ্ঞেস করুনঃ আরও কিছু?<br>ASK: Anything else?	শুকিয়েছিলাম Dried the baby	A	
		পরিস্কার কাপড়/কাঁথা দিয়ে মুড়িয়ে নিয়েছিলাম Wrapped the baby with clean cloths/'katha'	B	
		বুকের চামড়ার উপরে বাচ্চাকে চেপে রেখেছিলাম Kept the baby on bare skin to skin contact	C	
		রান্নাঘরে ডেলিভারি করানো হয়েছিল Conducted delivery at kitchen	D	
		ডেলিভারি কক্ষে আগুন জ্বালিয়ে রাখার ব্যবস্থা করা হয়েছিল Lighted some fire at delivery room	E	

	সব উত্তরের কোড বৃত্তায়িত করুন। Circle code of all the answers	গরম তেল শরীরে মালিশ করেছিলাম Rubbed the baby with hot oil	F	
		আমার কোলে বাচ্চাকে রেখেছিলাম Kept newborn on my lap	G	
		অন্য কারো কোলে বাচ্চাকে রাখা হয়েছিল Kept newborn at others lap	H	
		অন্যান্য (নির্দিষ্ট করুন) Other ________________________	X	
		কিছুই করিনি Nothing done	Z	

নবজাতক স্বাস্থ্য জটিলতায় চিকিৎসা বাবদ খরচ সমুহ Teatment cost for neonatal complication

এখন অমি আপনার বাচ্চার স্বাস্থ্য সমস্যা সম্পর্কে আলোচনা করব। Now I would like to discuss about the health of your child

No.	Questions and filters	Responses	Code	Skip
537	(নাম) _____এর জন্মের ১ মাসের মধ্যে তাঁর কি কোনো স্বাস্থ্যসমস্যা বা জটিলতা হয়েছিল? Now I will discuss about the health problems of your baby. After the birth of (NAME) did s/he suffer from any sort problem/complication within first month of life?	হ্যাঁ Yes	1	
		না No	2	→end this section
		জানিনা/মনে নাই Don't know/Can't remember	97	→end this section
538	আপনার (নাম) _____এর কি ধরণের সমস্যা বা জটিলতা হয়েছিল? Please tell me what were the problem/ complication with your baby (NAME)? উত্তর পড়ে শোনাবেন না। Do not read out the answers জিজ্ঞেস করুনঃ আরও কিছু? ASK: Anything else? সব উত্তরের কোড বৃত্তায়িত করুন। Circle code of all the answers	কষ্টকর/দ্রুত শ্বাস নেয়া Difficult or fast breathing	A	
		নিউমোনিয়া Pneumonia	B	
		ঠান্ডা/কফ/সর্দি/কাশি Cold/cough	C	
		চামড়ার রং, হাতের তালু, পায়ের পাতা, চোখ হলুদ হওয়া (জন্ডিস) Yellow skin/palm/feet/eye color (jaundice)	D	
		বাচ্চার খাওয়া কমে যাওয়া/বুকের দুধ চুষতে না পারা Poor sucking or feeding	E	
		নাভি থেকে পুঁজ, রক্ত বা অন্যকিছু বের হওয়া Pus, bleeding, or discharge from around the umbilical cord	F	
		চামড়ায় ফোসকা/ঘা হওয়া Skin lesions or blisters	G	
		খিঁচুনী/শরীর শক্ত হয়ে যাওয়া Convulsions/spasms/rigidity	H	
		অচেতন/অজ্ঞান/হুঁশ না থাকা Lethargy/unconsciousness	I	
		চোখ লাল হওয়া/ফুলে যাওয়া/চোখ থেকে পুঁজ বা পিঙ্গিস বের হওয়া Red or swollen eyes with pus	J	
		বাচ্চার শরীর ঠান্ডা হওয়া Baby feels cold	K	
		বাচ্চা না কাঁদা Baby doesn't cry	L	
		জ্বর Fever	M	
		প্রস্রাব না হওয়া Doesn't pass urine	N	
		পায়খানা না করা Doesn't pass stool	O	

		একটানা বমি Continuous vomiting	P	
		পেট ফোলা/ফাঁপা Distended abdomen	Q	
		ঘুম থেকে জাগানো কষ্টকর Difficult to wake	R	
		চামড়ায় ফুসকুড়ি/র‍্যাশ/মাসিপিসি Skin rash/'Mashipishi'...	S	
		হাম Measles	T	
		ডায়রিয়া Diarrhoea	U	
		বুকের খাঁচা ডেবে যাওয়া বা ভিতরে ঢুকে যাওয়া Chest in-drawing	V	
		অন্যান্য (নির্দিষ্ট করুন) Other: ____________________	X	
539	(নাম) _____এর এই সমস্যা/অসুবিধা/জটিলতার জন্য আপনি কোন চিকিৎসা করিয়েছেন কি?<br>Did you seek any sort of treatment for this problem/complication with (NAME)?	হ্যাঁ Yes	1	
		না No	2	→end this section
		জানিনা/মনে নাই Don't know/Can't remember	97	→end this section
540	আপনি কার কাছে চিকিৎসা করিয়েছেন?<br>From whom you received treatment for this problem/complication?<br><br>জিজ্ঞেস করুনঃ আর কেউ?<br>Anyone else?<br><br>ব্যক্তি সর্ম্পকে নিশ্চিত হোন এবং সঠিক উত্তরের কোড বৃত্তায়িত করুন।<br>Probe to identify each type of person and check all mentioned.	স্বাস্থ্য পেশাজীবিঃ Health personnel:		
		পাশ করা ডাক্তার MBBS doctor (Qualified)	A	
		নার্স/ ধাত্রী /প্যারামেডিক Nurse/Midwife/Paramedic	B	
		পরিবার কল্যাণ পরিদর্শিকা Family Welfare Visitor (FWV)	C	
		চিকিৎসা সহকারী / উপসহকারী কমিউনিটি চিকিৎসা কর্মকর্তা Medical Assistant / SACMO	D	
		কমিউনিটি ভিত্তিক দক্ষ ধাত্রী (CSBA)	E	
		কমিউনিটি ক্লিনিক ভিত্তিক স্বাস্থ্য সেবা প্রদানকারী (CHCP)	F	
		স্বাস্থ্য সহকারী Health Assistant (HA)	G	
		পরিবার কল্যাণ সহকারী Family Welfare Assistant (FWA)	H	
		অন্যান্য ব্যক্তিঃ Other person:		
		প্রশিক্ষণপ্রাপ্ত টিবিএ Trained TBA	I	
		অপ্রশিক্ষিত টিবিএ Untrained TBA	J	
		অপ্রশিক্ষিত ডাক্তার (গ্রাম ডাক্তার/ পলস্নী চিকিৎসক/হোমিওপ্যাথ/ কবিরাজ) Unqualified doctor	K	
		এন জি ও স্বাস্থ্যকর্মী NGO worker	L	
		পরিবারের সদস্য/আত্মীয় Family member / Relative	M	
		প্রতিবেশী/বন্ধু Neighbour / Friend	N	
		অন্যান্য (নির্দিষ্ট করুন) Other: ___________________	X	
		জানিনা/মনে নাই Don't know/Can't remember	Y	
541	এই চিকিৎসা আপনি কোথায় করিয়েছেন?<br>From where did you receive care for this problem/complication?<br><br>স্থান সর্ম্পকে নিশ্চিত হোন এবং সঠিক উত্তরের কোড বৃত্তায়িত করুন।<br>Probe to identify the type of source and circle the appropriate code.	বাড়ি Home:		
		বাড়িতে Home	A	
		সরকারী সেক্টর Public sector:		
		বিশেষায়িত হাসপাতাল / মেডিকেল কলেজ হাসপাতাল Specialized hospital/Medical College	B	
		জেলা হাসপাতাল District hospital	C	
		মাতৃমঙ্গল কেন্দ্র Maternal & Child Welfare Centre (MCWC)	D	
		উপজেলা স্বাস্থ্য কমপ্লেক্স Upazila Health Complex	E	
		পরিবার কল্যাণ কেন্দ্র Family Welfare Centre (FWC)	F	

	সরকারি না প্রাইভেট সেকটর নিশ্চিত হতে না পারলে, নীচে স্থানটির নাম লিখুনঃ<br>If unable to determine if public or private sector, write the name of the place:<br><br>___________________________________<br>স্থানের নাম (Name of place)	স্যাটেলাইট ক্লিনিক/ইপিআই কেন্দ্র Satellite clinic/EPI centre	G	
		কমিউনিটি ক্লিনিক Community clinic	H	
		অন্যান্য (নির্দিষ্ট করুন) Other: ____________________	I	
		<u>এন জি ও সেকটর NGO sector:</u>		
		এন জি ও ক্লিনিক NGO static clinic	J	
		এন জি ও স্যাটেলাইট ক্লিনিক NGO satellite clinic	K	
		অন্যান্য (নির্দিষ্ট করুন) Other: ____________________	L	
		<u>প্রাইভেট সেকটর Private medical sector:</u>		
		প্রাইভেট হাসপাতাল / ক্লিনিক Private hospital/Clinic	M	
		পাশ করা ডাক্তার MBBS doctor (Qualified)	N	
		অপ্রশিক্ষিত ডাক্তার Unqualified doctor	O	
		ফার্মেসী Pharmacy	P	
		অন্যান্য (নির্দিষ্ট করুন) Other: ____________________	X	
542	আপনার বাচ্চার চিকিৎসাকালীন কি কোন খরচ হয়েছিল?<br>Did you incur any cost for the treatment or care of your child?	হ্যাঁ Yes	1	
		না No	2	→end this section

এখন আমি আপনার কাছে সেবা গ্রহন বাবদ খরচ সমূহ সম্পর্কে বিস্তারিত জানতে চাইব। (ভিজিট অনুযায়ী উল্লেখ করুন) I will now ask you details about the costs that you had incurred for care seeking (Please mention by visit).

543	ডাক্তার বা সেবাপ্রদানকারীর ফি বাবদ কোন খরচ হয়েছিল কি? হ্যাঁ হলে, ডাক্তার বা সেবাপ্রদানকারীর ফি বাবদ মোট কত টাকা খরচ হয়েছিল?<br>Did you incur any cost for consulting a Doctor or a Service provider? How much did you pay to the doctor or service provider as fees?	১ম ভিজিট<br>1st visit	২য় ভিজিট<br>2nd visit	৩য় ভিজিট<br>3rd visit	৪র্থ ভিজিট<br>4th visit	
		হ্যাঁ Yes .... 1<br>না No .... 2<br>Tk.__________	হ্যাঁ Yes.........1<br>না No...........2<br>Tk.__________	হ্যাঁ Yes .... 1<br>না No .... 2<br>Tk.__________	হ্যাঁ Yes.........1<br>না No ....2<br>Tk.__________	
544	নাম ________ কে হাসপাতালে ভর্তি হতে হয়েছিল? কতদিন হাসপাতালে থাকতে হয়েছিল? বেড/কেবিন ভাড়া বাবদ কত খরচ হয়েছিল?<br>Were you admitted at the hospital? How long did you have to stay at the hospital? How much Did you pay for the bed/cabin?	১ম ভিজিট<br>1st visit	২য় ভিজিট<br>2nd visit	৩য় ভিজিট<br>3rd visit	৪র্থ ভিজিট<br>4th visit	
		হ্যাঁ Yes .... 1<br>না No .... 2<br>হ্যাঁ হলে, হাসপাতালে কতদিন ছিলেন?<br>Days of hospital stay |___|___| Tk.__________	হ্যাঁ Yes.........1<br>না No...........2<br>হ্যাঁ হলে, হাসপাতালে কতদিন ছিলেন?<br>Days of hospital stay |___|___| Tk.__________	হ্যাঁ Yes .... 1<br>না No .... 2<br>হ্যাঁ হলে, হাসপাতালে কতদিন ছিলেন?<br>Days of hospital stay |___|___| Tk.__________	হ্যাঁ Yes.........1<br>না No ....2<br>হ্যাঁ হলে, হাসপাতালে কতদিন ছিলেন?<br>Days of hospital stay |___|___| Tk.__________	
545	নাম ________ এর কি কোন অপারেশন হয়েছিল? অপারেশন বাবদ কত খরচ হয়েছিল?<br>Did you undergo any surgery? How much did you have to pay for operation?	১ম ভিজিট<br>1st visit	২য় ভিজিট<br>2nd visit	৩য় ভিজিট<br>3rd visit	৪র্থ ভিজিট<br>4th visit	
		হ্যাঁ Yes .... 1<br>না No .... 2<br>হ্যাঁ হলে, কত খরচ হয়েছিল?<br>Tk.__________	হ্যাঁ Yes.........1<br>না No...........2<br>হ্যাঁ হলে, কত খরচ হয়েছিল?<br>Tk.__________	হ্যাঁ Yes .... 1<br>না No .... 2<br>হ্যাঁ হলে, কত খরচ হয়েছিল?<br>Tk.__________	হ্যাঁ Yes.........1<br>না No ....2<br>হ্যাঁ হলে, কত খরচ হয়েছিল?<br>Tk.__________	
546	নাম ________ কে কি কোন ঔষধ /ইঞ্জেকশান/ স্যালাইন খেতে হয়েছিল?<br>এসব ক্রয় বাবদ কত খরচ হয়েছিল?<br>Did the child take any drugs/ medicine/ injection/ saline? How much did you pay	১ম ভিজিট<br>1st visit	২য় ভিজিট<br>2nd visit	৩য় ভিজিট<br>3rd visit	৪র্থ ভিজিট<br>4th visit	
		হ্যাঁ Yes .... 1<br>না No .... 2<br>হ্যাঁ হলে, কত খরচ হয়েছিল?	হ্যাঁ Yes.........1<br>না No...........2<br>হ্যাঁ হলে, কত খরচ হয়েছিল?	হ্যাঁ Yes .... 1<br>না No .... 2<br>হ্যাঁ হলে, কত খরচ হয়েছিল?	হ্যাঁ Yes.........1<br>না No ....2<br>হ্যাঁ হলে, কত খরচ হয়েছিল?	

	for buying them?	Tk.__________	Tk.__________	Tk.__________	Tk.__________	
547	হাসপাতালে যাওয়ার অথবা ফিরে আসার পথে যাতায়াত বাবদ কোন খরচ হয়েছিল কি? যাতায়াত বাবদ মোট কত খরচ হয়েছিল?<br>Did you incur any transport cost while travelling to and returning from the provider? How much did you pay for transport cost in total?	১ম ভিজিট<br>1st visit	২য় ভিজিট<br>2nd visit	৩য় ভিজিট<br>3rd visit	৪র্থ ভিজিট<br>4th visit	
		হ্যাঁ Yes .... 1<br>না No .... 2<br><br>হ্যাঁ হলে, কত খরচ হয়েছিল?<br>Tk.__________	হ্যাঁ Yes..........1<br>না No...........2<br><br>হ্যাঁ হলে, কত খরচ হয়েছিল?<br>Tk.__________	হ্যাঁ Yes .... 1<br>না No .... 2<br><br>হ্যাঁ হলে, কত খরচ হয়েছিল?<br>Tk.__________	হ্যাঁ Yes..........1<br>না No ....2<br><br>হ্যাঁ হলে, কত খরচ হয়েছিল?<br>Tk.__________	
548	এই সেবাগ্রহণ করার সময়ে খাবার অথবা পানীয় কিনে খেতে কোন খরচ হয়েছিল কি? খাবার অথবা পানীয় কিনে খেতে কত খরচ হয়েছিল?<br>Did you incur any cost on food or beverages? How much did you pay for food or beverages?	১ম ভিজিট<br>1st visit	২য় ভিজিট<br>2nd visit	৩য় ভিজিট<br>3rd visit	৪র্থ ভিজিট<br>4th visit	
		হ্যাঁ Yes .... 1<br>না No .... 2<br><br>হ্যাঁ হলে, কত খরচ হয়েছিল?<br>Tk.__________	হ্যাঁ Yes..........1<br>না No...........2<br><br>হ্যাঁ হলে, কত খরচ হয়েছিল?<br>Tk.__________	হ্যাঁ Yes .... 1<br>না No .... 2<br><br>হ্যাঁ হলে, কত খরচ হয়েছিল?<br>Tk.__________	হ্যাঁ Yes..........1<br>না No ....2<br><br>হ্যাঁ হলে, কত খরচ হয়েছিল?<br>Tk.__________	
549	চিকিৎসা সেবা নিতে যাওয়ার পথে অথবা সেখান থেকে ফিরে আসার পথে আপনাকে হোটেল বা অন্যকোথাও রাত কাটাতে হয়েছিল কি? হোটেল বা অন্যকোথাও থাকা বাবদ কত খরচ হয়েছিল?<br>Did you spend night away from home either while going there or while coming back? Did you incur any cost for staying over? How much did you pay?	১ম ভিজিট<br>1st visit	২য় ভিজিট<br>2nd visit	৩য় ভিজিট<br>3rd visit	৪র্থ ভিজিট<br>4th visit	
		হ্যাঁ Yes .... 1<br>না No .... 2<br>হ্যাঁ হলে, কত খরচ হয়েছিল?<br><br>Tk.__________	হ্যাঁ Yes..........1<br>না No...........2<br>হ্যাঁ হলে, কত খরচ হয়েছিল?<br><br>Tk.__________	হ্যাঁ Yes .... 1<br>না No .... 2<br>হ্যাঁ হলে, কত খরচ হয়েছিল?<br><br>Tk.__________	হ্যাঁ Yes..........1<br>না No ....2<br>হ্যাঁ হলে, কত খরচ হয়েছিল?<br><br>Tk.__________	
550	নাম ________ কে রক্ত/ মল/ মুত্র/ এক্সরে /আল্ট্রাসনোগ্রাম পরীক্ষা করাতে হয়েছিল কি? এসব পরীক্ষা করাতে কত খরচ হয়েছিল?<br>Did you spend any money for blood/stool/urine test?<br>How much did you spend for each of these tests?<br><br>যদি উত্তর না জানা থাকে তবে 'DK' লিখুন<br>If not known then write'DK'	১ম ভিজিট<br>1st visit	২য় ভিজিট<br>2nd visit	৩য় ভিজিট<br>3rd visit	৪র্থ ভিজিট<br>4th visit	
		হ্যাঁ Yes .... 1<br>না No .... 2<br><br>১। রক্ত পরীক্ষা<br>Blood test<br>Tk.__________<br><br>২। মুত্র পরীক্ষা<br>Urine test<br>Tk.__________<br><br>৩। মল পরীক্ষা<br>Stool test<br>Tk.__________<br><br>৪। আল্ট্রাসনোগ্রাম<br>Ultra sonogram<br>Tk.__________	হ্যাঁ Yes..........1<br>না No...........2<br><br>১। রক্ত পরীক্ষা<br>Blood test<br>Tk.__________<br><br>২। মুত্র পরীক্ষা<br>Urine test<br>Tk.__________<br><br>৩। মল পরীক্ষা<br>Stool test<br>Tk.__________<br><br>৪। আল্ট্রাসনোগ্রাম<br>Ultra sonogram<br>Tk.__________	হ্যাঁ Yes .... 1<br>না No .... 2<br><br>১। রক্ত পরীক্ষা<br>Blood test<br>Tk.__________<br><br>২। মুত্র পরীক্ষা<br>Urine test<br>Tk.__________<br><br>৩। মল পরীক্ষা<br>Stool test<br>Tk.__________<br><br>৪। আল্ট্রাসনোগ্রাম<br>Ultra sonogram<br>Tk.__________	হ্যাঁ Yes..........1<br>না No ....2<br><br>১। রক্ত পরীক্ষা<br>Blood test<br>Tk.__________<br><br>২। মুত্র পরীক্ষা<br>Urine test<br>Tk.__________<br><br>৩। মল পরীক্ষা<br>Stool test<br>Tk.__________<br><br>৪। আল্ট্রাসনোগ্রাম<br>Ultra sonogram<br>Tk.__________	
551	নাম ________ কে কি স্যালাইন/ রক্ত/অক্সিজেন দিতে হয়েছিল? এগুলো বাবদ কত খরচ হয়েছিল?<br>Did you spend any money for saline infusion or blood transfusion or oxygen inhalation during your stay at health facility? How much did you sopned for each of these?	১ম ভিজিট<br>1st visit	২য় ভিজিট<br>2nd visit	৩য় ভিজিট<br>3rd visit	৪র্থ ভিজিট<br>4th visit	
		হ্যাঁ Yes .... 1<br>না No .... 2<br><br>১। স্যালাইন Saline<br>Tk.__________	হ্যাঁ Yes..........1<br>না No...........2<br><br>১। । স্যালাইন<br>Saline<br>Tk.__________	হ্যাঁ Yes .... 1<br>না No .... 2<br><br>১। স্যালাইন Saline<br>Tk.__________	হ্যাঁ Yes..........1<br>না No ....2<br><br>১। । স্যালাইন Saline<br>Tk.__________	

	যদি উত্তর না জানা থাকে তবে 'DK' লিখুন<br>If not known then write'DK'	২। রক্ত<br>Blood transfusion<br>Tk.__________<br>৩। অক্সিজেন Oxygen<br>Tk.__________	২। রক্ত<br>Blood transfusion<br>Tk.__________<br>৩। অক্সিজেন Oxygen<br>Tk.__________	২। রক্ত<br>Blood transfusion<br>Tk.__________<br>৩। অক্সিজেন Oxygen<br>Tk.__________	২। রক্ত<br>Blood transfusion<br>Tk.__________<br>৩। অক্সিজেন Oxygen<br>Tk.__________	
552	ওয়ার্ড বয়, এটেনডেন্ট (সাহায্যকারী)/ দাড়োয়ানদের উপহার/বকশিশ বাবদ কোন খরচ হয়েছিল কি? কত খরচ হয়েছিল?<br>Did you pay for tips to ward boy, attendant, gate keeper? How much cost did you incur?	১ম ভিজিট<br>1st visit<br>হ্যাঁ Yes .... 1<br>না No .... 2<br>হ্যাঁ হলে, কত খরচ হয়েছিল?<br>Tk.__________	২য় ভিজিট<br>2nd visit<br>হ্যাঁ Yes..........1<br>না No...........2<br>হ্যাঁ হলে, কত খরচ হয়েছিল?<br>Tk.__________	৩য় ভিজিট<br>3rd visit<br>হ্যাঁ Yes .... 1<br>না No .... 2<br>হ্যাঁ হলে, কত খরচ হয়েছিল?<br>Tk.__________	৪র্থ ভিজিট<br>4th visit<br>হ্যাঁ Yes..........1<br>না No ....2<br>হ্যাঁ হলে, কত খরচ হয়েছিল?<br>Tk.__________	
553	আপনার কোন সাহায্যকারী ছিল কি?<br>Did you have any attendant?	হ্যাঁ Yes .... 1<br>না No .... 2→	হ্যাঁ Yes..........1<br>না No...........2→	হ্যাঁ Yes .... 1<br>না No .... 2→	হ্যাঁ Yes..........1<br>না No ....2→	If 2→ 555
554	আপনার এটেনডেন্ট (সাহায্যকারী) আপনাকে সঙ্গ দেওয়ার সময়কালীন কোন অর্থনৈতিক ড়্গতির সম্মুখিন হয়েছিল? (অর্থাৎ সাহায্যকারী কোন কাজ করে এবং আপনার সাথে থাকার কারনে সে পারিশ্রমিক থেকে বঞ্চিত) হয়ে থাকলে তার পরিমাণ?<br>Did your attendant lose any income as a result of time spent with you on seeking care? If yes, how much did he/she lose?	১ম ভিজিট<br>1st visit<br>হ্যাঁ Yes .... 1<br>না No .... 2<br>হ্যাঁ হলে, কত টাকা থেকে বঞ্চিত হয়েছিল?<br>Tk.__________	২য় ভিজিট<br>2nd visit<br>হ্যাঁ Yes..........1<br>না No...........2<br>হ্যাঁ হলে, কত টাকা থেকে বঞ্চিত হয়েছিল?<br>Tk.__________	৩য় ভিজিট<br>3rd visit<br>হ্যাঁ Yes .... 1<br>না No .... 2<br>হ্যাঁ হলে, কত টাকা থেকে বঞ্চিত হয়েছিল?<br>Tk.__________	৪র্থ ভিজিট<br>4th visit<br>হ্যাঁ Yes..........1<br>না No ....2<br>হ্যাঁ হলে, কত টাকা থেকে বঞ্চিত হয়েছিল?<br>Tk.__________	

555	আপনার এই চিকিৎসাকালীন সময়ে যে সকল খরচ হয়েছিল, তা কিভাবে মিটিয়েছিলেন?<br>(একাধিক উত্তর গ্রহণযোগ্য)<br>How did you meet the expenditure that was incurred due to careseeking for your treatment?<br>(multiple answers acceptable)	সুদ সহ ঋণ এর মাধ্যমে/ধার করে Loan with Interest	A	→for any option other than A &B go to next section
		সুদ বিহীন ঋণ এর মাধ্যমে/ধার করে Loan without interest	B	
		সঞ্চয় থেকে বা বাড়ীর অন্যান্য খরচ বাচিয়ে Savings after all household expenses	C	
		সম্পত্তি বিক্রয় করে Selling land and assets	D	
		আত্মীয় / বন্ধু এর কাছ থেকে সাহায্য হিসাবে Help from Relatives/ Friends	E	
		নিয়মিত আয় থেকে Regular Income	F	
		নিজস্ব জিনিস বিক্রি করে Sold Personal Belongings	G	
		গৃহ পালিত পশু বিক্রি করে Sold Livestock.	H	
		গাছ অথবা উৎপাদিত শস্য বিক্রি কওে Sold agricultural product/tree	I	
		স্থায়ী সম্পদ বিক্রি করে Sold permanent assets	J	
		জমি বা সম্পদ জামানত রেখে Mortgage of Assets/land	K	
		অন্যান্য, উল্লেখ করচন Others Specify ________________________________	X	
556	আপনি কোথা থেকে ধার করেছিলেন/ঋণ নিয়েছিলেন?<br>Where did you take the loan from?<br>(একাধিক উত্তর গ্রহণযোগ্য)<br>(multiple answers acceptable)	মাইক্রোক্রেডিট সংস্থা (এন জি ও) Microcredit (NGO) ব্যাংক Bank	A	
		গ্রামের মহাজন Village Mohajon	B	
		আত্মীয় Relative	C	
		প্রতিবেশী Neighbour	D	
		গ্রামের লোক Villagers	E	
		অন্যান্য, উল্লেখ করুন Others Specify ________________________________	X	
557	আপনি মোট কত টাকা ধার করেছিলেন/ঋণ নিয়েছিলেন?<br>How much money did you borrow?	☐☐☐☐☐☐ টাকা		
558	এই ঋণ এ সুদ এরহার কত ধরা হয়েছিল?			

	What was the rate of interest?<br>(কোন সুদ না থাকলে 00 এবং জানিনা হলে পুণরায় জিজ্ঞাসা করুন যে পরিবারের কেউ জানে কিনা, কেউ জানলে তার কাছ থেকে শুনে লিখুন, না হলে 99 লিখুন)<br>(put 00 if no interest, if answer is "don't know" ask whether anyone else in the family would know, write down the amount if anyone else knows, otherwise put 99)	১০০ টাকায় ☐☐ টাকা মাসিক/ বাৎসরিক		

Section G: Post natal care প্রসব পরবর্তী সেবা

এখন আমি আপনাকে (নাম) এর জন্মের পর আপনার এবং আপনার নবজাতকের স্বাস্থ্য পরিচর্যা ও সেবার উপর কিছু প্রশ্ন করতে চাই।

Now I will ask few questions on postnatal care of mother and newborn

No.	Questions and filters	Responses	Code	Skip
600	নাম _______এর জন্মের পর পর অতিরিক্ত রক্তক্ষরণ বন্ধ করার জন্য আপনাকে ২/৩ টি ট্যাবলেট দেওয়া হয়েছিল কি?<br>To prevent excessive bleeding after delivery of (NAME), were you given 2/3 tablets?	হ্যাঁ Yes	1	
		না No	2	
601	ডেলিভারির সাথে সাথেই আপনি সেই ট্যাবলেটগুলো খেয়েছিলেন কি?<br>Did you take those tablets just after delivery?	হ্যাঁ Yes	1	
		না No	2	
602	প্রশ্ন 401 দেখুন, ডেলিভারি কি বাড়িতে বা স্বাস্থ্য কেন্দ্র ছাড়া অন্য কোথাও হয়েছিল?<br>CHECK Q-401, was the delivery taken place at home or any place other than health centre?	হ্যাঁ Yes (কোড 01 অথবা 96 বৃত্তায়িত)	1	→606
		না No (কোড 02-10 বৃত্তায়িত)	2	
604	এখন আমি প্রসবের পর আপনার স্বাস্থ্যসেবা সম্পর্কে কথা বলতে চাই। যেমনঃ কেউ আপনার স্বাস্থ্য সম্পর্কে প্রশ্ন করেছিল বা আপনাকে পরীড়্গা করেছিলো।<br>আপনি স্বাস্থ্য কেন্দ্রে থাকা অবস্থায় কেউ কি আপনার স্বাস্থ্য পরীড়্গা করেছিলো?<br>I would like to talk to you about checks on your health after delivery, for example, someone asking you questions about your health or examining you after delivery.<br>Did anyone check on your health while you were still in the facility?	হ্যাঁ Yes	1	
		না No	2	→607
605	আপনি স্বাস্থ্য কেন্দ্র ছেড়ে আসার পর কেউ কি আপনার স্বাস্থ্য পরীড়্গা করেছিলো?<br>Did anyone check on your health after you left the facility?	হ্যাঁ Yes	1	→607
		না No	2	
606	এখন আমি প্রসবের পর আপনার স্বাস্থ্যসেবা সম্পর্কে কথা বলতে চাই। যেমনঃ কেউ আপনার স্বাস্থ্য সম্পর্কে প্রশ্ন করেছিল বা আপনাকে পরীড়্গা করেছিলো।<br>নাম _______ এর জন্মের পর কেউ কি আপনার স্বাস্থ্য পরীড়্গা করেছিলো?<br>I would like to talk to you about checks on your health after delivery, for example, someone asking you questions about your health or examining you after delivery.<br>Did anyone check on your health after you gave birth (NAME)?	হ্যাঁ Yes	1	
		না No	2	→611
607	সে সময় কে আপনার স্বাস্থ্য পরীড়্গা করেছিলো?<br>Who checked on your health at that time?<br>সবচেয়ে যোগ্য ব্যক্তি সর্ম্পকে যাচাই করুন।	স্বাস্থ্য পেশাজীবিঃ Health personnel:		
		পাশ করা ডাক্তার MBBS doctor (Qualified)	01	
		নার্স/ ধাত্রী /প্যারামেডিক<br>Nurse/Midwife/Paramedic	02	

	Probe for most qualified person.<br><br>উত্তর '04' হলে, কমিউনিটি ভিত্তিক দক্ষ ধাত্রী বা CSBA-এর নাম লিখুনঃ<br>If '04' mentioned, write the name of the CSBA.<br><br>______________________________________<br>নাম (Name)	পরিবার কল্যাণ পরিদর্শিকা Family Welfare Visitor (FWV)	03	
		কমিউনিটি ভিত্তিক দক্ষ ধাত্রী (CSBA)	04	
		কমিউনিটি ক্লিনিক ভিত্তিক স্বাস্থ্য সেবা প্রদানকারী (CHCP)	05	
		চিকিৎসা সহকারী / উপসহকারী কমিউনিটি চিকিৎসা কর্মকর্তা Medical Assistant / SACMO	06	
		স্বাস্থ্য সহকারী Health Assistant (HA)	07	
		পরিবার কল্যাণ সহকারী Family Welfare Assistant (FWA)	08	
		অন্যান্য ব্যক্তিঃ Other person:		
		প্রশিক্ষণপ্রাপ্ত টিবিএ Trained TBA	09	
		অপ্রশিক্ষিত টিবিএ Untrained TBA	10	
		অপ্রশিক্ষিত ডাক্তার (গ্রাম ডাক্তার/ পলস্নী চিকিৎসক/হোমিওপ্যাথ/ কবিরাজ) Unqualified doctor	11	
		এন জি ও স্বাস্থ্যকর্মী NGO worker	12	
		অন্যান্য (নির্দিষ্ট করুন) Other:	96	
		জানি না/মনে নেই Don't know/can't remember	97	
608	প্রথম স্বাস্থ্য পরীক্ষাটি কোথায় হয়েছিলো?<br>Where did this first check take place?<br><br>স্থান সর্ম্পকে নিশ্চিত হোন এবং সঠিক উত্তরের কোড বৃত্তায়িত করুন।<br>Probe to identify the type of source and circle the appropriate code.	বাড়ি Home:		
		বাড়িতে Home	01	
		সরকারী সেকটর Public sector:		
		বিশেষায়িত হাসপাতাল / মেডিকেল কলেজ হাসপাতাল Specialized hospital/Medical College	02	
		জেলা হাসপাতাল District hospital	03	
		মাতৃমঙ্গল কেন্দ্র Maternal & Child Welfare Centre (MCWC)	04	
		উপজেলা স্বাস্থ্য কমপ্লেক্স Upazila Health Complex	05	
		পরিবার কল্যাণ কেন্দ্র Family Welfare Centre (FWC)	06	
		স্যাটেলাইট ক্লিনিক/ইপিআই কেন্দ্র Satellite clinic/EPI centre	07	
		কমিউনিটি ক্লিনিক Community clinic	08	
		অন্যান্য (নির্দিষ্ট করুন) Other:	09	
		এন জি ও সেকটর NGO sector:		
		এন জি ও ক্লিনিক NGO static clinic	10	
		এন জি ও স্যাটেলাইট ক্লিনিক NGO satellite clinic	11	
		অন্যান্য (নির্দিষ্ট করুন) Other:	12	
		প্রাইভেট সেকটর Private medical sector:		
		প্রাইভেট হাসপাতাল / ক্লিনিক Private hospital/Clinic	13	
		পাশ করা ডাক্তার MBBS doctor (Qualified)	14	
		অপ্রশিক্ষিত ডাক্তার Traditional doctor	15	
		ফার্মেসী Pharmacy	16	
		অন্যান্য (নির্দিষ্ট করুন) Other:	96	
609	ডেলিভারির কত সময় পর প্রথম স্বাস্থ্য পরীক্ষাটি করা হয়েছিলো?	ঘন্টা Hours [ ][ ]	1	

<table>
<tr><td rowspan="3"></td><td rowspan="3">How long after delivery did the first check take place?
একসপ্তাহের কম হলে, দিনে লিখুন, একদিনের কম হলে, ঘন্টায় লিখুন, এবং এক ঘন্টার কম হলে, ঘন্টার ঘরে '00' লিখুন ।
If less than one week, record in days; If less than one day, record in hours and if less than one hour record '00' in the 'Hour' box.</td><td colspan="2">দিন Days [ ][ ]</td><td>2</td><td></td></tr>
<tr><td colspan="2">সপ্তাহ Weeks [ ][ ]</td><td>3</td><td></td></tr>
<tr><td colspan="2">জানি না Don't know</td><td>97</td><td></td></tr>
<tr><td rowspan="5">610</td><td rowspan="5">প্রসবের পর প্রথম দুই দিনে কোন স্বাস্থ্যকর্মী কি বাসায় অথবা স্বাস্থ্যকেন্দ্রে আপনার নিম্নোক্ত পরীক্ষা গুলি করেছিলঃ
▪ স্তন পরীক্ষা?
▪ স্রাব পরীক্ষা?
▪ তাপমাত্রা মেপেছিল?
▪ প্রসব-পরবর্তী জটিলতা সম্পর্কে ধারনা দিয়েছিল?
During the first two days after delivery, did any health care provider either do the following for you at home or at a health facility:
• Breast examination?
• Check vaginal discharge
• Check temperature?
• Counsel on danger signs?</td><td></td><td>Yes</td><td>No</td><td></td></tr>
<tr><td>স্তন পরীক্ষা Breast examination</td><td>1</td><td>2</td><td></td></tr>
<tr><td>স্রাব পরীক্ষা Check vaginal discharge</td><td>1</td><td>2</td><td></td></tr>
<tr><td>তাপমাত্রা temperature</td><td>1</td><td>2</td><td></td></tr>
<tr><td>প্রসব-পরবর্তী জটিলতা সম্পর্কে বলা Counsel on danger signs</td><td>1</td><td>2</td><td></td></tr>
<tr><td rowspan="3">611</td><td rowspan="3">সাক্ষাতকারগ্রহনকারীঃ প্রশ্ন-205 দেখুন এবং উত্তরদাতার এই গর্ভের ফলাফল সম্পর্কে সঠিক কোড বৃত্তায়িত করুন।
Check question 205 (What was the outcome of this pregnancy?)</td><td colspan="2">জীবিত জন্ম Live birth</td><td>1</td><td></td></tr>
<tr><td colspan="2">মৃত জন্ম Still birth</td><td>2</td><td>→618</td></tr>
<tr><td colspan="2">৭ মাসের আগে নষ্ট Lost before 7 months</td><td>3</td><td>→618</td></tr>
<tr><td rowspan="3">612</td><td rowspan="3">(নাম) _____এর জন্মের ২ (দুই) মাসের মধ্যে কোনো স্বাস্থ্যকর্মী অথবা কোনো অদক্ষ প্রসব সহায়তাকারী কি আপনার বাচ্চার স্বাস্থ্য পরীক্ষা করেছিলেন?
In the two months after (NAME) was born, did any health care provider or a traditional birth attendant check on his/her health?</td><td colspan="2">হ্যাঁ Yes</td><td>1</td><td></td></tr>
<tr><td colspan="2">না No</td><td>2</td><td>→618</td></tr>
<tr><td colspan="2">জানিনা/মনে নাই Don't know/Can't remember</td><td>97</td><td>→618</td></tr>
<tr><td rowspan="4">613</td><td rowspan="4">(নাম) _____ এর জন্মের কত সময় পর প্রথম তার স্বাস্থ্য পরীক্ষা করা হয়েছিলো?
How long after delivery did the first check up of NAME take place?
একসপ্তাহের কম হলে, দিনে লিখুন, একদিনের কম হলে, ঘন্টায় লিখুন, এবং এক ঘন্টার কম হলে, ঘন্টার ঘরে '00' লিখুন ।
If less than one week, record in days; If less than one day, record in hours and if less than one hour record '00' in the 'Hour' box.</td><td colspan="2">জন্মের [ ][ ] ঘন্টা পর Hours after birth</td><td>1</td><td></td></tr>
<tr><td colspan="2">জন্মের [ ][ ] দিন পর Days after birth</td><td>2</td><td></td></tr>
<tr><td colspan="2">জন্মের [ ][ ] সপ্তাহ পর Weeks after birth</td><td>3</td><td></td></tr>
<tr><td colspan="2">জানিনা/মনে নাই Don't know/Can't remember</td><td>97</td><td></td></tr>
<tr><td rowspan="6">614</td><td rowspan="6">কে তখন (নাম) _____এর স্বাস্থ্য পরীক্ষা করেছিলেন?
Who checked on (NAME)'s health at that time?
সবচেয়ে যোগ্য ব্যক্তি সম্পর্কে যাচাই করুন।
Probe for most qualified person.
উত্তর '04' হলে, কমিউনিটি ভিত্তিক দক্ষ ধাত্রী বা CSBA-এর নাম লিখুনঃ
If '04' mentioned, write the name of the CSBA.
____
নাম (Name)</td><td colspan="2">পাশ করা ডাক্তার MBBS doctor (Qualified)</td><td>01</td><td></td></tr>
<tr><td colspan="2">নার্স/ ধাত্রী /প্যারামেডিক Nurse/Midwife/Paramedic</td><td>02</td><td></td></tr>
<tr><td colspan="2">পরিবার কল্যাণ পরিদর্শিকা Family Welfare Visitor (FWV)</td><td>03</td><td></td></tr>
<tr><td colspan="2">কমিউনিটি ভিত্তিক দক্ষ ধাত্রী (CSBA)</td><td>04</td><td></td></tr>
<tr><td colspan="2">কমিউনিটি ক্লিনিক ভিত্তিক স্বাস্থ্য সেবা প্রদানকারী (CHCP)</td><td>05</td><td></td></tr>
<tr><td colspan="2">চিকিৎসা সহকারী / উপসহকারী কমিউনিটি চিকিৎসা কর্মকর্তা Medical Assistant / SACMO</td><td>06</td><td></td></tr>
</table>

		স্বাস্থ্য সহকারী Health Assistant (HA)	07	
		পরিবার কল্যাণ সহকারী Family Welfare Assistant (FWA)	08	
		প্রশিক্ষণপ্রাপ্ত টিবিএ Trained TBA	09	
		অপ্রশিক্ষিত টিবিএ Untrained TBA	10	
		অপ্রশিক্ষিত ডাক্তার (গ্রাম ডাক্তার/ পলস্নী চিকিৎসক/হোমিওপ্যাথ/ কবিরাজ) Unqualified doctor	11	
		এন জি ও স্বাস্থ্যকর্মী NGO worker	12	
		অন্যান্য (নির্দিষ্ট করুন) Other:	96	
		জানিনা/মনে নাই Don't know/Can't remember	97	
615	(নাম) _____এর প্রথম স্বাস্থ্য পরীড়্গাটি কোথায় হয়েছিলো?<br>Where did this first check of (NAME) take place?<br>স্থান সর্ম্পকে নিশ্চিত হোন এবং সঠিক উত্তরের কোড বৃত্তায়িত করুন।<br>Probe to identify the type of source and circle the appropriate code.<br>সরকারি না প্রাইভেট সেকটর নিশ্চিত হতে না পারলে, নীচে স্থানটির নাম লিখুনঃ<br>If unable to determine if public or private sector, write the name of the place:<br>____<br>স্থানের নাম (Name of place)	বাড়ি Home:		
		বাড়িতে Home	01	
		সরকারী সেকটর Public sector:		
		বিশেষায়িত হাসপাতাল / মেডিকেল কলেজ হাসপাতাল Specialized hospital/Medical College	02	
		জেলা হাসপাতাল District hospital	03	
		মাতৃমঙ্গল কেন্দ্র Maternal & Child Welfare Centre (MCWC)	04	
		উপজেলা স্বাস্থ্য কমপ্লেক্স Upazila Health Complex	05	
		পরিবার কল্যাণ কেন্দ্র Family Welfare Centre (FWC)	06	
		স্যাটেলাইট ক্লিনিক/ইপিআই কেন্দ্র Satellite clinic/EPI centre	07	
		কমিউনিটি ক্লিনিক Community clinic	08	
		অন্যান্য (নির্দিষ্ট করুন) Other:	09	
		এন জি ও সেকটর NGO sector:		
		এন জি ও ক্লিনিক NGO static clinic	10	
		এন জি ও স্যাটেলাইট ক্লিনিক NGO satellite clinic	11	
		অন্যান্য (নির্দিষ্ট করুন) Other:	12	
		প্রাইভেট সেকটর Private medical sector:		
		প্রাইভেট হাসপাতাল / ক্লিনিক Private hospital/Clinic	13	
		পাশ করা ডাক্তার MBBS doctor (Qualified)	14	
		অপ্রশিক্ষিত ডাক্তার Traditional doctor	15	
		ফার্মেসী Pharmacy	16	
		অন্যান্য (নির্দিষ্ট করুন) Other:	96	

616	প্রসবের পর প্রথম দুই দিনে কোন স্বাস্থ্যকর্মী কি বাসায় অথবা স্বাস্থ্যকেন্দ্রে নাম ______ এর জন্য নিম্নোক্ত পরীক্ষা গুলি করেছিলঃ<br>▪ নাড়ী পরীক্ষা করেছিল?<br>▪ নবজাতকের স্বাস্থ্য জটিলতা সম্পর্কে পরামর্শ দিয়েছিল?<br>▪ তাপমাত্রা মেপেছিল?<br>▪ আপনাকে বুকের দুধ খাওয়ানোর উপর পরামর্শ দিয়েছিল?<br>▪ বুকের দুধ খাওয়ানো পর্যবেক্ষন করেছিল?<br>▪ শিশুর ওজন নিয়েছিল?<br>During the first two days after delivery, did any health care provider do the following for (NAME) either at		Yes	No	
		নাড়ী পরীক্ষা Examine cord	1	2	
		নবজাতকের স্বাস্থ্য জটিলতা সম্পর্কে পরামর্শ Counsel on danger sign	1	2	
		তাপমাত্রা Temperature	1	2	
		আপনাকে বুকের দুধ খাওয়ানোর উপর পরামর্শ Counsel breast	1	2	

	home or at a facility:<br>• Examine the cord?<br>• Counsel on danger sign<br>• Assess temperature?<br>• Counsel you on breast feeding?<br>• Observe breast feeding?<br>• Assess weight?	feeding			
		বুকের দুধ খাওয়ানো পর্যবেক্ষন Observe breast feeding	1	2	
		শিশুর ওজন Weight	1	2	
617	ডেলিভারির পর প্রথম ২ মাসের মধ্যে আপনি কি এ ধরনের কোনো ভিটামিন ”এ” ক্যাপসুল পেয়েছিলেন? In the first two months after delivery, did you receive a vitamin A capsule like (this/any of these)?<br>সাধারণতঃ যে ধরনের ভিটামিন ”এ” ক্যাপসুল পাওয়া যায়, সেগুলো মা'কে দেখান।<br>Show common types of vitamin A capsules.	হ্যাঁ Yes		1	
		না No		2	
		জানিনা/মনে নাই Don't know/Can't remember		97	
618	এই প্রসবের পর আপনার কোন সমস্যা বা জটিলতা হয়েছিল কি যার জন্য ডাক্তারী চিকিৎসার প্রয়োজন ছিল?<br>After delivery of (NAME) did you develop any problem/complication that required medical treatment?	হ্যাঁ Yes		1	
		না No		2	→620
		জানিনা/মনে নাই Don't know/Can't remember		97	→620
619	আপনার কি ধরণের সমস্যা বা জটিলতা হয়েছিল?<br>Please tell me what was that problem/ complication?<br>উত্তর পড়ে শোনাবেন না।<br>Do not read out the answers<br>জিজ্ঞেস করুনঃ আরও কিছু?<br>ASK: Anything else?<br>সব উত্তরের কোড বৃত্তায়িত করুন।<br>Circle code of all the answers	জ্বর Fever		A	
		যোনীপথে অতিরিক্ত রক্তস্রাব, বড় বড় রক্তের চাকা যাওয়া (রক্তস্রাব কমার চেয়ে বাড়া) Excessive vaginal bleeding, large clots or tissue assed		B	
		যোনীপথে দুর্গন্ধযুক্ত স্রাব Foul smelling vaginal discharge		C	
		তলপেটে তীব্র ব্যাথা Severe lower abdominal pain		D	
		শ্বাস নিতে কষ্ট, ক্লান্তি/অবসন্নতা, বুক ধরফরানি এবং দুর্বলতা Breathlessness, tiredness, palpitation and weakness		E	
		নিস্তেজ/অজ্ঞানভাব Fainting		F	
		ফিট/খিঁচুনী Fits and convulsion		G	
		অন্যান্য (নির্দিষ্ট করুন)<br>Other:______________________________		X	
620	এই সমস্যা/অসুবিধা/জটিলতার জন্য আপনি কোন চিকিৎসা করিয়েছেন কি?<br>Did you seek any sort of treatment for this problem/complication?	হ্যাঁ Yes		1	
		না No		2	→
		জানিনা/মনে নাই Don't know/Can't remember		97	→
621	আপনি কার কাছে চিকিৎসা করিয়েছেন?<br>From whom you received treatment for this problem/complication?<br>জিজ্ঞেস করুনঃ আর কেউ?<br>Anyone else?<br>ব্যক্তি সর্ম্পকে নিশ্চিত হোন এবং সঠিক উত্তরের কোড বৃত্তায়িত করুন।<br>Probe to identify each type of person and check all	পাশ করা ডাক্তার MBBS doctor (Qualified)		A	
		নার্স/ ধাত্রী /প্যারামেডিক Nurse/Midwife/Paramedic		B	
		পরিবার কল্যাণ পরিদর্শিকা Family Welfare Visitor (FWV)		C	
		কমিউনিটি ভিত্তিক দক্ষ ধাত্রী (CSBA)		D	

	mentioned.<br>পরামর্শদাতার নাম লিখুনঃ<br>write the name of the person who advised you<br>____<br>নাম (Name)	কমিউনিটি ক্লিনিক ভিত্তিক স্বাস্থ্য সেবা প্রদানকারী (CHCP)	E	
		চিকিৎসা সহকারী / উপসহকারী কমিউনিটি চিকিৎসা কর্মকর্তা Medical Assistant / SACMO	F	
		স্বাস্থ্য সহকারী Health Assistant (HA)	G	
		পরিবার কল্যাণ সহকারী Family Welfare Assistant (FWA)	H	
		প্রশিক্ষণপ্রাপ্ত টিবিএ Trained TBA	I	
		অপ্রশিক্ষিত টিবিএ Untrained TBA	J	
		অপ্রশিক্ষিত ডাক্তার (গ্রাম ডাক্তার/ পলস্নী চিকিৎসক/ কবিরাজ) Unqualified doctor	K	
		এন জি ও স্বাস্থ্যকর্মী NGO worker	L	
		পরিবারের সদস্য/আত্মীয়/প্রতিবেশী Family member / Relative / neighbour	M	
		ফার্মেসি Pharmacy	N	
		অন্যান্য (নির্দিষ্ট করম্নন) Other:	X	
		জানিনা/মনে নাই Don't know	Y	
622	এই চিকিৎসা আপনি কোথায় করিয়েছেন?<br>From where did you receive care for this problem/complication?<br>স্থান সর্ম্পকে নিশ্চিত হোন এবং সঠিক উত্তরের কোড বৃত্তায়িত করম্নন।<br>Probe to identify the type of source and circle the appropriate code.<br>সরকারি না প্রাইভেট সেকটর নিশ্চিত হতে না পারলে, নীচে স্থানটির নাম লিখুনঃ<br>If unable to determine if public or private sector, write the name of the place:<br>____<br>স্থানের নাম (Name of place)	<u>বাড়ি Home:</u>		
		বাড়িতে Home	A	→623
		<u>সরকারী সেকটর Public sector:</u>		
		বিশেষায়িত হাসপাতাল / মেডিকেল কলেজ হাসপাতাল Specialized hospital/Medical College	B	
		জেলা হাসপাতাল District hospital	C	
		মাতৃমঙ্গল কেন্দ্র Maternal & Child Welfare Centre (MCWC)	D	
		উপজেলা স্বাস্থ্য কমপ্লেক্স Upazila Health Complex	E	
		পরিবার কল্যাণ কেন্দ্র Family Welfare Centre (FWC)	F	
		স্যাটেলাইট ক্লিনিক/ইপিআই কেন্দ্র Satellite clinic/EPI centre	G	
		কমিউনিটি ক্লিনিক Community clinic	H	
		অন্যান্য (নির্দিষ্ট করম্নন) Other:	I	
		<u>এন জি ও সেকটর NGO sector:</u>		
		এন জি ও ক্লিনিক NGO static clinic	J	
		এন জি ও স্যাটেলাইট ক্লিনিক NGO satellite clinic	K	
		অন্যান্য (নির্দিষ্ট করম্নন) Other:	L	
		<u>প্রাইভেট সেকটর Private medical sector:</u>		
		প্রাইভেট হাসপাতাল / ক্লিনিক Private hospital/Clinic	M	

		পাশ করা ডাক্তার MBBS doctor (Qualified)	N	
		অপ্রশিক্ষিত ডাক্তার Unqualified doctor	O	
		ফার্মেসী Pharmacy	P	
		অন্যান্য (নির্দিষ্ট করুন) Other:________________	X	

প্রসব পরবর্তী জটিলতায় চিকিৎসা বাবদ খরচ সমূহ **Treatment cost for postnatal complication**

No.	Questions and filters	Responses				Code	Skip
623	আপনি মোট কতবার প্রসব পরবর্তী জটিলতার জন্য চিকিৎসা গ্রহন করেছেন? How many times you have received treatment for postnatal complication?	বার times				☐☐	
		জানিনা/মনে নাই Don't know				97	
624	আপনার প্রসব পরবর্তী সময়ে চিকিৎসার জন্য কি কোন খরচ হয়েছিল? Did you incur any cost for the treatment during postnatal period?	হ্যাঁ Yes				1	
		না No				2	→end
এখন আমি আপনার কাছে সেবা গ্রহন বাবদ খরচ সমূহ সম্পর্কে বিস্তারিত জানতে চাইব। (ভিজিট অনুযায়ী উল্লেখ করুন)<br>I will now ask you details about the costs that you had incurred for care seeking (Please mention by visit).							
625	ডাক্তার বা সেবাপ্রদানকারীর ফি বাবদ আপনার কোন খরচ হয়েছিল কি? হ্যাঁ হলে, ডাক্তার বা সেবাপ্রদানকারীর ফি বাবদ মোট কত টাকা খরচ হয়েছিল?<br>Did you incur any cost for consulting a Doctor or a Service provider? How much did you pay to the doctor or service provider as fees?	১ম ভিজিট<br>1st visit	২য় ভিজিট<br>2nd visit	৩য় ভিজিট<br>3rd visit	৪র্থ ভিজিট<br>4th visit		
		হ্যাঁ Yes .... 1<br>না No .... 2<br>Tk.__________	হ্যাঁ Yes..........1<br>না No...........2<br>Tk.__________	হ্যাঁ Yes .... 1<br>না No .... 2<br>Tk.__________	হ্যাঁ Yes..........1<br>না No ....2<br>Tk.__________		
626	আপনাকে কি হাসপাতালে ভর্তি হতে হয়েছিল? আপনাকে কতদিন হাসপাতালে থাকতে হয়েছিল? বেড/কেবিন ভাড়া বাবদ কত খরচ হয়েছিল?<br>Were you admitted at the hospital? How long did you have to stay at the hospital? How much Did you pay for the bed/cabin?	১ম ভিজিট<br>1st visit	২য় ভিজিট<br>2nd visit	৩য় ভিজিট<br>3rd visit	৪র্থ ভিজিট<br>4th visit		
		হ্যাঁ Yes .... 1<br>না No .... 2<br>হ্যাঁ হলে, হাসপাতালে কতদিন ছিলেন?<br>Days of hospital stay |___|___| Tk.__________	হ্যাঁ Yes..........1<br>না No...........2<br>হ্যাঁ হলে, হাসপাতালে কতদিন ছিলেন?<br>Days of hospital stay |___|___| Tk.__________	হ্যাঁ Yes .... 1<br>না No .... 2<br>হ্যাঁ হলে, হাসপাতালে কতদিন ছিলেন?<br>Days of hospital stay |___|___| Tk.__________	হ্যাঁ Yes..........1<br>না No ....2<br>হ্যাঁ হলে, হাসপাতালে কতদিন ছিলেন?<br>Days of hospital stay |___|___| Tk.__________		
627	আপনার কি কোন অপারেশন হয়েছিল? অপারেশন বাবদ কত খরচ হয়েছিল?<br>Did you undergo any surgery? How much did you have to pay for operation?	১ম ভিজিট<br>1st visit	২য় ভিজিট<br>2nd visit	৩য় ভিজিট<br>3rd visit	৪র্থ ভিজিট<br>4th visit		
		হ্যাঁ Yes .... 1<br>না No .... 2<br>হ্যাঁ হলে, কত খরচ হয়েছিল?<br>Tk.__________	হ্যাঁ Yes..........1<br>না No...........2<br>হ্যাঁ হলে, কত খরচ হয়েছিল?<br>Tk.__________	হ্যাঁ Yes .... 1<br>না No .... 2<br>হ্যাঁ হলে, কত খরচ হয়েছিল?<br>Tk.__________	হ্যাঁ Yes..........1<br>না No ....2<br>হ্যাঁ হলে, কত খরচ হয়েছিল?<br>Tk.__________		
628	আপনাকে কি কোন ঔষধ /ইঞ্জেকশান/ স্যালাইন খেতে হয়েছিল?<br>এসব ক্রয় বাবদ কত খরচ হয়েছিল?<br>Did you take any drugs/ medicine/	১ম ভিজিট<br>1st visit	২য় ভিজিট<br>2nd visit	৩য় ভিজিট<br>3rd visit	৪র্থ ভিজিট<br>4th visit		
		হ্যাঁ Yes .... 1<br>না No 2	হ্যাঁ Yes..........1<br>না No...........2	হ্যাঁ Yes .... 1<br>না No 2	হ্যাঁ Yes..........1<br>না No 2		

	injection/ saline? How much did you pay for buying them?	হ্যাঁ হলে, কত খরচ হয়েছিল?<br>Tk._________	হ্যাঁ হলে, কত খরচ হয়েছিল?<br>Tk._________	হ্যাঁ হলে, কত খরচ হয়েছিল?<br>Tk._________	হ্যাঁ হলে, কত খরচ হয়েছিল?<br>Tk._________	
629	হাসপাতালে যাওয়ার অথবা ফিরে আসার পথে যাতায়াত বাবদ কোন খরচ হয়েছিল কি? যাতায়াত বাবদ মোট কত খরচ হয়েছিল?<br>Did you incur any transport cost while travelling to and returning from the provider? How much did you pay for transport cost in total?	১ম ভিজিট<br>1st visit<br>হ্যাঁ Yes .... 1<br>না No .... 2<br>হ্যাঁ হলে, কত খরচ হয়েছিল?<br>Tk._________	২য় ভিজিট<br>2nd visit<br>হ্যাঁ Yes..........1<br>না No...........2<br>হ্যাঁ হলে, কত খরচ হয়েছিল?<br>Tk._________	৩য় ভিজিট<br>3rd visit<br>হ্যাঁ Yes .... 1<br>না No .... 2<br>হ্যাঁ হলে, কত খরচ হয়েছিল?<br>Tk._________	৪র্থ ভিজিট<br>4th visit<br>হ্যাঁ Yes..........1<br>না No ....2<br>হ্যাঁ হলে, কত খরচ হয়েছিল?<br>Tk._________	
630	এই সেবাগ্রহণ করার সময়ে খাবার অথবা পানীয় কিনে খেতে কোন খরচ হয়েছিল কি? খাবার অথবা পানীয় কিনে খেতে কত খরচ হয়েছিল?<br>Did you incur any cost on food or beverages? How much did you pay for food or beverages?	১ম ভিজিট<br>1st visit<br>হ্যাঁ Yes .... 1<br>না No .... 2<br>হ্যাঁ হলে, কত খরচ হয়েছিল?<br>Tk._________	২য় ভিজিট<br>2nd visit<br>হ্যাঁ Yes..........1<br>না No...........2<br>হ্যাঁ হলে, কত খরচ হয়েছিল?<br>Tk._________	৩য় ভিজিট<br>3rd visit<br>হ্যাঁ Yes .... 1<br>না No .... 2<br>হ্যাঁ হলে, কত খরচ হয়েছিল?<br>Tk._________	৪র্থ ভিজিট<br>4th visit<br>হ্যাঁ Yes..........1<br>না No ....2<br>হ্যাঁ হলে, কত খরচ হয়েছিল?<br>Tk._________	
631	চিকিৎসা সেবা নিতে যাওয়ার পথে অথবা সেখান থেকে ফিরে আসার পথে আপনাকে হোটেল বা অন্যকোথাও রাত কাটাতে হয়েছিল কি? হোটেল বা অন্যকোথাও থাকা বাবদ কত খরচ হয়েছিল?<br>Did you spend night away from home either while going there or while coming back? Did you incur any cost for staying over? How much did you pay?	১ম ভিজিট<br>1st visit<br>হ্যাঁ Yes .... 1<br>না No .... 2<br>হ্যাঁ হলে, কত খরচ হয়েছিল?<br>Tk._________	২য় ভিজিট<br>2nd visit<br>হ্যাঁ Yes..........1<br>না No...........2<br>হ্যাঁ হলে, কত খরচ হয়েছিল?<br>Tk._________	৩য় ভিজিট<br>3rd visit<br>হ্যাঁ Yes .... 1<br>না No .... 2<br>হ্যাঁ হলে, কত খরচ হয়েছিল?<br>Tk._________	৪র্থ ভিজিট<br>4th visit<br>হ্যাঁ Yes..........1<br>না No ....2<br>হ্যাঁ হলে, কত খরচ হয়েছিল?<br>Tk._________	
632	আপনাকে কি রক্ত/ মল/ মুত্র/ আলট্রাসনোগ্রাম পরীক্ষা করাতে হয়েছিল কি? এসব পরীক্ষা করাতে কত খরচ হয়েছিল?<br>Did you spend any money for blood/stool/urine test?<br>How much did you spend for each of these tests?<br>যদি উত্তর না জানা থাকে তবে 'DK' লিখুন<br>If not known then write'DK'	১ম ভিজিট<br>1st visit<br>হ্যাঁ Yes .... 1<br>না No .... 2<br>১। রক্ত পরীক্ষা<br>Blood test<br>Tk.__________<br>২। মুত্র পরীক্ষা<br>Urine test<br>Tk.__________<br>৩। মল পরীক্ষা<br>Stool test<br>Tk.__________<br>৪। আলট্রাসনোগ্রাম<br>Ultra sonogram<br>Tk.__________	২য় ভিজিট<br>2nd visit<br>হ্যাঁ Yes..........1<br>না No...........2<br>১। রক্ত পরীক্ষা<br>Blood test<br>Tk.__________<br>২। মুত্র পরীক্ষা<br>Urine test<br>Tk.__________<br>৩। মল পরীক্ষা<br>Stool test<br>Tk.__________<br>৪। আলট্রাসনোগ্রাম<br>Ultra sonogram<br>Tk.__________	৩য় ভিজিট<br>3rd visit<br>হ্যাঁ Yes .... 1<br>না No .... 2<br>১। রক্ত পরীক্ষা<br>Blood test<br>Tk.__________<br>২। মুত্র পরীক্ষা<br>Urine test<br>Tk.__________<br>৩। মল পরীক্ষা<br>Stool test<br>Tk.__________<br>৪। আলট্রাসনোগ্রাম<br>Ultra sonogram<br>Tk.__________	৪র্থ ভিজিট<br>4th visit<br>হ্যাঁ Yes..........1<br>না No ....2<br>১। রক্ত পরীক্ষা<br>Blood test<br>Tk.__________<br>২। মুত্র পরীক্ষা<br>Urine test<br>Tk.__________<br>৩। মল পরীক্ষা<br>Stool test<br>Tk.__________<br>৪। আলট্রাসনোগ্রাম<br>Ultra sonogram<br>Tk.__________	
633	আপনাকে কি স্যালাইন/রক্ত/অক্সিজেন দিতে হয়েছিল? এগুলো বাবদ কত খরচ হয়েছিল?<br>Did you spend any money for saline	১ম ভিজিট<br>1st visit<br>হ্যাঁ Yes .... 1<br>না No 2	২য় ভিজিট<br>2nd visit<br>হ্যাঁ Yes..........1<br>না No...........2	৩য় ভিজিট<br>3rd visit<br>হ্যাঁ Yes .... 1<br>না No 2	৪র্থ ভিজিট<br>4th visit<br>হ্যাঁ Yes..........1<br>না No 2	

	infusion or blood transfusion or oxygen inhalation during your stay at health facility? How much did you sopned for each of these?<br><br>যদি উত্তর না জানা থাকে তবে 'DK' লিখুন<br>If not known then write'DK'	১। স্যালাইন<br>Saline<br>Tk.__________<br>২। রক্ত<br>Blood transfusion<br>Tk.__________<br>৩। অক্সিজেন<br>Oxygen<br>Tk.__________	১। স্যালাইন<br>Saline<br>Tk.__________<br>২। রক্ত<br>Blood transfusion<br>Tk.__________<br>৩। অক্সিজেন<br>Oxygen<br>Tk.__________	১। স্যালাইন<br>Saline<br>Tk.__________<br>২। রক্ত<br>Blood transfusion<br>Tk.__________<br>৩। অক্সিজেন<br>Oxygen<br>Tk.__________	১। স্যালাইন<br>Saline<br>Tk.__________<br>২। রক্ত<br>Blood transfusion<br>Tk.__________<br>৩। অক্সিজেন<br>Oxygen<br>Tk.__________	
634	ওয়ার্ড বয়, এটেনডেন্ট (সাহায্যকারী)/ দাড়োয়ানদের উপহার/বকশিশ বাবদ কোন খরচ হয়েছিল কি? কত খরচ হয়েছিল?<br>Did you pay for tips to ward boy, attendant, gate keeper? How much cost did you incur?	১ম ভিজিট<br>1st visit<br>হ্যাঁ Yes .... 1<br>না No .... 2<br>হ্যাঁ হলে, কত খরচ হয়েছিল?<br>Tk.__________	২য় ভিজিট<br>2nd visit<br>হ্যাঁ Yes.........1<br>না No...........2<br>হ্যাঁ হলে, কত খরচ হয়েছিল?<br>Tk.__________	৩য় ভিজিট<br>3rd visit<br>হ্যাঁ Yes .... 1<br>না No .... 2<br>হ্যাঁ হলে, কত খরচ হয়েছিল?<br>Tk.__________	৪র্থ ভিজিট<br>4th visit<br>হ্যাঁ Yes.........1<br>না No ....2<br>হ্যাঁ হলে, কত খরচ হয়েছিল?<br>Tk.__________	
635	আপনার কোন সাহায্যকারী ছিল কি?<br>Did you have any attendant?	হ্যাঁ Yes .... 1<br>না No .... 2→	হ্যাঁ Yes.........1<br>না No...........2→	হ্যাঁ Yes .... 1<br>না No .... 2→	হ্যাঁ Yes.........1<br>না No ....2→	If 2→636
636	আপনার এটেনডেন্ট (সাহায্যকারী) আপনাকে সঙ্গ দেওয়ার সময়কালীন কোন অর্থনৈতিক ড়্গতির সম্মুখিন হয়েছিল? (অর্থাৎ সাহায্যকারী কোন কাজ করে এবং আপনার সাথে থাকার কারনে সে পারিশ্রমিক থেকে বঞ্চিত) হয়ে থাকলে তার পরিমাণ?<br>Did your attendant lose any income as a result of time spent with you on seeking care? If yes, how much did he/she lose?	১ম ভিজিট<br>1st visit<br>হ্যাঁ Yes .... 1<br>না No .... 2<br>হ্যাঁ হলে, কত টাকা থেকে বঞ্চিত হয়েছিল?<br>Tk.__________	২য় ভিজিট<br>2nd visit<br>হ্যাঁ Yes.........1<br>না No...........2<br>হ্যাঁ হলে, কত টাকা থেকে বঞ্চিত হয়েছিল?<br>Tk.__________	৩য় ভিজিট<br>3rd visit<br>হ্যাঁ Yes .... 1<br>না No .... 2<br>হ্যাঁ হলে, কত টাকা থেকে বঞ্চিত হয়েছিল?<br>Tk.__________	৪র্থ ভিজিট<br>4th visit<br>হ্যাঁ Yes.........1<br>না No ....2<br>হ্যাঁ হলে, কত টাকা থেকে বঞ্চিত হয়েছিল?<br>Tk.__________	

637	আপনার এই চিকিৎসাকালীন সময়ে যে সকল খরচ হয়েছিল, তা কিভাবে মিটিয়েছিলেন?<br>(একাধিক উত্তর গ্রহণযোগ্য)<br>How did you meet the expenditure that was incurred due to careseeking for your treatment?<br>(multiple answers acceptable)	সুদ সহ ঋণ এর মাধ্যমে/ধার করে Loan with Interest	A	→for any option other than A &B→ end here
		সুদ বিহীন ঋণ এর মাধ্যমে/ধার করে Loan without interest	B	
		সঞ্চয় থেকে বা বাড়ীর অন্যান্য খরচ বাচিয়ে Savings after all household expenses	C	
		সম্পত্তি বিক্রয় করে Selling land and assets	D	
		আত্মীয় / বন্ধু এর কাছ থেকে সাহায্য হিসাবে Help from Relatives/ Friends	E	
		নিয়মিত আয় থেকে Regular Income	F	
		নিজস্ব জিনিস বিক্রি করে Sold Personal Belongings	G	
		গৃহ পালিত পশু বিক্রি করে Sold Livestock.	H	
		গাছ অথবা উৎপাদিত শস্য বিক্রি কওে Sold agricultural product/tree	I	
		স্থায়ী সম্পদ বিক্রি করে Sold permanent assets	J	
		জমি বা সম্পদ জামানত রেখে Mortgage of Assets/land	K	
		অন্যান্য, উল্লেখ করচন Others Specify	X	

		______________________________		
638	আপনি কোথা থেকে ধার করেছিলেন/ঋণ নিয়েছিলেন? Where did you take the loan from? (একাধিক উত্তর গ্রহণযোগ্য) (multiple answers acceptable)	মাইক্রোক্রেডিট সংস্থা (এন জি ও) Microcredit (NGO) ব্যাংক Bank	A	
		গ্রামের মহাজন Village Mohajon	B	
		আত্মীয় Relative	C	
		প্রতিবেশী Neighbour	D	
		গ্রামের লোক Villagers	E	
		অন্যান্য , উল্লেখ করুন Others Specify ______________________________	X	
639	আপনি মোট কত টাকা ধার করেছিলেন/ঋণ নিয়েছিলেন? How much money did you borrow?	☐☐☐☐☐☐ টাকা		
640	এই ঋণ এ সুদ এরহার কত ধরা হয়েছিল? What was the rate of interest? (কোন সুদ না থাকলে 00 এবং জানিনা হলে পুণরায় জিজ্ঞাসা করুন যে পরিবারের কেউ জানে কিনা , কেউ জানলে তার কাছ থেকে শুনে লিখুন , না হলে 99 লিখুন) (put 00 if no interest, if answer is “don’t know” ask whether anyone else in the family would know, write down the amount if anyone else knows, otherwise put 99)	১০০ টাকায় ☐☐ টাকা মাসিক/ বাৎসরিক		

Section H: Social Capital

No.	Questions and filters	Responses	Code	Skip
700	আপনি কি বর্তমানে কোন সমিতি বা দলের সদস্য হিসাবে আছেন? Do you have any membership in any financial or social group?	হ্যাঁ Yes	1	
		না No	2	→706
701	আপনি কতগুলো দলের সদস্য? (আর্থিক , সামাজিক ,ক্লাব ,বিভিন্ন কমিটি) Of how many such groups are you or any one in your household a member?	☐☐		
702	এগুলোর মধ্যে কোনটি আপনার কাছে সবচেয়ে বেশি গুরুত্বপূর্ণ? Which one is the most important to your household?	______________________________ [দলের নাম/Name of group]		
703	দলের সকল সদস্য কি একই ধর্ম , লিঙ্গ এবং সমান শিক্ষগত যোগ্যতা সম্পন্ন? Are most of them of the same religion, gender & same level of education?	হ্যাঁ Yes	1	
		না No	2	
704	দলের সবার পেশা কি একই ধরনের? Do members mostly have the same occupation?	হ্যাঁ Yes	1	
		না No	2	
705	আপনাদের এই দলটি কি পাশের এলাকার/গ্রামের অন্যান্য দলের সাথেও যোগাযোগ রাখে? Does this group work with or interact with groups outside the village/neighborhood?	না No	1	
		হ্যাঁ , মাঝেমধ্যে Yes, occasionally	2	
		হ্যাঁ , প্রায়ই Yes, frequently	3	
706	বর্তমানে আপনার এমন কয়জন বন্ধু আছে যাদের কাছে আপনি সব ধরনের কথা শেয়ার করতে পারেন বা সাহায্য চাইতে			

	পারেন? About how many close friends do you have these days? These are people you feel at ease with, can talk to about private matters, or call on for help.			
707	যদি হঠাৎ করে টাকা ঋণ নেওয়ার দরকার (৫-১০ হাজার টাকা) হয় তাহলে তা বন্ধুদের কাছ থেকে তা পাওয়া সম্ভব? If you suddenly needed to borrow a small amount of money are there people beyond your immediate household and close relatives to whom you could turn and who would be willing and able to provide this money?	অবশ্যই Definitely	1	
		পেতে পারি Probably	2	
		ঠিক জানি নাUnsure	3	
		না্ও পেতে পারি Probably not	4	
		পাবো না Definitely not	5	
	**Trust and Solidarity**			
708	আপনার কি মনে হয় আজকাল মানুষকে বিশ্বাস করা যায়? Generally speaking, would you say that most people can be trusted?	হ্যাঁ Yes	1	
		না No	2	
709	গ্রামের অধিকাংশ মানুষ প্রয়োজনে সাহায্য করতে প্রস্তুত? Most people in this village/neighborhood are willing to help if you need it.	অবশ্যই Agree strongly	1	
		করতেও পারে Agree somewhat	2	
		করতেও পারে নাও করতে পারে Neither agree nor disagree	3	
		নাও করতে পারে Disagree somewhat	4	
		করবে না Disagree strongly	5	
710	গ্রামে এমন কিছু মানুষ আছে যাদের থেকে সতর্ক থাকা দরকার, তারা যে কোন সময় ঠকাতে পারে। In this village/neighborhood, one has to be alert or someone is likely to take advantage of you.	অবশ্যই Agree strongly	1	
		থাকা উচিৎ Agree somewhat	2	
		থাকলেও চলে না থাকলেও চলে Neither agree nor disagree	3	
		না থাকলেও চলে Disagree somewhat	4	
		প্রয়োজন নাই Disagree strongly	5	
711	সরকারি কর্মকর্তা-কর্মচারীদের প্রতি আপনার আস্থা/বিশ্বাস কতটুকু? How much do you trust Local government officials?	অনেক বেশি To a very great extent	1	
		বেশি To a great extent	2	
		মোটামুটি Neither great nor small extent	3	
		খুব একটা না To a small extent	4	
		একদমই না To a very small extent	5	
	**Collective Action and Cooperation**			
712	যদি এলাকায় এমন কোন যাতে আপনার সরাসরি কোন লাভ নাই কিন্তু সামগ্রিক উপকার হবে সে ক্ষেত্রে কি আপনি সেই কাজে আপনার সময় বা টাকা খরচ করবেন? If a community project does not directly benefit you but has benefits for many others in the village/neighborhood, would you contribute time or money to the project?	হ্যাঁ Yes	1	
		না No	2	
713	গত ১২ মাসে আপনি বা আপনার পরিবারের কোন সদস্য কি এমন কোন কাজে অংশ নিয়েছেন যা দ্বারা আপনার এলাকার	হ্যাঁ Yes	1	
		না No	2	

	মানুষ উপকৃত হয়েছে?<br>In the past 12 months did you or any one in your household participate in any communal activities, in which people came together to do some work for the benefit of the community?			
714	যদি কোন কারনে আপনার এলাকার স্বাস্থ্য কেন্দ্রটি ক্ষতিগ্রস্থ হয় তাহলে এলাকার মানুষ কি তা মেরামতের উদ্যোগ নেবে?<br>If problem happens to the health facility in this community, how likely is it that people will cooperate to try to solve the problem?	অবশ্যই Agree strongly	1	
		করতেও পারে Agree somewhat	2	
		করতেও পারে নাও করতে পারে Neither agree nor disagree	3	
		নাও করতে পারে Disagree somewhat	4	
		করবে না Disagree strongly	5	
	**Information and Communication**			
715	গত সপ্তাহে আপনি কতগুলো ফোন কল করেছেন বা রিসিভ করেছেন?<br>In the past week, how many times have you made or received a phone call?	☐☐		
716	কাদের সাথে আপনি ফোনে বেশি যোগাযোগ করেন? (একাধিক উত্তর হতে পারে)<br>With whom you communicate more over phone?	পরিবারের সদস্য Family Members	1	
		আত্মীয়-স্বজন Relatives	2	
		বন্ধু-বান্ধব Friends	3	
		সহকর্মী Colleagues/business partners	4	
		অন্যান্য Others________________________	5	
717	স্বাস্থ্যসেবা সম্পর্কে জানার ক্ষেত্রে কোন ০৩টি মাধ্যমকে আপনি বেশি সক্রিয় মনে করেন?<br>What are your three main sources of information about health care services?	টেলিভিশন TV	1	
		রেডিও Radio	2	
		সরকারি স্বাস্থ্যকর্মী GoB Health Workers	3	
		এনজিও কমী NGO workers	4	
		ইন্টারনেট Internet	5	
	**Social Cohesion and Inclusion**			
718	শিক্ষা, অর্থনৈতিক অবস্থা, সামাজিক অবস্থান, ধর্ম, গোষ্ঠী এসবের কারনে আপনার এলাকার মানুষজনে মাধ্যে কি পরিমাণ বিভাজন আছে?<br>What is the level of discrimination among your community due to educational, religious, social status and other issues?	অনেক বেশি To a very great extent	1	
		বেশি To a great extent	2	
		মোটামুটি Neither great nor small extent	3	
		খুব একটা না To a small extent	4	
		একদমই না To a very small extent	5	
719	এই বিভাজন কি কোন ধরনের সমস্যা বা দ্বন্দ্ব সৃষ্টি করে?<br>Do any of these differences cause problems?	হ্যাঁ Yes	1	
		না No	2	
720	কোন ০২টি কারনে সবচেয়ে বেশি সমস্যা হয়?<br>Which two differences most often cause problems?	শিক্ষা Education	1	
		ধর্ম Religion	2	
		জাতি Ethnicity	3	
		সামাজিক অবস্থান Social Status	4	
		গোষ্ঠা Clan	5	
		অন্যান্য Others________________________	6	
721	গত মাসে আপনি বা আপনার পরিবারের কেউ কি কোথাও	হ্যাঁ Yes	1	

	দা্ওয়াত খেতে গিয়েছিলেন বা কাউকে দা্ওয়াত দিয়েছিলেন?<br>In the past month have you got together with people to have food or drinks, either in their home or in a public place?	না No	2	
722	যাদের সাথে দেখা হয়েছিলো তারা কি সাবই একই ধরনের সামাজিক, আর্থিক, শিক্ষা ও ধর্মীয় অবস্থা সম্পন্ন মানুষ ছিলেন?<br>Were all of them from same social, economic, educational and religious background?	হ্যাঁ Yes	1	
		না No	2	
723	আপনার বাড়িতে কি আপনি নিরাপদ বোধ করেন?<br>In general, how safe from crime and violence do you feel when you are alone at home?	হ্যাঁ Yes	1	
		না No	2	
	**Empowerment and Political Action**			
724	আপনি নিজেকে কতটুকু সুখী মনে করেন?<br>In general, how happy do you consider yourself to be?	অনেক বেশি To a very great extent	1	
		বেশি To a great extent	2	
		মোটামুটি Neither great nor small extent	3	
		খুব একটা না To a small extent	4	
		একদমই না To a very small extent	5	
725	আপনি কি মনে করেন আপনার বর্তমান আবস্থা উল্লেখযোগ্যভাবে পরিবর্তন করার সামর্থ্য আপনার আছে?<br>Do you feel that you have the power to make important decisions that change the course of your life?	অনেক বেশি To a very great extent	1	
		বেশি To a great extent	2	
		মোটামুটি Neither great nor small extent	3	
		খুব একটা না To a small extent	4	
		একদমই না To a very small extent	5	
726	গত ১২ মাসে কি আপনারা এলাকার মানুষ স্থানীয় নেতা বা সরকারী কোন অফিসে এমন কোন আবেদন করেছেন যাতে সবার উপকার হতে পারে?<br>In the past 12 months, how often have people in this village/neighborhood got together to jointly petition government officials or political leaders for something benefiting the community?	এবারও না Never	1	
		একবার Once	2	
		কয়েকবার (<৫)A few times (<5)	3	
		অনেকবার (>৫) Many times (>5)	4	
727	সর্বশেষ নির্বাচনে আপনি কি ভোট দিয়েছিলেন?<br>Did you vote on the last local/national election?	হ্যাঁ Yes	1	
		না No	2	

ধন্যবাদ। **Thank you for participation.**

Time of ending of interview: ☐☐:☐☐ **(24 hour)**

Tool 5: KII guideline for UP Chairman

Objective(s) of the interview:

1. Understand his level of involvement into local health system
2. His willingness to improvement of health services
3. Availability of services
4. Status of local social capital
5. Understand the level of awareness about health policies
6. Understand challenges facing to provide adequate & quality service
7. Suggestion to improve services.

Guiding questions

24. In general, how would you describe health facilities of this area?
25. In your opinion, has health services in this area, stayed the same, or declined over the past few years? (How & why)
26. What are major health issues you see in your community? (Common diseases, illness)
27. How do you see your role in relation to health care of this locality?
    - Do you have any experience?
    - What is your influence?
    - Do you feel any responsibility?
    - What are the three biggest health problems in this community?
28. Is there any coordination between the local government and health sectors in this area?
    - If yes, how it works?
    - If no, what are some of the barriers you observed that impacted the coordination?
29. Did you ever participate in local level planning meeting?
    - If yes, what outcomes would you like to see from the planning meeting?
    - If no, did you ever heard about the meeting?
30. Where do people go for health care and what is their experience like?
    - What is quality of care at major sources of care/health facilities?
    - Are services located in convenient locations?
31. Do you think GoB health centres had adequate staff, medicine and other necessary things to perform their required activities?
32. What is your role in the community mobilization and sensitization activities for seeking health care from health facilities?
33. What types of community mobilization and sensitization activities did you observe?
34. Which organizations participate in community mobilization and sensitization activities?
35. What are community advocacy groups in the area like?

- What kinds of activism are there among underserved people? What issues have mobilized people?
- What groups/organizations are involved with underserved?
- Do community/advocacy groups work together? How?

36. How will you describe the relationship among community people? (Helpful to each other or not)
37. What barriers, if any, exist to improving health services in this area?
38. What needs to be done to address these issues?
    - Possible probe: What specific actions, policy or funding priorities would you support because they would contribute to a healthier service center?
39. How would you go about trying to motivate people in your community to more actively support facility based care seeking, delivery?
40. Do you think the environment or the quality of the health facility have anything to do with it?
41. What do you think is the best way to improve health services in health centers?
42. Are you aware of any major new developments taking place in the health system?
43. Is there any policy or guideline of HtR areas healthcare system?
    - If yes, can please tell something about that?
    - If no, do you think there should be a policy? (Why)
    - What should be addressed in that policy or guideline?

THANK YOU for taking the time to participate in this interview.

Interview guideline for interviewing UP Chairman

Objective(s) of the interview:

1. Understand his level of involvement into local health system
2. His willingness to improvement of health services
3. Availability of services
4. Status of local social capital
5. Understand the level of awareness about health policies
6. Understand challenges facing to provide adequate & quality service
7. Suggestion to improve services.

Guiding questions

১. আপনার এলাকার স্বাস্থ্য সেবার এবং স্বাস্থ্য কেন্দ্রের অবস্থা কেমন বলে আপনি মনে করেন?

২. আপনার মতে, গত পাঁচ বছরে আপনার এলাকার স্বাস্থ্য সেবার মান কি একই রকম আছে নাকি ভালো অথবা খারাপ হয়েছে? (কেন/কিভাবে)

৩. এই এলাকার প্রধান স্বাস্থ্য সমস্যা কি বলে আপনি মনে করেন? (সবচেয়ে বেশি কি রোগ/অসুস্থতা দেখা গিয়েছে)

৪. স্বাস্থ্য সেবার ক্ষেত্রে আপনার ভুমিকাকে আপনি কিভাবে ব্যাখ্যা করবেন?

- এই বিষয় নিয়ে কাজ করার কোন অভিজ্ঞতা আছে কি?
- আপনার প্রভাব বা অবদান কতটুকু?
- আপনি কি মনে করেন এই ক্ষেত্রে আপনার কোন দায়িত্ব বা করনীয় আছে?
- এই এলাকায় কোন তিনটি স্বাস্থ্য সমস্যাকে আপনি সবচেয়ে বড় সমস্যা বলে আপনি মনে করেন?

৫. এই এলাকায় স্থানীয় সরকার এবং স্বাস্থ্য ক্ষেত্রের মধ্যে কি কোন ধরনের সমন্বয় আছে?

- হ্যাঁ হলে, সেটা কেমন?
- না হলে, কি কারনে এই সমন্বয় হয় না?

৬. আপনি কি কখনও স্থানীয় পর্যায়ের পরিকল্পনা মিটিং এ অংশগ্রহন করেছেন?

- হ্যাঁ হলে, ঐ মিটিং থেকে আপনি কি ধরনের ফলাফল আশা করেন?
- না হলে, কখন্ও কি এই ধরনের মিটিং এর কথা শুনেছেন?

৭. এই এলাকার মানুষজন অসুস্থ হলে সেবার জন্য সাধারনত কোথায় যায় এবং তাদের অভিজ্ঞতা কেমন?

- প্রধান প্রধান সেবাদান কেন্দ্রে কি সবাইকে সমানভাবে চিকিৎসা সেবা দেওয়া হয়?
- সেবাকেন্দ্রগুলো কি সুবিধাজনক জায়গায় অবস্থিত?

৮. আপনার কি মনে মনেহয়, সরকারি স্বাস্থ্য সেবা কেন্দ্রগুলোতে যথাযথ সেবা প্রদানের জন্য যথেষ্ট সংখ্যক জনবল, ওষুধ এবং অন্যান্য সুবিধাদি রয়েছে?

৯. সেবা কেন্দ্র থেকে সেবা নেওয়ার ব্যাপারে এলাকার মানুষকে সচেতন এবং উদ্বুদ্ধ করার ব্যাপারে আপনি কি ধরনের ভুমিকা পালন করে থাকেন?

১০. মানুষকে সচেতন এবং উদ্বুদ্ধ করার ব্যাপারে আপনার এলাকায় কি কোন ধরনের কর্মসূচি কি কখন্ও দেখেছেন?

১১. কোন সংগঠন এই ধরনের কাজ করে থাকে?

১২. এই এলাকায় এডভোকেসি করার জন্য কোন ধরনের সংগঠন এবং কি ধরনের কাজ করে থাকে?

- সুবিধা বঞ্চিত মানুষদের জন্য কি করা হয়? কোন ব্যাপারগুলো মানুষকে অনুপ্রানিত করে?
- কোন সংগঠনগুলো সুবিধা বঞ্চিত মানুষদেও নিয়ে কাজ করে?
- বিভিন্ন এ্যাডভোকেসি দলগুলো কি একসাথে কাজ করে? কিভাবে?

১৩. এলাকার মানুষদের মধ্যে পারস্পরিক সম্পর্ককে আপনি কিভাবে ব্যাখ্যা করবেন? (সহানুভতিপ্রবণ নাকি বিদ্বেষপূর্ণ; ব্যাখ্যা করুন)

১৪. এলাকার স্বাস্থ্য সেবার উন্নয়নে প্রধান বাধাসমূহ কি বলে আপনি মনে করেন?

১৫. এই বাধা/সমস্যাগুলো দূর করার জন্য কি করা যেতে পারে আপনি মনে করেন?

- সুনির্দিষ্ট পদক্ষেপ, পলিসি বা ফান্ড যাতে করে স্বাস্থ্য কেন্দ্রের মান উন্নয়ন করা যায়?

১৬. এলাকার মানুষ যাতে বেশি করে স্বাস্থ্য কেন্দ্র থেকে সেবা নেয় এবং ডেলিভারি করায় সে ব্যাপারে জনগণকে সচেতন করার ব্যাপারে আপনার কি করনীয় আছে বলে আপনি মনে করেন?

১৭. আপনি কি মনে করেন স্বাস্থ্য কেন্দ্রের পরিবেশ বা সবাইকে সমান সেবা দেওয়া এ ব্যাপারে কোন ভুমিকা রাখতে পারে?

১৮. স্বাস্থ্য কেন্দ্রে সেবার মান বাড়ানোর ক্ষেত্রে সবচেয়ে কার্যকরী পদক্ষেপ কি বলে আপনি মনে করেন?

১৯. স্বাস্থ্য সেবার ক্ষেত্রে কোন নতুন উন্নয়ন বা পদক্ষেপ সম্পর্কে কি আপনি কিছু শুনেছেন?

২০. দূর্গম এলাকার স্বাস্থ্যসেবা সংক্রান্ত কোন ধরনের পলিসি বা নির্দেশনা আছে কি?

- হ্যাঁ হলে, সে সম্পর্কে কি একটু বলবেন?
- না হলে, আপনি কি মনে করেন এরকম কিছু থাকা উচিৎ? (কেন)
- সেখানে কোন বিষয়গুলো অনর্ভূক্ত থাকা উচিৎ বলে আপনি মনে করেন?

(গবেষনায় অংশগ্রহন করা এবং সময় দেয়ার জন্য আপনাকে ধন্যবাদ)

Tool 6: FGD guideline for Community People

Objective(s) of the interview:

1. Understand the availability and accessibility of health facilities (number, distance, cost, transport)
2. Understand the care practices of the area (Typical knowledge, attitudes, practices, and resources)
3. Understand the experience of care seeking from health facilities (services available, accessibility to services)
4. Explore the status of social capital among peer groups.
5. Determine which are perceived to be a challenge for achieving optimal health service and the interrelationships among these constraints.
6. Suggestion to improve services.

Guiding questions

1. Which health services/health facilities are available in your area? (Probe: UH&FWC, USC, CC, UHC, Private Clinic, TBA, VD)
2. Which service provider/ health facility is more popular to villagers?
    - Why popular? (Probe: Social context, better service, less expensive, trust, especial reason)
    - In which places people are going less what are the reasons? (Probe: distance, expensive, lack of proper care, distrust)
3. Usually which transports usually people use to go to different health facilities?
4. If the urgency of ambulance arises is it possible to get at that moment?
    - If no; what may be the alternate vehicle? (Rickshaw, van, other)
    - Is other vehicles are available all the time or have to have arrangement earlier?
    - Are people/neighbors help to manage transportation during emergency? (if yes, how)
5. Where do pregnant women usually go for check-up?
    - If yes, Where? (GoB health facility, Private hospital, other)
    - Who suggests for that? (Relatives, community health care provider)
    - How many times went?
    - What situation they have to face to go for check-up? (No problem, transportation, financial, family members' resistance)
    - Do you think regular check-up is necessary and helpful? (if yes, what)
    - Did you get any benefit due to regular check up? (if yes, what)
    - If no, why most of pregnant women don't go for check-up? (Financial, religious, resistance from family member, health care provider came home)
6. In which place most of the babies delivered in this area? (Probe: Home, GoB health facilities, private hospitals)
    - Why in that place? (Less expensive, trust, family tradition, social reason, better service, especial reason)
7. Why did most of the people choose that place/person for delivery?
8. Who usually decide to go to that person/place for delivering? (Probe: Mother, husband, other family member)
9. (If went to health center) What situation people usually have to face to reach the facility? (Probe: Transportation, finance, company)
10. What happen just after reaching the health facility? (Probe: Admission, starting time of service providing, behavior of facility's staffs, expenditure)
11. Does anyone come to see mother & baby after delivery or people bring the baby to any health facility?
12. If any baby becomes sick then where do people usually go for treatment?
    - Why people go to that person/place more? (Probe: especial reason, cost effective, trust)

13. Who usually take decision about choosing person/place for seeking care for baby? (Probe: Mother, husband, other family member)
14. Does the EPI camp arranged regularly in your area?
15. What about vaccination of babies around your house?
16. From where/person you know about vaccine/EPI?
17. What is your opinion about overall health care services of your area?
18. Which things you feel is not up to mark/satisfactory level?
    - The issues you said are not satisfactory, in those which is most considerable?
19. You told about few problems; can you please suggest something for remedy of those problems? And how the level of service can be upgraded?
20. What types of groups are available in this area? (Probe: financial or social group)
21. Do those groups have any influence on your health care seeking behavior? (If yes how)
22. How will you describe the helpfulness of you neighbors to you and surrounding people? Why do you think so?
23. How people are involved in communal work like repairing roads etc?
24. What is the history of building community clinic of your area? (Probe: land donor, building process)
25. If anyone becomes sick, how neighbors help/support that household/person? (Probe: financial, mental, transportation, other)
26. What are the main sources of information about health care services? (TV, internet, mobile phone, others)
27. What is the role of local political & religious leaders to solve different types of problems of the locality?
28. If any poor person/household needs financial help due to sickness, do local leaders or community people help that person? (If yes, how)

THANK YOU for taking the time to participate in this interview.

Objective(s) of the interview:

1. Understand the availability and accessibility of health facilities (number, distance, cost, transport)
2. Understand the care practices of the area (Typical knowledge, attitudes, practices, and resources)
3. Understand the experience of care seeking from health facilities (services available, accessibility to services)
4. Explore the status of social capital among peer groups.
5. Determine which are perceived to be a challenge for achieving optimal health service and the interrelationships among these constraints.
6. Suggestion to improve services.

Guiding questions

১. আপনাদের এলাকায় কি কি ধরনের স্বাস্থ্য কেন্দ্র/সেবা পাওয়ার সুযোগ আছে? (ইউনিয়ন স্বাস্থ্য ও পরিবার পরিকল্পনা কেন্দ্র, ইউনিয়ন সাব-সেন্টার, কমিউনিটি ক্লিনিক, উপজেলা স্বাস্থ্য কমপ্লেক্স, প্রাইভেট ক্লিনিক, গ্রাম্য ডাক্তার, ধাত্রী)

২. গ্রামে কোন মানুষ অসুস্থ হলে সাধারনত কার কাছে/কোথায় যায়?

- কেন ঐসব জায়গাতেই যায়? (প্রোবঃ সামাজিক কারণ, উন্নত সেবা, বিশেষ কারন, কম টাকা খরচ হয়, বিশ্বাস-আস্থা)
- যেসব জায়গায় কম যায় তার পেছনে কারণ কি? (প্রোবঃ দুরত্ব, বেশি টাকা খরচ হয়, যথাযথ সেবা পাওয়া যায় না, আস্থা নাই)

৩. বিভিন্ন স্বাস্থ্য সেবা কেন্দ্রে যাওয়ার জন্য মানুষ সাধারত কি ধরনের যানবাহন ব্যবহার করে?

৪. হঠাৎ করে যদি কারো এ্যম্বুলেন্স দরকার হয় তাহলে কি তা পাওয়া সম্ভব?

- যদি না হয়, তাহলে বিকল্প হিসাবে কি ব্যবস্থা নেয়া হয়? (প্রোবঃ ভ্যান-রিক্সা, অন্য কোন যানবাহন)
- অন্যান্য যানবাহন কি যে কোন সময় চাইলেই পাওয়া যায় নাকি আগে থেকে ঠিক করে রাখতে হয়? (পুরো ব্যাপারটি বোঝার চেষ্টা করুন)
- যানবাহন এর ব্যবস্থা করার ক্ষেত্রে কি এলাকার মানুষের সহযোগিতা পা্ওয়া যায়? (হ্যাঁ হলে, কি ধরনের)

৫. গর্ভবতী মায়েরা কি সাধারনত চেক আপ করানোর জন্য কোথায় যান?

- হ্যাঁ হলে, কোথায়? (প্রোবঃসরকারি স্বাস্থ্য কেন্দ্রে, বেসরকারি হাসপাতালে)
- কার পরামর্শে? (আত্মীয়, স্বাস্থ্যসেবা প্রদানকারী)
- যাতায়াতের সময় কি ধরনের পরিস্থিতির সম্মুখীন হয়? (প্রোবঃ সমস্যা হয় না, যোগাযোগ ব্যবস্থা, আর্থিক সমস্যা, পরিবারের কারো বাধা)
- নিয়মিত চেক-আপ করিয়ে কি কোন উপকার হয় বলে মনে করেন? (হ্যাঁ হলে, কি ধরনের)
- না হলে, কি কারনে বেশিরভাগ মায়েরা চেক আপ করাতে চান না? (প্রোবঃ আর্থিক সমস্যা, পরিবারের কারো বাধা, ধর্মীয় গোড়ামী, সেবাদানকারী বাড়ি এসে দেখে যায়)

৬. এলাকার বেশিরভাগ বাচ্চার প্রসব কোথায় করানো হয়? (প্রোবঃ বাড়িতে, সরকারি স্বাস্থ্য কেন্দ্রে, বেসরকারি হাসপাতালে)

- উল্লেখিত জায়গায় কেন বেশি করানো হয়? (প্রোবঃ বিশেষ কারন, কম টাকা খরচ হয়, বিশ্বাস-আস্থা, পারিবারিক প্রথা, সামাজিক কারণ, উন্নত সেবা)

৭. কেন উনার দ্বারা ডেলিভারি / উক্ত স্থানে বেশি করানো হয়?

৮. বাচ্চা কোথায় জন্মাবে এ ব্যাপারে সিদ্ধান্ত গ্রহণ করে কে? (প্রোবঃ মা নিজে, স্বামী, পরিবারের অন্য কেউ)

৯. স্বাস্থ্য কেন্দ্রে যাওয়ার ক্ষেত্রে সাধারনত কি ধরনের অবস্থার সম্মুখীন হতে হয়? (প্রোবঃ যানবাহন, টাকা-পয়সা, সঙ্গী)

১০. স্বাস্থ্য কেন্দ্র পৌছানোর পর সাধারনত কি হয়? (প্রোব: ভর্তি হওয়া, সেবা প্রদান শুরু করা, সেবা প্রদানকারীদের আচরন, অর্থ খরচ)

১১. বাচ্চার জন্মের পরে কি কোন সেবাদানকারী বাড়িতে দেখতে আসে?

১২. কোন বাচ্চা অসুস্থ হলে সাধারনত চিকিৎসার জন্য কোথায় নেয়া হয়?

- উল্লেখিত জায়গায় কেন বেশি নেয়া হয়? (প্রোব: বিশেষ কারন, কম টাকা খরচ হয়, বিশ্বাস-আস্থা)

১৩. বাচ্চার চিকিৎসা কোথায় করানো হবে সে সিদ্ধান্ত সাধারনত কে নেন? (প্রোব: মা নিজে, স্বামী, পরিবারের অন্য কেউ)

১৪. আপনাদের এলাকায় কি বাচ্চাদের টিকা দেওয়ার জন্য নিয়মিত ক্যাম্প হয়?

১৫. আপনাদের বাড়ির আশেপাশের বাচ্চার সবাই কি সময়মতো টিকা নিয়েছে?

১৬. আপনারা কার কাছ থেকে টিকা দেয়ার সময়/স্থানের ব্যাপারে জানতে পারেন?

১৭. আপনার এলাকার সার্বিক স্বাস্থ্য সেবার অবস্থা সম্পর্কে আপনার মতামত কি?

১৮. কোন বিষয়গুলো আপনার কাছে অসুবিধাজন মনে হয়? (প্রোব: দুরত্ব, খরচ, সঙ্গী, অন্যান্য)

- যে অসুবিধাগুলোর কথা বললেন তার মধ্যে কোনটি সবচেয়ে মারাত্মক বলে আপনি মনে করেন এবং কেন?

১৯. আপনি বেশ কিছু অসুবিধার কথা বলেছেন, কি করলে এই সব অসুবিধাগুলো দূর করা সম্ভব এবং স্বাস্থ্যসেবার মান আরো উন্নত করা সম্ভব বলে আপনি মনে করেন?

২০. আপনাদের এখানে কি কি কোন ধরনের সমিতি বা সামাজিক সংগঠন আছে? (ঋণদানকারী, স্বোচ্ছাসেবী)

২১. এই সমিতি বা সংগঠন কি আপনাদের স্বাস্থ্যসেবা গ্রহনের ক্ষেত্রে কেন ভুমিকা রাখে? (হ্যাঁ হলে কিভাবে)

২২. আপনার এলাকার মানুষ অন্যদের ব্যাপারে কি পরিমান উপকারি/সাহায্যকারী বলে মনে করেন? কেন এমন মনে করেন?

২৩. এলাকার কোন কাজ করার সময় মানুষজন এর অংশগ্রহন কেমন থাকে? (রাস্তা নির্মান, বাঁধ নির্মান, স্বাস্থ্যকেন্দ্র নির্মান/মেরামত)

২৪. আপনারদের এলাকায় যে কমিউনিটি ক্লিনিকটি আছে সেটি নির্মান করা হয়েছিলো কিভাবে? (জমিদাতা, নির্মান প্রক্রিয়া)

২৫. কেউ অসুস্থ হয়ে পড়লে প্রতীবেশিরা কি ধরনের সহযোগিতা করে থাকে? (আর্থিক, মানসিক, যোগাযোগ)

২৬. স্বাস্থ্যসেবা সহ বিভিন্ন ধরনের তথ্য আপনারা কি মাধ্যমে জানতে পারনে? (টিভি, ইন্টারনেট, মোবাইল, অন্যান্য)

২৭. এলাকার বিভিন্ন ধরনের সমস্যা সমাধানে স্থানীয় রাজনৈতিক বা সামাজিক নেতাদের ভুমিকা কেমন? (প্রয়োজনে সাহায্য পাওয়া যায়, কোনো খোঁজ রাখে না, সমস্যা জানালে মাঝে মাঝে সাহায্য করেন)

২৮. কোন দরিদ্র মানুষের যদি স্বাস্থ্যসেবার জন্য অর্থের প্রয়োজন হয় তাহলে কি তারা নেতা বা এলাকার সাধারন মানুষের কাছ থেকে সাহায্য পায়? (হ্যাঁ হলে, কি ধরনের)

(গবেষনায় অংশগ্রহন করা এবং সময় দেয়ার জন্য আপনাকে ধন্যবাদ)
