# Supplementary material for: Bottleneck analysis of maternal and newborn health services in hard-to-reach areas of Bangladesh using ‘TANAHASHI’ framework’: An explanatory mixed-method study
Source: PLoS One. 2022 May 12;17(5):e0268029. doi: 10.1371/journal.pone.0268029 (PMC9098042; doi:10.1371/journal.pone.0268029)
Supplement: S4 File — (DOCX) [file pone.0268029.s004.docx]

**Supplementary table 1: Indicator matrix for quantitative data collection**

| **Building blocks** | **Availability** | **Accessibility** | **Utilization** | **Adequate coverage** | **Effective coverage** | **Data collection method** |
| --- | --- | --- | --- | --- | --- | --- |
| Service delivery | Number of FWV including CSBA who provide ANC services per 10000 population | Proportion of women aged 15-49 who had a live birth in past one year, lives within 1 hour travel to UH&FWC/ union sub-centre | Proportion of women aged 15-49 years with a live birth in past one year, received antenatal care from a skilled provider during their last pregnancy | Proportion of women aged 15-49 years with a live birth in past one year, received 4+ ANC from a skilled provider during their last pregnancy | Proportion of women aged 15-49 with a live birth in past one year, received 4+ antenatal care (ANC) visits and 450 calcium supplements (3 tab/day from 20 week gestation until the delivery) during their last pregnancy | Health facility assessment survey & household survey |
|  | Proportion of days in the last 3 months with suffcient stock of IFA in the facility | Proportion of women aged 15-49 who had a live birth in past one year, lives within one hour travel to facilities where IFA is available | Proportion of women aged 15-49 who had a live birth in past one year, received IFA during their last pregnancy | Proportion of women aged 15-49 who had a live birth in past one year, received sufficient IFA tablets for recommended 180 days during their last pregnancy | Proportion of women aged 15-49 who had a live birth in past one year, consumed IFA tablets for 180 days during their last pregnancy | Health facility assessment survey & household survey |
|  | Number of functional BEmONC facilities available per upazilla | Proportion of women aged 15-49 years who had a live birth in past one year , lives within 1 hour travel to BEmONC facilities | Proportion of women aged 15-49 years who had a live birth in past one year, delivered in the BEmONC facility | Proportion of women aged 15-49 years who had a live birth in past one year, delivered by medically trained provider in the BEmONC facility | Proportion of women aged 15-49 years who had a live birth in past one year, delivered by medically trained provider and delivery was conducted as per SOP for normal delivery in the BEmONC facility | Health facility assessment survey & household survey |
|  | Proportion of FWV including CSBA who provide PNC services per 10000 population | Proportion of women aged 15-49 who had a live birth in past one year, lives within one hour travel to UH&FWC/ union sub-centre | Proportion of women aged 15-49 years who had a live birth in past one year, received at least one PNC for their last born child from a skilled provider within 45 days of their delivery | Proportion of women age 15-49 who had a live birth in past one year, received 2+ PNC for their last born child from a skilled provider within 45 days of their delivery | Proportion of women aged 15-49 years who had a live birth in past one year, who received 2+ PNC visits for their last born children within 45 days of their delivery and post natal vitamin A supplementation | Health facility assessment & household survey |
|  | Proportion of FWV including CSBA who provide PNC services per 10000 population | Proportion of women aged 15-49 years who had a live birth in past one year, have access within 2 kms to UH&FWC/ union sub-centre | Proportion of women aged 15-49 years who had a live birth in past one year, received at least one PNC for themselves from a skilled provider within 45 days of their delivery | Proportion of women aged 15-49 years who had a live birth in past one year, received 2+ PNC for themselves from a skilled provider within 45 days of their delivery | Proportion of women aged 15-49 years who had a live birth in past one year, received 2+ PNC visits for themselves within 45 days of their delivery and post natal vitamin A supplementation | Health facility assessment survey & household survey |
| Health workforce | Proportion of facilities with position for health workforce categories to deliver ANC , BEmONC / CEmONC, ENC & PNC services | | Proportion of facilities with position filled for health workforce categories to deliver ANC , BEmONC / CEmONC, ENC & PNC services | Proportion of facilities with presence of the filled up positions for health workforce categories to deliver ANC , BEmONC / CEmONC, ENC & PNC services at the day of assessment | | Health facility assessment survey |
|  | Proportion of health workers have provision of receiving training on ANC , IMPAC, AMTSL, EOC, Post abortion care & neonatal resuscitation within past 24 months | | Proportion of health workers have received in service training on ANC , IMPAC, AMTSL, EOC, Post abortion care & neonatal resuscitation within past 24 months | Proportion of health workers have provision of receiving training received in service training and practice the gained knowledge effectively during job on ANC , IMPAC, AMTSL, EOC, Post abortion care & neonatal resuscitation within past 24 months | | Health facility assessment survey |
| HMIS | Availability of registers for ANC, delivery, PNC and neonatal case management | | Utilization of the registers | Proper maintenance of the registers with filling up all the required fields | | Health facility assessment survey |
|  | Provision of generation of report regarding MNH services to HMIS | | Practice of sending online reports regarding MNH services to HMIS | Accurate, timely and complete report generated for ANC, delivery, PNC and neonatal case management | | Health facility assessment survey |
| Access to essential medicine | Proportion of facilities with available priority medicines for mothers and newborn defined by WHO | | | | | Health facility assessment survey |
| Health financing | General government expenditure on health as proportion of general government expenditure | | | | | Health facility assessment survey and document review |
|  | The ratio of household out of pocket expenditure for health to total expenditure on health | | | | | Household survey |
| Leadership / Governance | Proportion of facilities with available ANC , BEmONC/ CEmONC, ENC & PNC guideline | | | | | Health facility assessment survey |
|  | Proportion of facilities having internal supervision and monitoring system | | | | | Health facility assessment survey |
