# Supplementary material for: Bottleneck analysis of maternal and newborn health services in hard-to-reach areas of Bangladesh using ‘TANAHASHI’ framework’: An explanatory mixed-method study
Source: PLoS One. 2022 May 12;17(5):e0268029. doi: 10.1371/journal.pone.0268029 (PMC9098042; doi:10.1371/journal.pone.0268029)
Supplement: S5 File — (DOC) [file pone.0268029.s005.doc]

.

| **Theme** | **Sub-theme** | **A-priori Code** |
| --- | --- | --- |
|  |  |  |
| Availability & Accessibility of Health Facilities | Availability | Number of health facilities (HF) |
|  |  | Available service providers |
|  |  | Available services |
|  |  | Availability of ambulance/transports |
|  |  | HR in facilities |
|  |  | Medicine in facilities |
|  | Accessibility | Distance of HF |
|  |  | Time requires to reach |
|  |  | Mode of transport |
|  |  | Cost of transport |
| Care seeking practices & experience of the area | Provider Choosing | Reason of choosing |
|  |  | Reason for not choosing |
|  | Difficulties of reaching HF | Availability of transports |
|  |  | Availability of companion |
|  |  | Economical barrier |
| ANC | Place of ANC | Place |
|  |  | Provider |
|  |  | Reason |
|  | Influence | Advised by |
|  |  | Resistance from family member |
|  | Time | How many times |
|  |  | Months of pregnancy |
|  | Benefit | Useful |
|  |  | Not necessary |
|  | Difficulties of reaching HF | Availability of transports |
|  |  | Availability of companion |
|  |  | Economical barrier |
| Delivery | Place of delivery | Place of others’ |
|  |  | Reason |
|  |  | Place-own |
|  |  | Delivery person |
|  | Decision making | Decision maker |
|  |  | Resistance from family member |
|  |  | Reason of the decision |
|  | Difficulties of reaching HF | Availability of transports |
|  |  | Cost of transport |
|  |  | Cost of Delivery |
|  |  | Availability of companion |
|  |  | Economical barrier |
|  |  | Source of economic |
|  | After reaching HF | Admission |
|  |  | Attitude of the service provider |
|  |  | Satisfaction about service |
|  | Especial assistance | Admission |
|  |  | Economical |
|  | Complications | Eclampsia |
|  |  | Pre-term baby |
|  |  | Excessive bleeding |
|  | Complications management | KMC, incubator, others |
|  |  | Blood managing |
| PNC | Check up | Home |
|  |  | Frequency at home |
|  |  | Facility |
|  |  | Frequency to facility |
|  | Difficulties of reaching HF | Availability of transports |
|  |  | Availability of companion |
|  |  | Economical barrier |
|  | After reaching HF | Admission |
|  |  | Attitude of the service provider |
|  |  | Satisfaction about service |
| Child/neonatal care | Place | Where |
|  |  | Provider |
|  | Decision making | Decision maker |
|  |  | Reason the decision |
|  | Difficulties of reaching HF | Availability of transports |
|  |  | Availability of companion |
|  |  | Economical barrier |
|  |  |  |
|  | Vaccination | Regular EPI camp |
|  |  | Distance of EPI center |
|  |  | Environment of the center |
|  |  | Attitude of the service provider |
|  |  | Information received by |
|  |  | Other babies situation |
| Overall impression about health care services | Overall situation | Opinion |
|  |  | Main health problem |
|  |  | Advancement of HC in 5yrs |
|  | Problems | Distance |
|  |  | Cost |
|  |  | Companion |
|  |  | Road communication |
|  |  | Most significant problem |
|  | Recommendation | Suggestions |
|  | Co operative | Microcredit |
|  | Community help | Emergency loan |
|  |  | Other help |
|  | Health profession involve | relative |
