# Supplementary material for: Bottleneck analysis of maternal and newborn health services in hard-to-reach areas of Bangladesh using ‘TANAHASHI’ framework’: An explanatory mixed-method study
Source: PLoS One. 2022 May 12;17(5):e0268029. doi: 10.1371/journal.pone.0268029 (PMC9098042; doi:10.1371/journal.pone.0268029)
Supplement: Attachment — Submitted filename: Plos review_7 Jan.docx [file pone.0268029.s006.docx]

Abstract: Authors might want to write it in structured format.

The authors wrote “Bangladesh being a low-and-middle income country, set targets for MNH aligning with Sustainable 24 Development Goals” ---how does a country’s income level matters here? Rewrite this sentence, I think it does not quite make sense to bring that ‘low-and-middle income’ category as a reason to set SDG targets.

Introduction

- “342 to 211 per 100,000 live births between 2000-2017, still over 800 women are dying from pregnancy and childbirth related complications”—where are these women dying and how frequently? Please specify a country, region or worldwide—where? And annually or over a certain period of time?
- Can authors clearly demonstrate the gap in a table/box? They might want to bring the annual rate of decline in MMR and NMR over the past (e.g. 1990 to 2017). Also include in the table the numbers on where we reach provided the similar rates of decline by 2030, and the rates required to reach the SDG targets.
- Can authors also talk about the gaps in health service coverage nationally vs in HtR areas, both for key maternal and neonatal interventions?
- The detailed description of Tanahashi framework might be omitted from introduction. Authors might want to demonstrate the figure in method section.

Materials and methods

- Did the authors go for 100% of the villages in the 4 HtR district? It is not clear.
- Use a reference for ‘explanatory sequential mixed-method design’.
- How did the data saturation work for different categories of participants? Having 1-2 sentences about authors’ practical insight into it could be useful.
- How was the ‘availability’ determined by interviews or observation of the records from all health facilities of the 4 districts? Please confirmed it. Also, how did you explore clients’ need? How did you define the actual volume of the clients in the study location?
- Please mention the total lists of unions in each HtR district? What is your reason to choose 3 unions from each HtR district?
- How did you define RDW? Is 2989 the all RDWs in the 36 villages?
- Did you select all facility managers and providers in the study areas? If not how did you select them for the interviews? You used snowball for local stakeholders, but how did you get the other qualitative participants in your study?
- In the tools section, you said you had IDIs with RDWs? And FGDs with community people? Can you mention how many IDIs and FGDs conducted in the method above?
- I would suggest you make a simple summary table with type of data collection methods, type of study participants, type of data collection tools, number of participants to make it easier for the readers to understand the types of data and different participants in your study.
- Your overall sampling process for quanti looks like ‘multi-stage convenient sampling’.
- In variable section, your key variables are those stated in Tanahashi framework. Please provide your working definition (clearly mention the operational definition used in your analysis) availability, accessibility, utilisation, and others. I would suggest a simple table again to list each sub variables by those key domain variables (availability, accessibility, utilisation, Adequate coverage and Effective coverage).
- Submit as additional files of all of your data collection tools.

Analysis

- Mention the key descriptive stats utilised.
- Mention the number of transcripts and people involved in analysing qualitative data.
- How did you check inter coder reliability as stated?
- Upload the primary code book and the master code list file as additional files.
- Provide a reason (maybe on method section above) on why FGDs, IDIs, KIIs—all of these needed in your study? There seems a big bulk of data—was it all necessary? If so why, for which aspects of your study? How did your principle of saturation worked out while you were collecting data using these many different methods?

Ethics

- Were the written consents taken from RDWs? There are nearly 15% who had never been to school as per your data. How did it work?
- Did you exclude any categories of RDWs for any ethical reasons? Such as those who had lost their babies recently??
- Did you collect written consents for all qualitative participants too? How did it work for FGDs?

Results

- Can you give a brief description on which level of the 29 health facilities are in your health system? You said only 13.7% were providing CS---were all 29 facilities eligible to provide CS as per your Ministry/Department of Health? The same for blood transfusion ! How many of the 29 facilities are Comprehensive Emergency Obstetric and Newborn Care providers?
- For each component analysis—how does a reader understand how many were supposed to provide that particular care component, say ‘Antenatal care’? For the HR it is clear as you mention the sanctioned posts.
- I would strongly suggest reorganizing the results as per the Tanahashi domain variables—be specific to mention your results as per the key domain variables of the framework you utilised. You can mention the socio-demographic characteristics of study participants—of both quantitative and qualitative participants at the start of the result section. In so doing, you can bring in the key qualitative information integrating together with the key domains of the Tanahashi model.
- Provide a list of key bottlenecks as per each domain in the model, maybe do so in a table. You have collected a lot of qualitative data—I think there could be plenty of info on bottlenecks.

Discussion

Needs further work. Once you have reorganized the result section well as per the model you used—you then can choose key points to interpret, elaborate and provide compelling discussion. Also, acknowledge the limitation of the convenient sampling you have utilised, and strengths of the range of data collection methods employed.

Conclusion: Authors need to write a brief conclusion separately.

Thank you for the great work.
